# Structural and spectroscopic characterization of a Tb(IV) polyoxometalate

**DOI:** 10.1038/s41467-026-75657-7

**Published:** 2026-07-16

**Authors:** Primadi J. Subintoro, Brett Lottes, Felipe A. Pereiro, Nolwenn Mahieu, Alexander M. Brown, S. Genevieve Duggan, Monica S. Mullis, Aldo M. Jordan, Cassandra Gates, Samuel M. Greer, Joshua J. Woods, Rebecca J. Abergel, Jenifer C. Shafer, Daniel K. Unruh, Pere Miró, Benjamin W. Stein, Jennifer N. Wacker, Stosh A. Kozimor, Korey P. Carter

**Affiliations:** 1 Department of Chemistry, University of Iowa, Iowa City, IA USA; 2 Chemistry Division, Los Alamos National Laboratory, Los Alamos, NM USA; 3 Department of Chemistry, Colorado School of Mines, Golden, CO USA; 4 Chemical Sciences Division, Lawrence Berkeley National Laboratory, Berkeley, CA USA; 5 Department of Nuclear Engineering, University of California, Berkeley, CA USA; 6 Department of Chemistry, University of California, Berkeley, CA USA; 7 Office of the Vice President for Research, University of Iowa, Iowa City, IA USA

**Keywords:** Chemical bonding, Coordination chemistry, Inorganic chemistry

## Abstract

There has been a renaissance in high valent lanthanide chemistry, which has resulted in the first examples of tetravalent praseodymium (Pr) and terbium (Tb) in molecular systems. These feats have been achieved with tailored ligands that facilitate tetravalent lanthanide stability in non-aqueous conditions. The next step in realizing the potential of high valent lanthanide chemistry is moving towards complexes that are stable in ambient and aqueous conditions. In this investigation, we advance this paradigm by obtaining definitive evidence of Tb(IV) in a molecular system under aqueous conditions utilizing the lacunary Wells-Dawson polyoxometalate, K_10_P_2_W_17_O_61_•20H_2_O. Herein we present the single crystal structure of K_16_Tb(IV)(P_2_W_17_O_61_)_2_•39.85H_2_O (Tb(IV)W_34_) as well as extensive spectroscopic evidence obtained via UV–Vis-NIR, X-ray absorption near edge spectroscopy, continuous wave X-band electron paramagnetic resonance spectroscopy and SQUID magnetometry measurements to confirm the oxidation state of Tb in W_34_ complexes as +4.

## Abstract (web summary)

Here, authors describe how polyoxometalate chemistry can overcome intrinsic redox and reactivity limitations in aqueous media to allow for structural and spectroscopic characterization of terbium in the +4 oxidation state.

## Introduction

A defining feature of lanthanide molecular chemistry is the persistence of metals in the +3 oxidation state^1–3^. There are historic exceptions in cerium (Ce), samarium (Sm), europium (Eu), and ytterbium (Yb), in which stability of the +4 or +2 oxidation states are rationalized by the closed shell (Ce(IV), Yb(II)) and half-filled (Eu(II) or nearly half-filled Sm(II)) electronic configurations^4–9^. More recently, pioneering work from the groups of Lappert, Evans, and others have demonstrated that Ln(II) chemistry can be realized across the whole series, which has significantly advanced understanding of 4f-element electronic structure^10–13^. Pursuit of high valent lanthanide species beyond Ce has been a longstanding research aim since the 1960s, with only a few successful examples in aqueous carbonate media or solid-state systems^14–17^. This paradigm changed with the recent works from the groups of La Pierre, Mazzanti and Zheng, which demonstrated that isolation and characterization of molecular complexes of tetravalent praseodymium and terbium was possible in non-aqueous, inert conditions^18–30^. In aqueous media, molecular examples of divalent and tetravalent lanthanides, beyond Ce, are rare as lanthanide metal ions are labile toward redox processes that lead back to the +3 oxidation state. We have overcome this limitation using polyoxometalate (POM) chemistry, and herein we present, K_16_Tb(IV)(α_2_-P_2_W_17_O_61_)_2_•39.85H_2_O (Tb(IV)W_34_), a molecular tetravalent terbium complex that can be synthesized in the presence of persulfate and characterized in water, although still susceptible to auto reduction upon desolvation.

Tb(IV) is a species of fundamental importance as it is the starting point for further explorations into higher oxidation state lanthanide chemistry, and its 4*f*^7^ electronic configuration is distinct, wherein quenching of the huge orbital angular momentum of the *f*-orbitals means it should have a purely spin based ground state of S = 7/2 analogous to Gd(III). La Pierre and colleagues used electron paramagnetic resonance (EPR) spectroscopy to investigate a series of three 4*f*^7^ (Eu(II), Gd(III), Tb(IV)) molecular analogues, and found that the electronic ground state of Tb(IV) was distinct compared to the Gd(III), due to the higher metal ligand covalency and spin orbit coupling of the tetravalent cation^31^. This raises the question of whether the magnetic and spin-based properties of Tb(IV) and Gd(III) are comparable, and to advance research in this regard, a fundamental understanding of the coordination chemistry that allows for Tb(IV) to be isolated must be developed. The current strategy for accessing tetravalent Tb and Pr, which has been explicitly outlined by the group of La Pierre and used by the groups of Mazzanti and Zheng, is based on the use of bulky ligands with strong σ and π donating sites that can destabilize the lanthanide *f*-orbitals, thereby lowering the energetic favorability of the trivalent configuration. The ligands used by all three groups also have the overall effect of increasing ligand covalent character such that tetravalent congeners are stabilized, and the thermodynamic cost of oxidation is diminished.

Manifestations of these strategies have resulted in significant negative shifts in metal-ion redox potentials as exemplified by the shifting of the E_0_ value for Ce(IV)/Ce(III) pair to −2.64 V vs. Fc/Fc^+^ (from +1.30 V) when complexed with tris(piperidinyl)imidophosphorane, [NP(pip)_3_]^-^, ligands^32^. However, siloxide and imidophosphorane ligands are not readily translatable to aqueous environments, yet we saw a number of similarities between these ligand systems and tungsten-based POMs ligands, which are a research focus within our group^33,34^. The lacunary Wells-Dawson (α_2-_P_2_W_17_O_61_)^10-^ (W_17_) anion, specifically, is known to preferentially bind tetravalent *f*-elements, and is also stable in water^35–38^. The four oxygens within the lacunary site of the W_17_ anion contain highly localized lone pairs, similar to those found in the siloxides and imidophosphorane ligands, while the bulky nature of the W_17_ ligands impose a rigid coordination environment that does not require rearrangement to accommodate tri- or tetravalent Ln cations. These similarities led us to believe that W_17_ ligands could stabilize Tb(IV) in aqueous media, which would then provide a platform to interrogate how changes in metal-ion oxidation states affect electronic and spin manifolds within POM complexes, thereby advancing efforts to use this class of materials in quantum information science and spintronic applications.

We are not the first to recognize the potential of W_17_ POMs for lanthanide coordination chemistry efforts as Saprykin et al. have documented the solution state complexation of Tb(IV) with W_17_ ligands almost half a century ago^39^. Fedoseev and colleagues have been the only group to pursue this chemistry further^35,40^; however, beyond the identification of a metal-based charge transfer band at 440 nm in UV–Vis-NIR spectra, no further characterization of tetravalent terbium in a TbW_34_ complex has been published. The hypothesis from these studies is that the preference of W_17_ POMs for tetravalent *f*-elements is driven by chelate effects and the electronic structure of the lacunary species. The four basic oxygens and the accessible 5 *d* orbitals of the W(VI) cations are thought to facilitate charge delocalization that favors binding with tetravalent species. Lanthanide and actinide M(IV)/(III) redox potentials are known to decrease by 1.2–1.3 V upon complexation with two W_17_ ligands^35,38^, and proton-independent stability constants (log *β*) for An(IV)/Ln(IV)W_34_ complexes are often more than twice the value of their An(III)/Ln(III) congeners^35^. A shift of the Tb(IV)/(III) redox potential by 1.2–1.3 V would bring this value to just below 2 V vs. NHE, and this reduction potential shift allows strong chemical oxidants such as persulfate (S_2_O_8_^2-^) to oxidize Tb(III) within the electrochemical window of water^35,39,40^.

Herein, we report the synthesis, structure, and spectroscopic characterization of a Tb(IV)W₃₄ POM complex. The species was prepared using a two-step process: first, a Tb(III) sandwich complex coordinated by W₁₇ ligands was synthesized; second, this precursor was subjected to a post-synthetic oxidation reaction, which converted Tb(III) to Tb(IV), yielding the desired product. The characteristic brown color of Tb(IV) is observed for Tb(IV)W_34_ complexes in both solution and single crystal samples, and this species was characterized in detail using single crystal X-ray diffraction (SCXRD), X-ray absorption (XAS), UV–Vis–NIR, and EPR spectroscopies as well as SQUID magnetometry measurements and density functional theory calculations. SCXRD experiments revealed Tb(III)W_34_ and Tb(IV)W_34_ species were isostructural, and comparison of average bond distances between coordinating oxygen atoms (d_Ln-O_) in the Tb(III)W_34_ and Tb(IV)W_34_ complexes revealed a ~ 0.1 Å decrease for the tetravalent complex. X-ray absorption near edge spectroscopy (XANES) measurements on Tb(IV)W_34_ featured a white line doublet that is characteristic of tetravalent terbium; however, the sample is unstable in the X-ray beam and auto-reduction back to Tb(III) was observed. UV–Vis–NIR spectra for Tb(IV)W_34_ feature a ligand to metal charge transfer (LMCT) band with a λ_max_ = 437 nm that is absent in the spectrum of the Tb(III)W_34_ complex. Longitudinal UV–Vis-NIR measurements on Tb(IV)W_34_ were conducted over a period of twelve days, and during the experimental observation period Tb(IV)W_34_ was stable when kept under closed aqueous conditions based on the sustained presence of the 437 nm charge transfer peak^40^. X-band EPR measurements on solid-state samples of Tb(IV)W_34_ further confirmed the Tb(IV) oxidation state assignment as spectra contained features that have only been observed for previously reported molecular Tb(IV) complexes^31^. Zero field cooled SQUID magnetometry measurements for Tb(IV)W_34_ revealed an effective magnetic moment (μ_eff_) value that is consistent with expectations for a *f*^7^, ^8^S_7/2_ metal-ion, which also contrasted with the magnetic behavior and μ_eff_ value for Tb(III)W_34_. Finally, density functional theory calculations for Tb(IV)W_34_ found that oxidation happens on the terbium center without involvement of the W_17_ ligands, which also leads to oxygen to terbium ligand-to-metal charge transfer in computed absorption spectra, consistent with experimental findings regarding the tetravalent character of Tb(IV)W_34_.

## Results and discussion

The synthesis of Tb(IV)W_34_ was performed under ambient conditions using persulfate (S_2_O_8_^2-^) as the chemical oxidant, as this was the only anion capable of oxidizing Tb(III) to Tb(IV) in the TbW_34_ complex. This observation is consistent with the results from Fedoseev and Shilov^35,40^, who suggested a 1.2–1.3 V decrease in the reduction potential of the Tb(IV)/Tb(III) pair occurs upon POM complexation (going from 3.1 V down to ~1.9 V vs. NHE), which is within the oxidative window of S_2_O_8_^2-^ (E°_S2O8/SO4_ = 2.01 V vs. NHE). The importance of the background electrolyte and the solution ionic strength for facilitating Tb oxidation in solutions containing Tb(III)W_34_ was also noted in the studies from Fedoseev and Shilov^35,40^, and these observations suggest that the oxidation mechanism for Tb ions in W_34_ complexes involves both the first coordination sphere and the components of the lattice. More specifically, it was hypothesized that Tb oxidation in W_34_ complexes occurs via a radical mechanism that only happens when Tb(III) is chelated by one W_17_ ligand and several aqua ligands, wherein chemical oxidants react with the aqua ligands to form hydroxyl radicals (OH•) that ultimately oxidize the Tb(III) center in TbW_34_^40^. Our experience preparing Tb(IV)W_34_ corroborated this hypothesis as we found that other chemical oxidants (i.e., alkali bromates, hydrogen peroxide, potassium permanganate, silver nitrate, potassium dichromate) were incapable of oxidizing Tb, while increasing ionic strength or counterion concentrations interrupted or impeded the ability of persulfate to interact with aqua ligands, resulting in a detrimental change to the kinetics of the oxidation of Tb(III) to Tb(IV) in the TbW_34_ system.

**Fig. 1:**
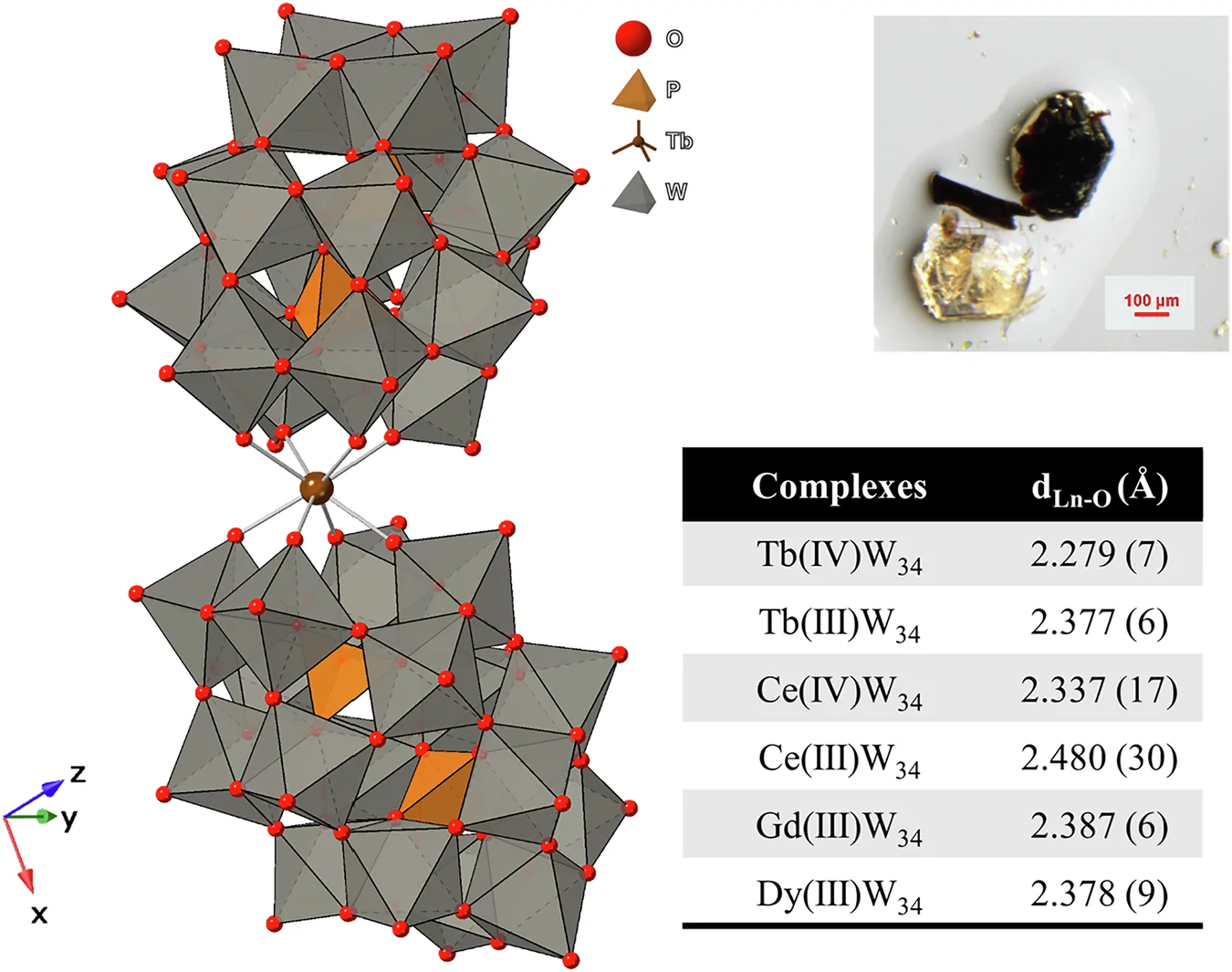
Single crystal X-ray diffraction was used to probe polyoxometalate structures in the solid-state. (Left) Polyhedral representation of local structure of Tb(IV)W_34_. Tb is depicted as a brown sphere, whereas W and P atoms are represented as gray and orange polyhedral, respectively. Oxygen atoms are represented as red spheres. Lattice water molecules and potassium counterions have been omitted for clarity. (Top Right) Picture of the reddish-brown crystals of Tb(IV)W_34_ and (Bottom Right) table of average Ln-O bond distances in Tb(IV)W_34_, Tb(III)W_34_, Ce(IV)W_34_, Ce(III)W_34_, Gd(III)W_34_, and Dy(III)W_34_.

Single crystal X-ray diffraction analysis revealed that Tb(IV)W_34_ (Fig. 1) crystallizes in the space group *C*2/*c* with crystallographic parameters included in Table S1 (Supplementary Information). The Tb(IV) cation is eight coordinate and adopts slightly distorted square antiprismatic (*D*_4*d*_) molecular geometry. This is a common structural motif in Peacock-Weakley type POM complexes featuring *f*-elements, Fig. 1, and here we note that two W_17_ ligands sandwich the Tb(IV) cation in a *syn* confirmation through four oxygen atoms from each lacunary Wells-Dawson POM^41,42^. The four coordinating oxygens from the W_17_ ligand are located in the lacunary sites of the POM ligands where a WO^4+^ subunit has been removed. These lacunary oxygens are stronger electron donors, in comparison with the bridging and terminal oxygens of the POM cluster^41,42^, and the oxygen atoms within the lacunary sites strongly interact with the harder Tb(IV) cation in Tb(IV)W_34_ versus the Tb(III) cations in Tb(III)W_34_, as reflected by the average Ln-O distance (d_Ln-O_) of 2.279(7) Å for Tb(IV)W_34_ and 2.377(6) Å for Tb(III)W_34_. The decrease in metal-oxygen bond length (Δd_Tb-O_ = 0.098 Å) when comparing Tb(IV)W_34_ and Tb(III)W_34_ is less than the contraction observed between Ce(III)W_34_ and Ce(IV)W_34_ (Δd_Ce-O_ = 0.14 Å) and less than the difference between CN = 8 ionic radii for Tb(III) and Tb(IV) (Δd = 0.16 Å)^43^. Based on these structural observations, it is possible that Tb(IV)W_34_ may have some mixed valent character or may have started to reduce during the XRD data collection process; however, the bond valence (BV) sums for Tb in Tb(IV)W_34_ and Tb(III)W_34_ are 4.11 and 3.16, respectively, (Table S2, Supplementary Information) which suggest that the oxidation state of Tb in Tb(IV)W_34_ is predominantly +4. Looking into the overall packing of Tb(IV)W_34_, the unit cell is different than what was observed for Ln(III)W_34_ complexes (Tb(III)W_34_, Ce(III)W_34_, Gd(III)W_34_, or Dy(III)W_34_) and tetravalent Ce(IV)W_34_, Table S1 (Supplementary Information). Interestingly, the packing of Ce(IV)W_34_ matches with what is observed for Ln(III)W_34_ congeners; however, when Ce(IV)W_34_ was synthesized following the two-step process used to prepare Tb(IV)W_34_, a secondary Ce(IV)W_34_ phase (Ce(IV)W_34__version II) was produced that packs in the same manner as Tb(IV)W_34_, Table S1 (Supplementary Information). This observation indicates the packing of Tb(IV)W_34_ is caused by the tailored reaction conditions, and that otherwise the Tb center in Tb(IV)W_34_ behaves as a typical tetravalent lanthanide cation.

We also evaluated the effective symmetry of the Tb(IV) center in Tb(IV)W_34_ via calculations of distortion parameters (DPs) associated with the square antiprismatic (*D*_4*d*_) symmetry of lanthanide metal centers within W_34_ complexes, Fig. S3, Supplementary Information. Comparing DP values between Tb(III)W_34_ and Tb(IV)W_34_ provides insights into the different coordination environments of Tb and allowed us to assess whether significant rearrangement is required to facilitate the oxidation of the Tb(III) center. The skew angle (SA), plane angle (PA), and plane distance (PD) for Tb(IV)W_34_ are 3.775 °, 1.78 °, and 0 Å, respectively. Comparing these values to those of Tb(III)W_34_ (SA = 3.03 °, PA = 4.2 °, PD = 0.025 Å) reveals only small changes in the DPs with the biggest difference in the PA values for the two complexes (Fig. S4, Supplementary Information). Overall, these slight differences in DP values translate into minor changes in the coordination environment between Tb(III) and Tb(IV) in W_34_ complexes, and this indicates no major rearrangement is required to accommodate Tb(IV); a key to enhancing the stability of tetravalent Tb as first highlighted by La Pierre et al*.*^19^.

**Fig. 2:**
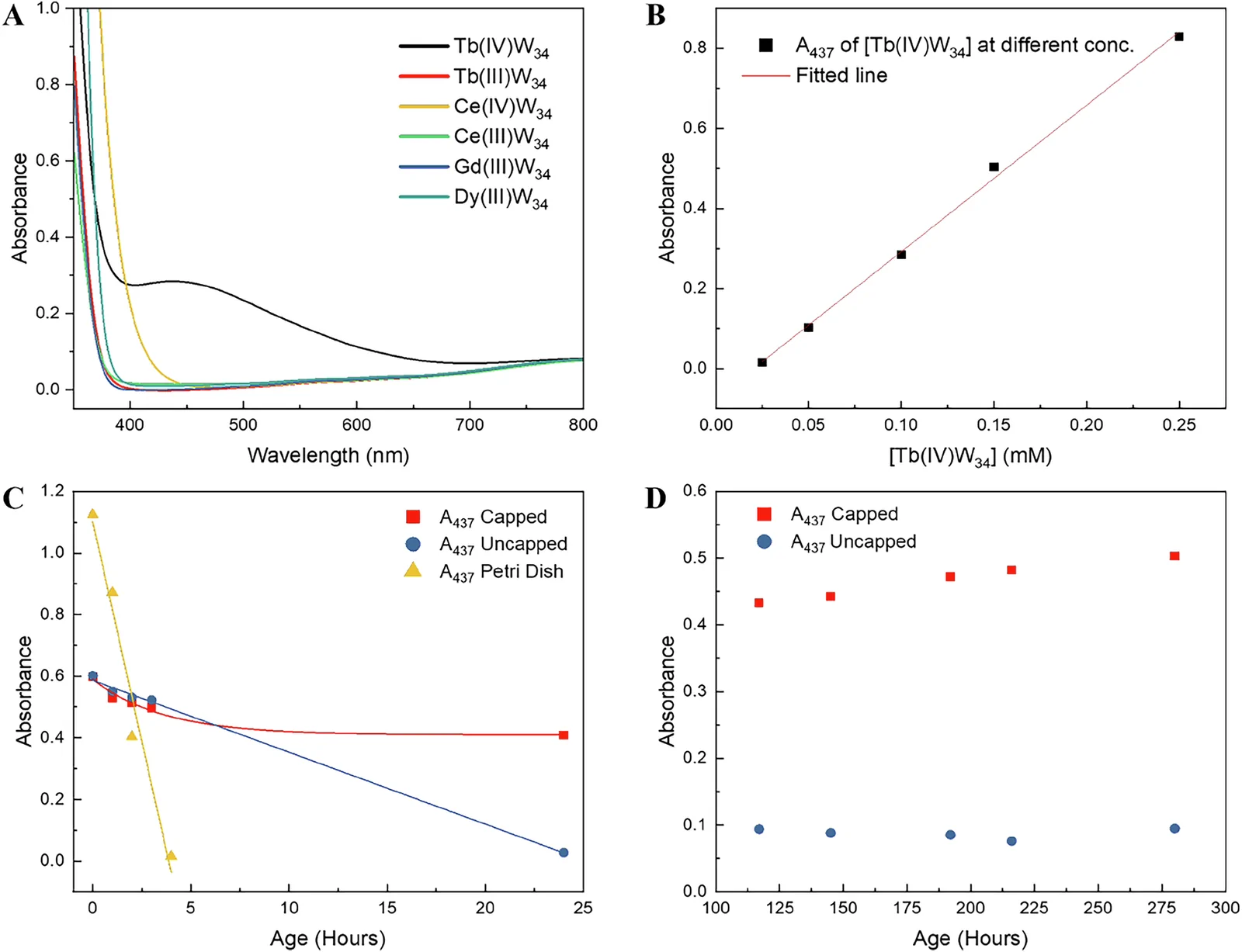
UV–Vis-NIR spectroscopy was used to probe polyoxometalate (POM) properties in solution and to probe Tb oxidation states within POM complexes. **A** UV–Vis–NIR absorbance spectra of Tb(IV)W_34_, Tb(III)W_34_, Ce(IV)W_34_, Ce(III)W_34_, Gd(III)W_34_, and Dy(III)W_34_. The concentration of each complex was 0.25 mM and spectra were collected in 1 cm path length cuvettes (I = 0.1 M, *T* = 25 °C). **B** Figure of absorbance at 437 nm (A_437_) versus concentration of Tb(IV)W_34_ and a linear fit for the data. The fitted line equation is y = (3.66 ± 0.10)x – (0.074 ± 0.015) is mapped to the Beer’s Lambert law (A = ε[Tb(IV)W_34_]l; l = 1 cm) which results in an ε value of 3660 M^−1^ cm^−1^. **C** Figure of A_437_ versus age over a period of 24 h showing the linear and exponential reductions of A_437_ for the uncapped and petri dish samples, respectively, until the A_437_ signals are gone, while the capped sample A_437_ persists after 24 h. **D** Figure of A_437_ versus age over a period of 300 h showing the A_437_ feature persists over the entire observation period.

The UV–Vis-NIR spectrum of Tb(IV)W_34_ shows a broad absorption band centered at 437 nm with a molar absorptivity (ε) of 3660 M^−1^ cm^−1^ that is absent from the spectrum of Tb(III)W_34_, Fig. 2. The high ε value and broadness of the peak suggests this feature originates from a ligand to metal charge transfer band, and this spectral feature is consistent with UV–Vis-NIR results from Saprykin et al. and Fedoseev and colleagues for Tb(IV)W_34_^39,40^. The La Pierre and Mazzanti groups have reported that their Tb(IV) complexes exhibit LMCT spectral bands that are absent from spectra of Tb(III) analogues, and here we also confirmed the observed spectral feature at 437 nm was specific to Tb(IV)W₃₄ and a result of oxygen to terbium ligand-to-metal charge transfer using time-dependent density functional theory calculations (Fig. S5, Supplementary Information). The absorption maxima of Tb(IV)W_34_ (λ_max_ = 437 nm) is redshifted when compared to [Tb(IV)(OSi(OtBu)₃)₄] (Tb(IV)OSi₄) (λ_max_ = 371 nm) indicating a smaller energy difference between the electronic ground state and excited state, and this change is expected as differences in crystal field splitting affect electronic energy states due to changes in the coordination symmetry between Tb(IV)W_34_ (*D*_4*d*_) and Tb(IV)OSi_4_ (*T*_*d*_)^18,19^. To confirm the emergence of the charge transfer band in the Tb(IV)W_34_ spectrum is due to oxidation of Tb(III) to Tb(IV) and not from unaccounted for interactions between K_2_S_2_O_8_ and Ln(III)W_34_ complexes, we subjected Ce(III)W_34_, Gd(III)W_34_, and Dy(III)W_34_ to the same synthesis regime as Tb(IV)W_34_ and compared their UV–Vis-NIR spectra, Fig. 2A. The spectra of Gd(III)W_34_ and Dy(III)W_34_ are identical before and after addition of K_2_S_2_O_8,_ which eliminates interactions between persulfate and the Ln(III)W_34_ cluster itself as the source of the charge transfer band at 437 nm, while Ce(III)W_34_ was oxidized to Ce(IV)W_34_ after addition of the K_2_S_2_O_8_, as indicated by a change in color from brown to light yellow and a redshift in the onset of the Ln(III)W_34_ POM absorption band, Fig. S6 (Supplementary Information). Moreover, the Tb(IV) metal center in Tb(IV)W_34_ was found to be stable in a closed aqueous system as demonstrated by longitudinal UV–Vis-NIR measurements wherein the molar absorptivity of the LMCT band (A_437_) was monitored, Fig. 2C, D. Upon transferring the Tb(IV)W_34_ solution from a cuvette to container with greater surface area such as a plastic petri dish, while maintaining the same volume, the A_437_ decreases exponentially, Fig. 2C. Extending measurements on Tb(IV)W_34_, kept under closed aqueous conditions, revealed the sustained presence of the 437 nm charge transfer peak over twelve days, Fig. 2D.

**Fig. 3:**
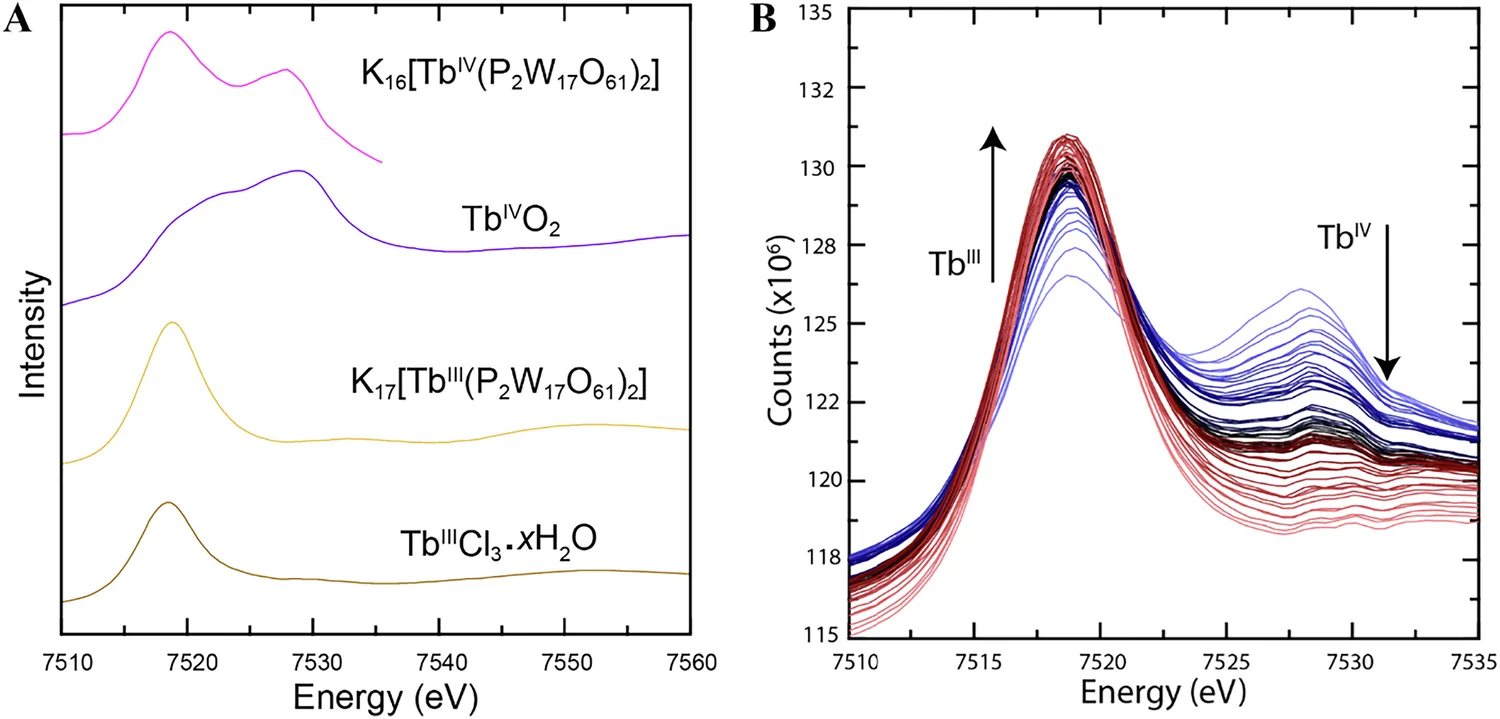
X-ray absorption spectroscopy measurements further probed Tb oxidation states within polyoxometalate complexes. **A** The low temperature (10 K) Tb *L*_3_-edge XANES of Tb(IV)W_34_, Tb(III)W_34_, Tb(IV)O_2_, and Tb(III)Cl_3_·*x*H_2_O. Data for Tb(IV)W_34_ were collected from freshly prepared aqueous solutions that were flash frozen prior to data acquisition. Spectra obtained for Tb(III)W_34_, Tb(IV)O_2_, and Tb(III)Cl_3_·*x*H_2_O were obtained on solid state samples. Spectra for Tb(III)W_34_, Tb(IV)O_2_, and Tb(III)Cl_3_·*x*H_2_O were calibrated in energy, background subtracted, and normalized. Data for Tb(IV)W_34_ were only calibrated in energy. Background subtraction and normalization were not conducted on this data. **B** Low temperature (10 K) Tb *L*_3_-edge XANES repeatedly obtained every 2 min and 10 s for Tb(IV)W_34_. These spectra were obtained from an aqueous solution that contained freshly prepared Tb(IV)W_34_. The sample was flash frozen prior to data acquisition.

Analysis of Tb(IV)W_34_ using Tb *L*_3_-edge X-ray absorption near edge spectroscopy confirmed that this complex contained Tb in the +4 oxidation state^19,44–48^. Evidence for this conclusion comes from the low temperature (10 K) Tb *L*_3_-edge XANES data for Tb(IV)W_34_, which was compared to Tb(III)W_34_ and two Tb oxidation state standards, namely Tb(IV)O_2_ (synthesized in-house) and Tb(III)Cl_3_·*x*H_2_O (obtained commercially), Fig. 3. The XANES spectrum obtained for Tb(IV)W_34_ displayed the double peak absorption feature characteristic of Tb in the +4 oxidation state (Fig. 3A). The energy of the inflection point for the rising edge was 7516.8(1) eV and the peak maxima energies for the two main absorption peaks were 7518.7(1) and 7527.9(1) eV. This spectrum was similar to that from the Tb(IV) standard (TbO_2_), which has been reported previously^49,50^, and featured inflection point and peak maxima energies of 7517.3(1), 7518.8(1), and 7528.8(1) eV, respectively. In contrast, Tb *L*_3_-edge XANES data for Tb(III)W_34_ and Tb(III)Cl_3_·*x*H_2_O oxidation state standards each contained a single absorption peak, typical for Tb(III) species^44,51–54^. The energies for the inflection point and peak maxima for the Tb(III) standards were slightly lower in energy than those observed for the Tb(IV) compounds, Table S3 (Supplementary Information).

It was not possible to quantitatively analyze peak intensities for the double peak absorption feature associated with Tb(IV)W_34_ because this complex decomposed during XANES measurements. To characterize the extent of decomposition, Tb *L*_3_-edge XANES measurements were rapidly and repeatedly obtained (every 2 min and 10 s) for seven hours over the absorption edge (c.a. 7510 to 7535 eV). Collectively, these spectra showed that both the low-energy [7518.7(1) eV] and high-energy [7527.9(1) eV] Tb(IV) absorption peaks decreased in intensity and disappeared during data acquisition. Concomitantly, a lower energy Tb(III) single absorption peak increased in intensity and replaced the Tb(IV) peaks, Fig. 3B. The Tb(III) decomposition product inflection point and peak maximum energies were 7516.2(2) and 7518.5(1) eV, respectively. The rate of the Tb(IV) to Tb(III) transformation was also investigated via monitoring of how the total counts from the Tb(IV) high-energy peak maximum [7527.9(1) eV] and the Tb(III) decomposition product [7518.5(1) eV] varied as a function of time upon exposure to X-rays. In less than one hour, evidence for Tb(IV) in Tb(IV)W_34_ had completely disappeared Fig. S7 (Supplementary Information). Despite its fleeting nature, these XANES data unambiguously confirmed the presence of Tb(IV) within Tb(IV)W_34_ under aqueous conditions.

**Fig. 4:**
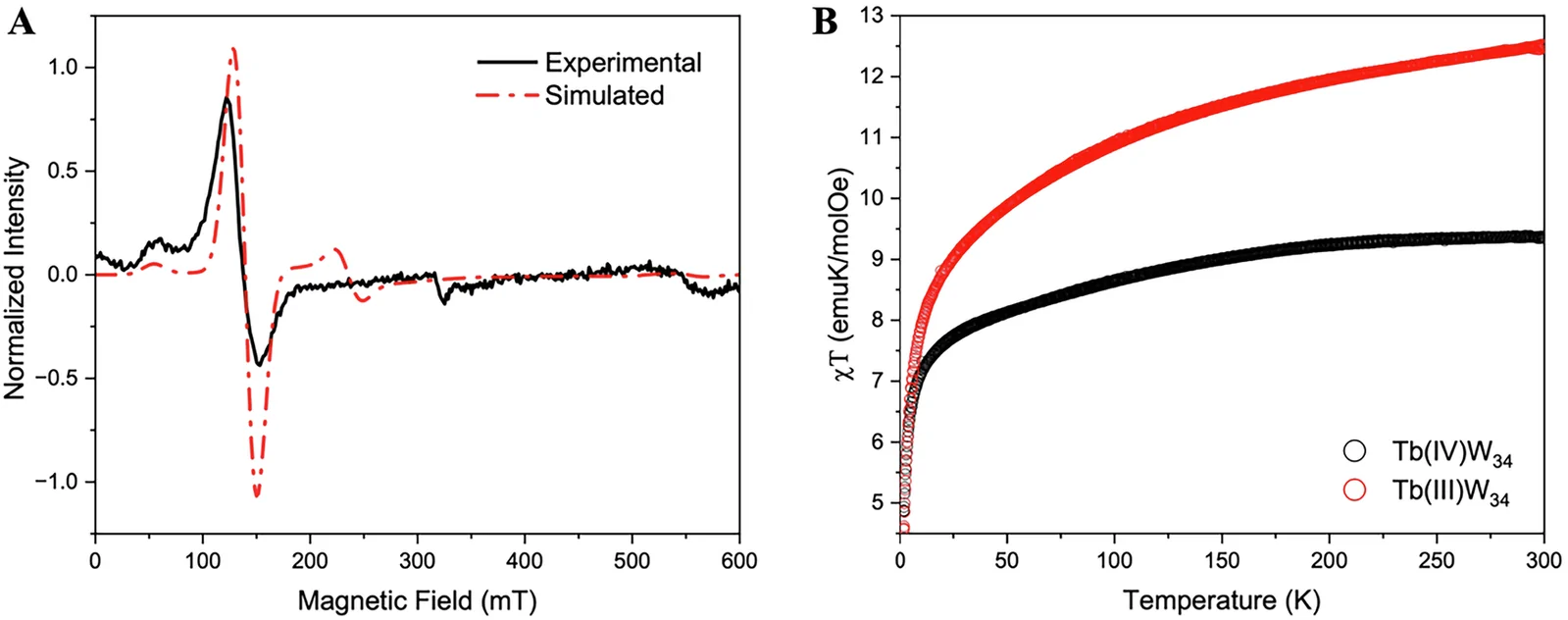
Electron paramagnetic resonance and SQUID magnetometry measurements provided insights into the electronic structure of Tb-polyoxometalate complexes. **A** Continuous wave X-band EPR spectrum of Tb(IV)W_34_ (black trace) and the simulated spectrum (red trace) obtained using EasySpin. **B** Variable temperature molar magnetic susceptibility (χT) versus temperature (K) plots for Tb(IV)W_34_ and Tb(III)W_34_ collected under a dc field of 1000 Oe.

Additional confirmation of the Tb oxidation state in Tb(IV)W_34_ was provided by continuous wave (CW) X-band EPR spectroscopy. EPR is an ideal technique to detect the presence of Tb(IV) molecular species because the 4*f*^7^ electron configuration of Tb(IV) results in readily detectable spectra at commercially available X-band frequencies, in contrast to 4*f*^8^ Tb(III) complexes that are typically EPR silent. In this study, X-band EPR data were collected on Tb(III)W_34_ and Tb(IV)W_34_ at 5 K, Fig. 4A. The Tb(III) complex was EPR silent as anticipated, while the Tb(IV)W_34_ spectrum includes two distinct low field transitions at around 50 and 150 mT that are in agreement with the spectra for tetrahedrally coordinated Tb(IV) systems from La Pierre and Mazzanti^18,19^. Only one of the Tb(IV)NP_4_ complexes contains a third transition below 50 mT that may arise from the higher rhombicity provided by the NP_4_ ligand^19,31^. In contrast, Tb(IV) ions trapped in glasses, silicates, or germanate lattices generally contain features above 150 mT at X-band frequencies^55–57^. We simulated our spectrum using the EasySpin toolbox within MatLab^58,59^, and the electronic states of the Tb(IV) species can be described using the following effective spin Hamiltonian (Eq. 1).

1 $$H={{{\bf{g}}}}{{{{\rm{\mu }}}}}_{{{{\rm{B}}}}}{{{\bf{B}}}}{{\cdot }}\hat{{{{\bf{S}}}}}+D\left[{S}_{z}^{2}-\left(\frac{1}{3}\right)S\left(S+1\right)\right]+E({S}_{x}^{2}-{S}_{y}^{2})$$

The first term describes the Zeeman interaction caused by the applied magnetic field and it is determined based on ${{{\bf{g}}}}$, which describes interactions between the applied magnetic field and an electron, ${\mu }_{{{{\rm{B}}}}}$, the Bohr magneton, ${{{\bf{B}}}}$, the applied magnetic field, and $\hat{{{{\bf{S}}}}}$, the spin operator. We did not include a total orbital angular momentum term, $\hat{{{{\bf{J}}}}}$, due to 4*f*^7^ systems having L = 0, which leads to a pure spin system. As a result, we anticipated that the *g*-factor should be isotropic with a magnitude of around 2. The second and third terms describe the anisotropic and rhombic zero-field splitting (ZFS) terms, where *D* and *E* represent the magnitude of the anisotropic and rhombic ZFS parameters, respectively. We included only *D* and *E* terms in the effective spin Hamiltonian as X-band spectra lack the resolution to resolve the energy level splitting of higher order crystal field parameters. The experimental spectrum of Tb(IV)W_34_ can only be simulated by employing an S = 7/2 system, clearly indicating the signal originates from tetravalent terbium (^8^S_7/2_), Fig. 4A. The values obtained from the simulation of the Tb(IV)W_34_ spectrum were found to be *g*_iso_ = 2.01, *D* = 0.634 cm^−1^ (19000 MHz) and E = 0.0667 cm^−1^ (2000 MHz). The assignment of the *g*_iso_ is based on the results from the La Pierre group for their Tb(IV)NP_4_ complex^31^, and the *D* and *E* values reported for the Tb(IV)NP_4_ complex are 0.140 cm^−1^ (4197 MHz) and 0.0250 cm^−1^ (749.5 MHz), respectively^31^. The difference in *D* values, the value for Tb(IV)W_34_ is more than four times higher than Tb(IV)NP_4_, can be rationalized from a coordination symmetry perspective. The *D*_4*d*_ symmetry provided by the W_17_ ligands is pseudo axial, while the *T*_*d*_ symmetry provided by the NP ligands is more isotropic, which would result in greater anisotropic crystal field splitting. The spectrum of Tb(IV)W_34_ was also compared to the X-band spectrum for the isoelectronic Gd(III)W_34_ and this revealed stark differences (Fig. S8 and Table S4, Supplementary Information) as the major feature in the spectrum of Tb(IV)W_34_ is centered at 139 mT, while the primary spectral band for Gd(III)W_34_ is centered at 337 mT. Based on simulation of the Gd(III)W_34_ spectrum, the *D* and *E* values for Gd(III)W_34_ are 0.0587 cm^−1^ (1760 MHz) and 0.00490 cm^−1^ (147 MHz), respectively. The much smaller *D* and *E* values for Gd(III)W_34_ compared to Tb(IV)W_34_ are consistent with previous findings from La Pierre and colleagues^31^, who found that significantly larger ZFS values for Tb(IV) are the result of an increase in spin orbit coupling going from Gd(III) to Tb(IV), and our results confirm this trend applies for W_34_ complexes.

Beyond XAS and CW X-Band EPR measurements, we have also conducted variable temperature direct current magnetometry measurements on Tb(IV)W_34_ and Tb(III)W_34_ complexes to further investigate the electronic structure of each of these species. Figure 4B shows the χT versus T (K) spectra for both Tb complexes and the curve for Tb(IV)W_34_ (black trace) lies well below that of Tb(III)W_34_ (red trace). Plotting 1/χ versus temperature (Fig. S9, Supplementary Information) allows us to extract effective magnetic moments (μ_eff_) for comparison with calculated theoretical (μ_calc_) values. The μ_eff_ values for Tb(IV)W_34_ and Tb(III)W_34_ are 8.76 μ_B_ and 10.15 μ_B_, respectively, while the μ_calc_ values are 7.94 μ_B_ (Tb(IV), 4*f*^*7*^, ^8^S_7/2_) and 9.72 μ_B_ (Tb(III), 4*f*^8^, ^7^F_6_). Effective magnetic moment values for both complexes are broadly consistent with expectations for Tb(IV) and Tb(III) complexes^19^, with the possibility of a small amount of reduction of Tb(IV)W_34_ occurring during the sample loading process ahead of measurement, and the field dependent behavior noted for Tb(IV)W_34_ (Fig. 4B) likely arises from increased Zeeman splitting at higher fields.

Finally, the electronic structure of Tb(III)W_34_ and Tb(IV)W_34_ complexes were computationally assessed using density functional theory. The calculated density of states for both Tb POM complexes are highlighted in Fig. S10 (Supplementary Information), which display traditional polyoxometalate electronic structure with an occupied oxide and empty metal band that are dominated by oxygen 2*p* and tungsten 5 *d* contributions, respectively. The occupied terbium *f*-orbitals reside at lower energies, consistent with previous computational studies on Tb(IV) coordination complexes^19^. Upon oxidation, the Mulliken spin density of terbium increases from 6.02 to 7.63, indicative of a transition from a 4*f*^8^ to 4*f*^7^ electron configuration. No significant spin density was observed in the polyoxometalate ligands, confirming that the oxidation takes place at the lanthanide center, Fig. S10 (Supplementary Information). The Tb(IV/III) reduction potential was calculated to be +1.60 V, with the absolute potential of the hydrogen electrode taken to be 4.44 V^60,61^.

In this investigation, Tb(IV)W_34_ and Tb(III)W_34_ complexes have been synthesized and characterized via multiple spectroscopic techniques to prove that Tb(IV) can be isolated and characterized in aqueous media in the presence of persulfate. The crystal structure of Tb(IV)W_34_ shows a d_Tb-O_ reduction of 0.098 Å compared to Tb(III)W_34_, while the UV–Vis-NIR spectrum of Tb(IV)W_34_ shows a broadband feature centered at 437 nm that we have identified experimentally and computationally as a Tb(IV) ligand to metal charge transfer band, consistent with the findings from Fedoseev and colleagues^40^. The LMCT band persists for over twelve days in water in a capped cuvette demonstrating aqueous stability of tetravalent terbium when desolvation effects are minimized. This LMCT band is absent from the UV–Vis-NIR spectra of all the other complexes investigated in this study, including Gd(III)W_34_, indicating the electronic manifolds of Tb(IV)W_34_ are distinct despite having the same 4*f*^7^ electronic configuration as the Gd(III) congener. Contrasts between the isoelectronic Tb(IV)W_34_ and Gd(III)W_34_ also manifest as markedly different ZFS parameters (*D* and *E*), which were extracted from fittings of continuous wave X-band EPR spectra for both complexes. The X-band EPR spectra of Tb(IV)W_34_ can only be simulated by assuming S = 7/2, a characteristic feature of a 4*f*^*7*^ electron configuration that is indicative of terbium in the +4 oxidation state. The ZFS values extracted for Tb(IV)W_34_ are also an order of magnitude higher than those extracted for Gd(III)W_34_, indicating an increase in crystal field interactions for Tb(IV)W_34_, which results from larger spin-orbit coupling that is known for tetravalent lanthanide cations. XANES measurements definitively confirmed the oxidation state assignment for Tb(IV)W_34_ under aqueous conditions, although the complex proved unstable under the X-ray beam. Zero field cooled variable temperature SQUID magnetometry measurements further indicated the tetravalent character of Tb(IV)W_34_, with the μ_eff_ value for the complex broadly consistent with expectations for Tb(IV) species. Density functional theory calculations were used to computationally support the oxidation state assignment within Tb(IV)W_34_ and included electronic density of states calculations that showed an increase in the Mulliken spin density of terbium upon oxidation. This increase is associated with a transition from a 4*f*^8^ to 4*f*^7^ electron configuration and indicates that the oxidation of the Tb(III) to Tb(IV) does not involve the lacunary Wells Dawson POM ligands. The redox potential of the Tb(IV)/Tb(III) pair in Tb(IV)W_34_ has also been calculated to be +1.60 V, which is a 1.5 V cathodic shift compared to the standard Tb(IV)/Tb(III) redox potential (3.1 V vs NHE)^40^. Overall, this study demonstrates the ability to isolate and characterize Tb(IV) under aqueous conditions and serves as proof of the ability of POM ligands to stabilize unusual oxidation states within the 4*f*-elements. Further development of this system could lead to a straightforward oxidation-based separation of Tb from neighboring rare-earth elements, new approaches for generating redox-switchable magnetic materials, as well as providing pivotal fundamental insights into the relationship between metal-ion oxidation state and the electronic manifolds of *f*-elements.

## Methods

### Materials

Lanthanide starting materials other than TbO_2_ are commercially available and were used as received. These include terbium(III) nitrate hexahydrate (Tb(NO_3_)_3_•6H_2_O) (Strem Chemicals, Inc., 99 + %), ceric(IV) ammonium nitrate ((NH_4_)_2_Ce(NO_3_)_6_) (Thermo Scientific, 98%), cerium(III) chloride heptahydrate (CeCl_3_•7H_2_O) (BeanTown Chemical, 99%), gadolinium(III) chloride hexahydrate (GdCl_3_•6H_2_O) (BeanTown Chemical, ≥99.9%), dysprosium(III) nitrate pentahydrate (Dy(NO_3_)_3_•5H_2_O) (BeanTown Chemical, 99.9%), terbium(III) oxide (Tb_2_O_3_) (Sigma Aldrich, 99.99%), and terbium (III/IV) oxide (Tb_4_O_7_) (Thermo Scientific, 99.9%). Ammonium persulfate ((NH_4_)_2_S_2_O_8_, 98 + %) and potassium persulfate (K_2_S_2_O_8_, 99%) were obtained from Fisher Scientific. K_10_P_2_W_17_O_61_•20H_2_O was synthesized following the procedure from Contant et al.^62^ and K_6_P_2_W_18_O_62_•nH_2_O was synthesized using the procedure from Graham and Finke^63^.

#### Synthesis of K_16/17_Ln(III/IV)(P_2_W_17_O_61_)_2_•*x*H_2_O (LnW_34_) where Ln = Ce(III/IV), Gd(III), Tb(III), Dy(III)

The synthesis protocol used to prepare LnW_34_ complexes is based on the procedure first outlined by Peacock and Weakley^64^. 0.25 grams of K_10_P_2_W_17_O_61_•20H_2_O (0.05 mmol) was dissolved in 9.75 mL Milli-Q H_2_O with heating (90 °C) and stirring (pH = ~6.5). Subsequently, 250 μL of 0.1 M Ln(NO_3_)• *x*H_2_O/LnCl_3_•*x*H_2_O/(NH_4_)_2_Ce(NO_3_)_6_ (0.025 mmol) solution was added in 50 μL portions into the heated (90 °C) polyoxotungstate solutions, and pH values stayed consistent at approximately 6.5. After stirring for ten minutes, the solutions were centrifuged for five minutes at 6100 x *g* (7830 rpm), and the supernatant solutions were collected in plastic Petri dishes. The Gd(III), Tb(III), and Dy(III) solutions were faint yellow in color, while the Ce(III) and Ce(IV) supernatants were brown and yellow, respectively. LnW_34_ crystals were grown from the supernatant solutions in approximately seven days by slow evaporation on the benchtop and yields (by weight) for all systems that used this procedure were approximately 70%.

#### Synthesis of K_16_[Tb(IV)(P_2_W_17_O_61_)_2_]*•x*H_2_O

To prepare Tb(IV)W_34_, 100 molar eq of K_2_S_2_O_8_ was added into a solution containing 1 eq of Tb(III)W_34_ solution, prepared using the procedure outlined above, with stirring at 80 °C, which lowers the reaction solution pH to ~2.5. The solution started to change color to brown after ten minutes, indicative of Tb(III) oxidation to Tb(IV), and the color was saturated after twenty minutes of heating. The final color of the solution was dark brown. The reaction solution was then cooled down to room temperature, transferred to an airtight container, and stored in the fridge (5 °C). After one day, colorless and brown crystals were observed. The colorless crystals were unreacted K_2_S_2_O_8_, while the brown crystals were Tb(IV)W_34_ (54% yield (semi-quantitative)). The brown crystals were stored in the fridge (5 °C) in a capped under air container filled with the mother liquor to slow down the auto-reduction process back to Tb(III)W_34_. Synthesis of Tb(IV)W_34_ was also pursued using Schlenk techniques and in an argon glovebox, and storage of the POM crystals in sample mother liquor was still required to limit auto-reduction to the Tb(III) analogue. Worthy of additional comment, obtaining a yield value for Tb(IV)W_34_ is challenging due to co-crystallization with K_2_S_2_O_8_. Gravimetric analysis and scanning electron microscopy-energy dispersive spectroscopy (SEM-EDS) (Table S6 and Fig. S11, Supplementary Information) were used to obtain the semi-quantitative yield value provided above. Details regarding this yield calculation can be found in the Supplementary Information.

#### Synthesis of TbO_2_

0.75 g of dark brown Tb_4_O_7_ was transferred into a glass test tube. 1 mL of concentrated acetic acid (17.4 M) and 1 mL of concentrated HCl (12.1 M) were added into the tube containing Tb_4_O_7_. The suspension was then boiled at a temperature of 105 °C for one hour. After boiling, the solid brown powder turns slightly reddish, and the suspension was transferred into a 15 mL centrifuge tube with a 50/50 mixture of H_2_O and acetone and centrifuged for five minutes at 6100 × *g* (7830 rpm). The supernatant solution was removed, and the reddish brown solid was washed with H_2_O three times to remove any Tb(III)Cl_3_ and Tb(III)(CH_3_COOH)_3_ byproducts. The final suspension was then transferred to a glass petri dish to dry in the fume hood. Once dry, the product was harvested and characterized via powder X-ray diffraction to ensure phase purity, Fig. S1 (Supplementary Information).

### Characterization

#### X-Ray structure determination

Single crystal diffraction data for Tb(IV)W_34_, Tb(III)W_34_, Ce(IV)W_34_, Ce(III)W_34_, Gd(III)W_34_, Dy(III)W_34_, and the Ce(IV) polymorph (Ce(IV)W_34__version II) were collected at 100(2) K on a Bruker D8 Venture Duo diffractometer using a Mo X-ray source, K_α1_ = 0.71073 Å. Crystals of Tb(IV)W_34_ were sensitive to desolvation and, after a period of approximately ten minutes, lose their brown color indicating the reduction of Tb(IV) back to Tb(III). Tb(IV)W_34_ crystals also quickly dissolve back into the mother liquor, in under five minutes, at room temperature. To address these challenges, mounting of the crystals was done on a makeshift stage with an ice pack to keep the temperature low and the crystal of Tb(IV)W_34_ was mounted in the liquid N_2_ cold stream in under ten minutes. Absorption corrections were applied for all datasets using the SADABS multi-scan method within the APEX5 software package^65^. Structures were solved via intrinsic phasing using SHELXT and refined with SHELXL contained within Olex2 1.5^66–68^. All atoms in the LnW_34_ POM units were refined anisotropically. Due to disorder within the lattices of the POM complexes, some of the counterions and lattice water oxygen atoms were refined with less than full chemical occupancy. ISOR (0.01–0.001) restraints were used on lattice potassium and oxygen atoms to provide reasonable thermal ellipsoids, when necessary, in all complexes, while EADP constraints were also applied to partially occupied potassium atoms in Gd(III)W_34_ (K17A, K17B, K9, and K10) and Tb(III)W_34_ (K18A and K18B) structures. Ce(III)W_34_ was solved as a two-component twin and RIGU (0.01–0.001) and SIMU (0.01–0.001) restraints were required to model select cluster and lattice oxygen atoms, along with applying the appropriate twin law. Twinning in the Ce(III)W_34_ data also led to greater disorder in the lattice, and limited the extent to which lattice water molecules could be modeled, which is the origin of the significantly smaller number of lattice water molecules included in the complex formula compared to other LnW_34_ species. Bond valence calculations were conducted with PLATON version 230318^69^ in Olex2 1.5^66^, and all publication images were generated using CrystalMaker^70^. Crystallographic parameters for Tb(IV)W_34_, Tb(III)W_34_, Ce(IV)W_34_, Ce(III)W_34_, Gd(III)W_34_, Dy(III)W_34_, and the Ce(IV) polymorph (Ce(IV)W_34__version II) can be found in Table S1 (Supplementary Information).

#### Tb *L*_3_-edge XAS measurements

All Tb *L*_3_-edge XAS data were collected at the Stanford Synchrotron Research Laboratory (SSRL), beamline 4–3, under dedicated operating conditions (3.0 GeV, 500 mA) at a temperature of 10 K. This beamline is equipped with a 20-pole, 2.0 Tesla wiggler and utilizes a liquid N_2_-cooled double-crystal Si[111] monochromator (FWHM beam spot size = 3 × 16 mm). The crystals were de-tuned by 30% from the rocking curve maximum. Two mirrors were utilized, the first mirror for vertical collimation and the second mirror for vertical and horizontal focus. The experimental set up at beamline 4-3 is best described as having three ion chambers downstream from the beam pipe. The beam pipe was capped with a beryllium window and ion chambers were sealed with polypropylene windows (4 μm). A potential of 2000 V was applied to the ion chambers, through which helium gas was continually flowed. One chamber (30 cm long) was positioned before a cryostat to monitor the incident radiation (*I*_*0*_). The second chamber (30 cm long) was positioned after the cryostat, such that sample transmission (*I*_*1*_) could be evaluated against *I*_*0*_, while a third chamber (*I*_*2*_, 30 cm long) was positioned downstream from *I*_*1*_ so that the XANES of calibration samples could be measured in situ during the XAS experiments and compared to *I*_*1*_. Samples were loaded into the cryostat, which was equipped with two sets, inner and outer, of Kapton windows (2 mil). There was additionally a ~ 12 μm thick radiation shield. Overall, the beam traversed approximately 112 μm of Kapton to get to the sample and the subsequent sample X-ray fluorescence traversed through the same amount of Kapton on its way to the detector. All of these components were Al-coated. Moreover, the cryostat was separated from the beam pipe by a small air gap (~2.5 cm). Sample fluorescence was monitored using a partially depleted series (PIPS^®^) charged particle detector with a 5000 mm^2^ active area (PD5000-75-500AM) that was positioned at a 90° angle from the incident beam and suspended as close as possible to the cryostat, which minimized the air gap between the cryostat and the detector. Hence, sample fluorescence was measured against the incident radiation (*I*_*0*_). Spectra were collected with acquisition times of 1 s per data point for K_17_[Tb(III)(P_2_W_17_O_61_)_2_]·*x*H_2_O, Tb^IV^O_2_, and TbCl_3_·*x*H_2_O and 0.5 s per data point for K_16_[Tb(IV)(P_2_W_17_O_61_)_2_]·*x*H_2_O. Radiation damage was characterized by rapidly and repeatedly scanning the analyte absorption edge (10 s/scan) prior to conducting the full spectrum measurement. Spectra from K_17_[Tb(III)(P_2_W_17_O_61_)_2_]·*x*H_2_O, Tb(IV)O_2_, and TbCl_3_·*x*H_2_O were obtained in triplicate. Spectra obtained from K_16_[Tb(IV)(P_2_W_17_O_61_)_2_]·*x*H_2_O were collected only over the absorption edge (7507 to 7540 eV) because of the rapid sample decomposition that occurred upon exposure of the sample to the X-ray incident radiation. Energy calibrations were conducted in situ using Tb(IV)O_2_ samples in transmission mode.

#### Sample preparation for Tb *L*_3_-edge XAS measurements

Solution phase Tb *L*_3_-edge XANES samples were prepared that contained approximately 10 mg/mL of Tb dissolved in aqueous solutions. Solution phase measurements at these Tb concentrations strike an acceptable balance that provides sufficient sample for high signal-to-noise spectra while minimizing contributions from self-absorption. The K_16_[Tb(IV)(P_2_W_17_O_61_)_2_]·*x*H_2_O solution was prepared in a fume hood by boiling a mixture of K_17_[Tb(III)(P_2_W_17_O_61_)_2_]·*x*H_2_O (80 mg, 8.1 µmol) with (NH_4_)_2_S_2_O_8_ (200 mg, 0.88 µmol) in H_2_O (1 mL) in a glass vial (3 mL) where the solution pH was approximately 2.6. An aliquot of the resulting orange-brown solution (70 μL) was then transferred into a sample holder that had been 3D-printed from VeroClear (Stratasys). This holder was best described as an open box whose inner dimensions were 18 mm (height) × 2 mm (width) × 2 mm (depth) with a total volume of approximately 0.07 mL. The open side of the sample holder was sealed with Kapton tape (0.5 mil; 12.7 μm). Solutions were pipetted into the holder through a hole that had been bored through one end of the box. This injection hole was subsequently sealed with Kapton tape and the sample was placed in the holder attached to a rod and flash frozen in a bath of liquid nitrogen. The frozen sample was transferred to the end-station hutch and rapidly inserted into the cryostat against a strong flow of helium. The cryostat was evacuated and refilled with He (3×), cooled to 10 K, and refilled with helium. The solid-state Tb *L*_3_-edge XANES samples were prepared by grinding the samples with BN in polycarbonate canister that contained a Teflon bead using a Wig-L-Bug for 2 min. The K_17_[Tb(III)(P_2_W_17_O_61_)_2_]·*x*H_2_O (10 mg, 1 μmol) sample was ground with 27.8 mg of BN, the Tb(IV)O_2_ sample (5.8 mg, 16 μmol) was ground with 54.5 mg of BN, and the TbCl_3_·*x*H_2_O (5.6 mg, 15 μmol) sample was ground with 27.4 mg of BN. Prior to making these measurements, we determined that these quantities of terbium in solid-state samples provided an acceptable balance that provides sufficient sample for high signal-to-noise spectra while minimizing contributions from self-absorption. Subsequently, a thick film of each ground solid sample was dispersed on a piece of Kapton tape (0.5 mil; 12.7 μm) using a paint brush. Samples were sealed with a layer of Kapton tape (0.5 mil; 12.7 μm) and the assembly was fixed to an aluminum sample holder that had a 1 mm × 5 mm × 20 mm well.

#### Tb *L*_3_-edge XAS data analysis

Data manipulations and analyses were conducted using a combination of the Athena and IGOR software packages. Energies were calibrated to the first inflection point from Tb(IV)O_2_ (7517.25 eV). Data from K_17_[Tb(III)(P_2_W_17_O_61_)_2_]·*x*H_2_O, Tb(IV)O_2_, and TbCl_3_·*x*H_2_O were analyzed by fitting a line to the pre-edge region that was subsequently subtracted from the experimental data to eliminate the background of the spectrum. Data were then normalized by fitting a first-order polynomial to the post-edge region of the spectrum and setting the edge jump to an intensity of 1.0. This procedure gave spectra normalized to a single Tb atom. Data from K_16_[Tb(IV)(P_2_W_17_O_61_)_2_]·*x*H_2_O were also calibrated to the first inflection point from Tb(IV)O_2_. However, this data was not background subtracted nor was it normalized. Important metrics associated with all spectra–inflection points and peak maxima–were determined graphically. The first inflection point of the data were determined to be the point at which the second derivative of the data equaled zero. The peak maxima were determined by identifying the points at which the first derivatives of each spectra equaled zero.

#### UV–Vis-NIR spectroscopy

UV–Vis-NIR spectra were collected at room temperature on Agilent Cary 5000 UV–Vis–NIR spectrometers at the University of Iowa and Lawrence Berkeley National Laboratory with tungsten halogen visible and deuterium arc UV light sources. Measurements were collected with quartz cuvettes if not specified otherwise. Data were collected between 200nm and 800 nm at a scan rate of 10 nm/s. Below 350 nm, the detector was saturated due to absorption of the polyoxometalate ligands, Fig. S2 (Supplementary Information); hence, data below 350 nm were omitted. The UV–Vis–NIR spectra of Tb(IV)W_34_, Tb(III)W_34_, Ce(IV)W_34_, Ce(III)W_34_, Gd(III)W_34_, and Dy(III)W_34_ were collected from freshly prepared 0.25 mM solutions of each POM. The longitudinal stability study was conducted on two samples of Tb(IV)W_34_, each at a concentration of 0.25 mM, with one of the samples capped and the other one left uncapped with data collected regularly over twelve days. Sample pH was monitored during the duration of measurements and stayed consistent for both capped and uncapped solutions at approximately 2.6 (Table S5, Supplementary Information).

#### EPR spectroscopy

X-band electron paramagnetic resonance measurements on Tb(IV)W_34_ were performed on a Bruker Elexys E500 spectrometer with the center field set at 8100 G and a sweep width of 16000 G. The power was set to 20 mW with a phase modulation amplitude of 1 MHz. Spectra were collected at 5 K using an ESR 9000 cryostat with a Cold Edge Stinger that was adjusted with a Lakeshore temperature controller. Samples of Tb(IV)W_34_ analyzed using EPR spectroscopy were prepared by slow evaporation from a 5 mM solution, and crystals were separated from persulfate and subsequently harvested with a transfer pipette along with the mother liquor. This slurry was transferred to a 4 mm quartz EPR tube for X-band (9.48 GHz) measurements. Simulations of the EPR spectra of Tb(IV)W_34_ were done with EasySpin 6.0 within the MATLAB interface^58^, and models incorporated the zero-field splitting parameters, *D* and *E*, of Tb(IV)W_34_, while keeping *g* mostly isotropic at approximately 2. This fitting regime is consistent with expectations for a 4*f*^7^ system and is aligned with previously reported simulation parameters for Tb(IV) complexes^28^. The X-band EPR spectrum for Gd(III)W_34_ was collected on a Bruker Elexys E580 spectrometer equipped with a Bruker ER 4118X-MD5 X-band pulse resonator inside an Oxford CF935 flow cryostat. The temperature was maintained using liquid helium by an Oxford ITC503 temperature controller. Samples were pumped into a glovebox with a nitrogen atmosphere and loaded into 4 mm quartz capillary tubes and flame sealed. The reported spectrum was obtained with a 9.753 GHz field strength and a microwave power of 0.597 mW at 9 K with a phase modulation amplitude of 1 MHz.

#### SQUID magnetometry measurements

Powdered Tb(III)W_34_ was weighed and prepared in a vibrating sample magnetometer (VSM) powder sample container inserted into a brass holder and measured on a Quantum Design MPMS 3 SQUID magnetometer. The holder was packed tightly to prevent the microcrystalline samples from rearranging during measurements. For Tb(IV)W_34_, samples were weighed into the VSM powder sample container in the form of crystals (each was hand-picked and loaded) and subsequently turned into a powder within the sample holder. The instrument sequences used for the magnetometry measurements and details related to data processing for Tb(IV)W_34_ and Tb(III)W_34_ spectra are included in the Supplementary Information within the SQUID Magnetometry section.

#### Electronic structure calculation details

Density functional theory calculations were conducted using the ORCA 6.0.1 software package^71^. Geometries were optimized considering solvent effects via the conductor-like polarizable continuum model (CPCM) with water parameters^72^. The hybrid-exchange correlation functional PBE0^73^ was used in conjunction with the def2-TZVP basis set on metal centers and the def2-SVP basis set on all other atoms^74^. The nature of all stationary points was verified by analytic calculation of vibrational frequencies, which were used to calculate molecular partition functions. Subsequently, partition functions were used to calculate the 298.15 K thermal contributions to free energy employing the usual ideal-gas, rigid-rotor, and harmonic oscillator approximations. To correct for the breakdown of the harmonic oscillator approximation for low frequencies, entropic contributions to the free energies from frequencies below 100 cm^−1^ were computed using the quasi-RRHO approach of Grimme^75^. Standard state conditions were assumed for all species, and all calculations were performed without symmetry constraints. Single point calculations were performed assigning the def2-TZVP basis set on all atoms, marginally shifting the redox potential by 70 mV. Optimizations with larger basis sets for this system size were computationally prohibitive. All calculations are available in an ioChem-BD repository: (https://iochem-bd.bsc.es/browse/review-collection/100/479640/c0dc4e30af7af7f15db4e612).

## Supplementary information

Supplementary Information

Transparent Peer Review file

## Data Availability

The CIFs for Tb(IV)W_34_, Tb(III)W_34_, Ce(IV)W_34_, Ce(III)W_34_, Ce(IV)W_34__version II, Gd(III)W_34_, and Dy(III)W_34_ complexes have been deposited at the Cambridge Crystallographic Database Centre and may be obtained free of charge from http://www.ccdc.cam.ac.uk by citing reference numbers 2473065-2473071. The raw data for Figs. 2, 3, and 4 are made available as Source Data files and are available via FigShare^76^. The data that support the findings of this study are included in the main text and the Supplementary Information and can be obtained from the corresponding authors upon request.
