# Supplementary material for: Structural and spectroscopic characterization of a Tb(IV) polyoxometalate
Source: Nat Commun. 2026 Jul 16;17:8712. doi: 10.1038/s41467-026-75657-7 (PMC13490611; doi:10.1038/s41467-026-75657-7)
Supplement: Supplementary file 1 [file 41467_2026_75657_MOESM1_ESM.pdf]

Structural and Spectroscopic Characterization of a Tb(IV) Polyoxometalate

Primadi J. Subintoro,[1] Brett Lottes,[1,2] Felipe A. Pereiro,[2,3] Nolwenn Mahieu,[4] Alexander M. Brown,[4] S. Genevieve Duggan,[1] Monica S. Mullis,[2] Aldo. M. Jordan,[2] Cassandra Gates,[2] Samuel M. Greer,[2] Joshua J. Woods,[4] Rebecca J. Abergel,[4,5,6] Jenifer C. Shafer,[3] Daniel K. Unruh,[7] Pere Miro,[1]* Benjamin W. Stein,[2]* Jennifer N. Wacker,[4]* Stosh A. Kozimor,[2]* and Korey P. Carter[1]*

[1] Department of Chemistry, University of Iowa, Iowa City, IA 52242, United States

[2] Chemistry Division, Los Alamos National Laboratory, Los Alamos, NM 87545, United States

[3] Department of Chemistry and Geochemistry, Colorado School of Mines, Golden, Colorado 80401, United States

[4] Chemical Sciences Division, Lawrence Berkeley National Laboratory, Berkeley, CA 94720, United States

[5] Department of Nuclear Engineering, University of California, Berkeley, Berkeley, CA 94709 United States

[6] Department of Chemistry, University of California, Berkeley, Berkeley, CA 94720 United States

[7] Office of the Vice President for Research, University of Iowa, Iowa City, IA 52242, United States

* Corresponding author's e-mails: pere-miro@uiowa.edu, bstein@lanl.gov, jwacker@lbl.gov, stosh@lanl.gov, korey-carter@uiowa.edu

Supporting Information

Table of Contents

Figure S1 – PXRD spectrum of Tb(IV)O_2	**S2**
Figure S2 – UV-Vis-NIR spectrum of $K_{10}P_2W_{17}O_{61}$•20H_2O	**S3**
Table S1 – Crystallographic parameters for LnW_{34} crystal structures	**S4**
Table S2 – Average Tb-O bond distances and bond valence sums for Tb(IV)W_{34} and Tb(III)W_{34}	**S4**
Figure S3 – Distortion parameters schematics and calculation information	**S5**
Figure S4 – Distortion parameters comparison between Tb(IV)W_{34} and Tb(III)W_{34}	**S6**
Figure S5 – Time dependent DFT plots of Tb(III)W_{34} and Tb(IV)W_{34} absorption spectra and corresponding natural transition orbitals	**S7**
Figure S6 – UV-Vis-NIR spectra of Ce(III)W_{34} and Ce(IV)W_{34} and pictures of solution of Ce(III)W_{34} before and after $K_2S_2O_8$ addition	**S8**
Figure S7 – Tb L_3-edge XANES time resolved data for Tb(IV)W_{34}	**S9**
Table S3 – Summary of inflection point and peak maximum energies obtained from Tb L_3-edge XANES measurements	**S10**
Figure S8 – Comparison of X-band EPR spectra for Tb(IV)W_{34} and Gd(III)W_{34} and comparison of experimental and simulated EPR spectra for Gd(III)W_{34}	**S11**
EPR simulation parameters and scripts for Tb(IV)W_{34} and Gd(III)W_{34}	**S12-S13**
Figure S9 – χ^{-1} vs temperature plots for Tb(IV)W_{34} and Tb(III)W_{34}	**S14**
SQUID data processing section	**S15-S16**
Figure S10 – Total and partial Tb f-orbital density of states plots for Tb(III)W_{34} and Tb(IV)W_{34}	**S17**
DFT coordinates for Tb(III)W_{34} and Tb(IV)W_{34}	**S18-S23**
Table S5 - pH of capped and uncapped UV-Vis-NIR stability study samples	**S24**
Figure S11 - SEM-EDS spectrum for Tb(IV)W_{34}	**S25**
Table S6 - Weight % data SEM-EDS spectrum collected on Tb(IV)W_{34}	**S26**
References	**S27**

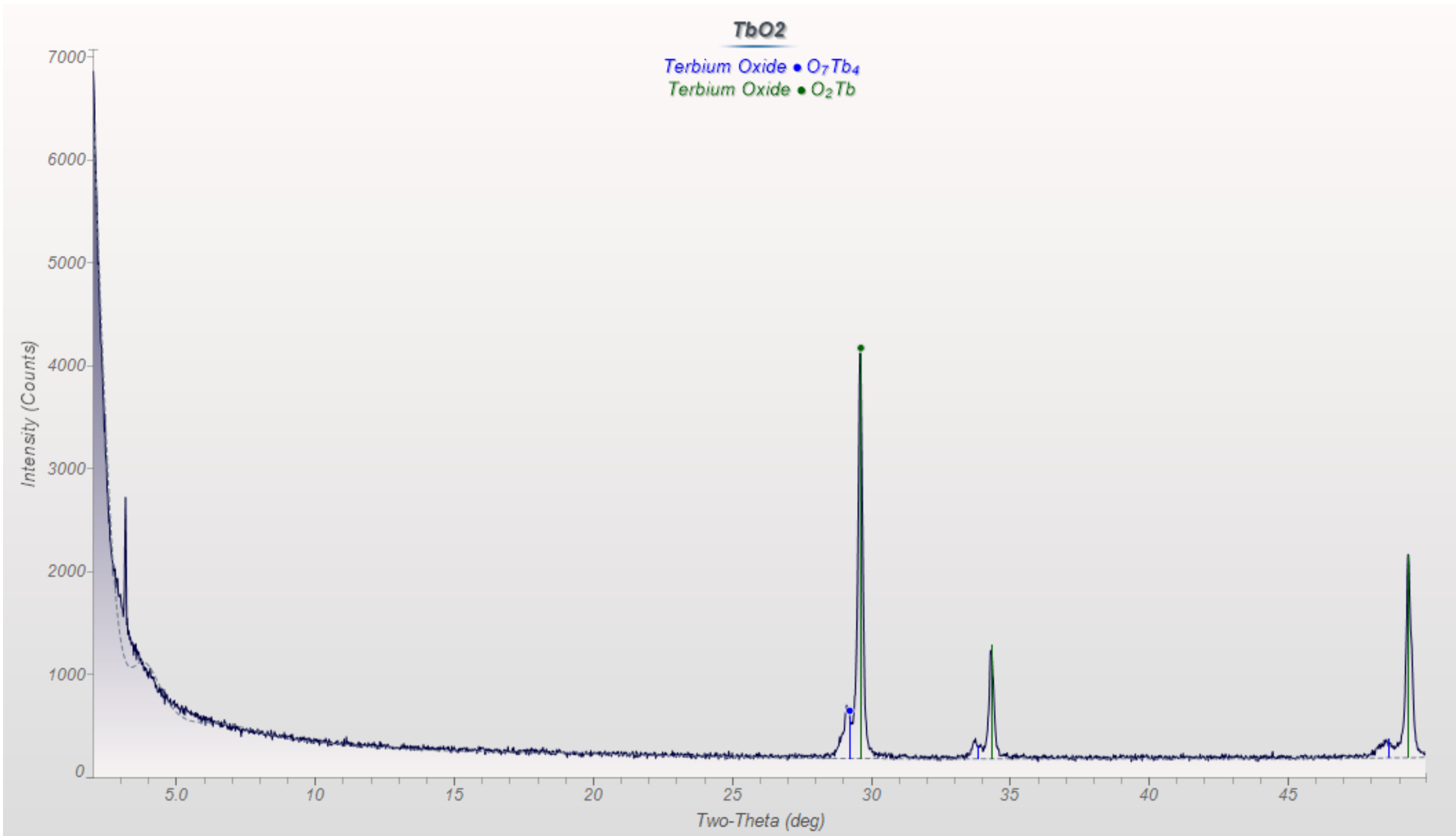

Figure S1. PXRD pattern of Tb(IV)O_2 (black trace) indexed with known PXRD patterns of Tb(IV)O_2 (green) and mixed valent Tb_4O_7 (blue) from the ICDD database.[1,2]

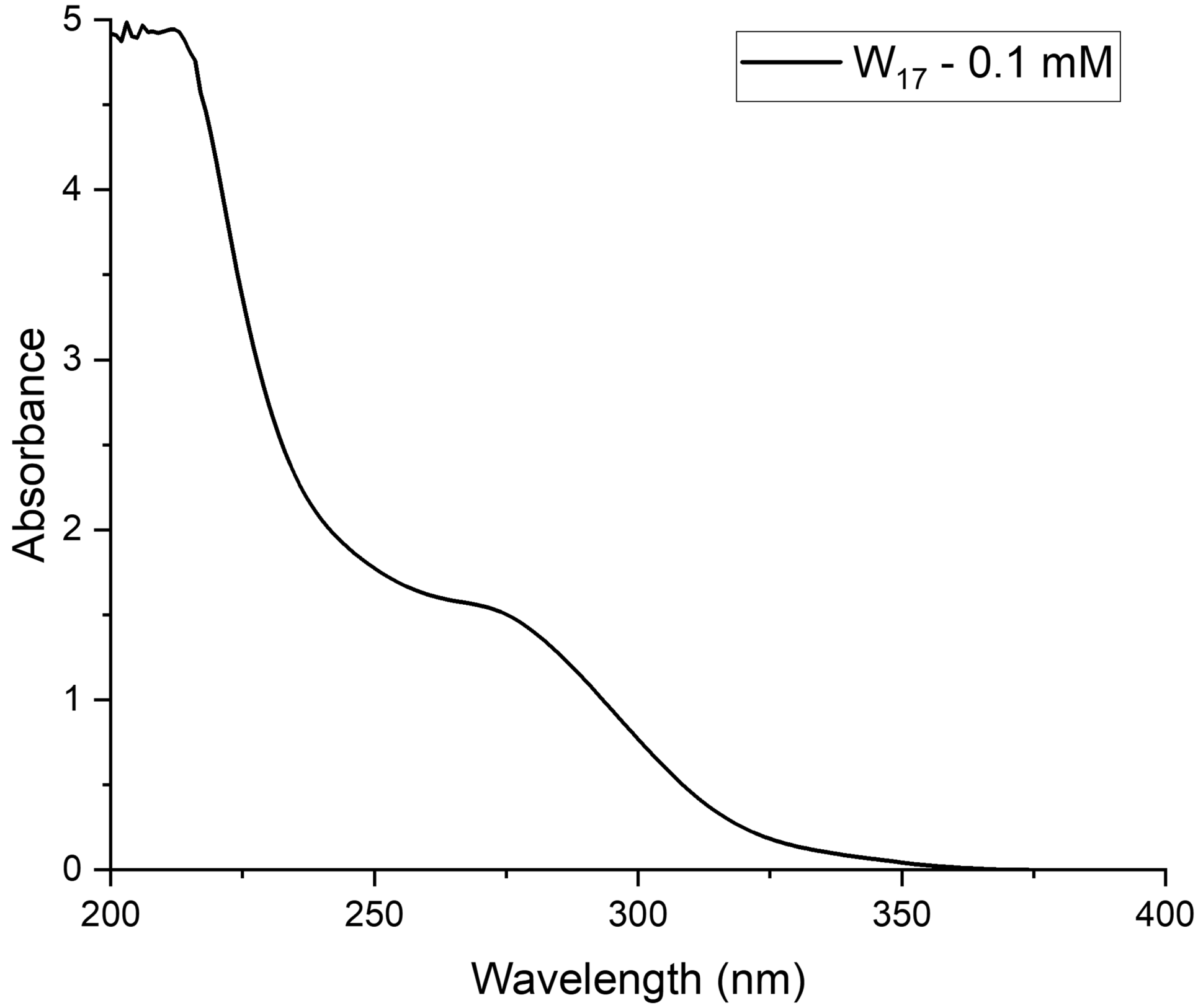

Figure S2. UV-Vis-NIR spectrum of $K_{10}P_2W_{17}O_{61}$•$20H_2O$ in water showing the absorption band centered at around 280 nm. Data collection was done at room temperature in a 1 cm path length quartz cuvette without any background electrolyte.

Table S1. Crystallographic parameters for Tb(IV)W_{34}, Tb(III)W_{34}, Ce(IV)W_{34}, Ce(III)W_{34}, Ce(IV) polymorph (Ce(IV)W_{34}_version II), Gd(III)W_{34}, and Dy(III)W_{34}.

	Tb(IV)W_{34}	Tb(III)W_{34}	Ce(IV)W_{34}	Ce(III)W_{34}	Ce(IV)W_{34}_version II	Gd(III)W_{34}	Dy(III)W_{34}
Formula	$K_{16}TbP_4W_{34}O_{122}$ • 39.85 H_2O	$K_{17}TbP_4W_{34}O_{122}$ • 42.5H_2O	$K_{16}CeP_4W_{34}O_{122}$ • 28 H_2O	$K_{17}CeP_4W_{34}O_{122}$ • 5 H_2O	$K_{16}CeP_4W_{34}O_{122}$ • 32.2 H_2O	$K_{17}GdP_4W_{34}O_{122}$ • 36.3 H_2O	$K_{17}DyP_4W_{34}O_{122}$ • 38 H_2O
Mr	9736.7	9830.4	9556.5	9101.04	9624.42	9729.45	9709.9
SG	*C*2/*c*	*P*-1	*P*-1	*P*2₁/*n*	*C*2/*c*	*P*-1	*P*-1
a (Å)	47.5405(17)	14.5231(4)	14.4784(7)	12.2641(9)	47.6786(19)	14.4440(5)	13.6456(5)
b (Å)	14.5404(5)	22.4915(9)	22.4528(12)	23.2195(17)	14.5620(5)	22.3894(10)	21.6748(9)
c (Å)	22.7790(8)	24.6265(9)	24.5040(14)	51.563(4)	22.8411(8)	24.5645(11)	24.7194(8)
α (º)	90	95.662(2)	95.552(2)	90	90	95.292(2)	90.405(2)
β (º)	91.484(1)	102.669(1)	102.574(2)	91.253(2)	91.560(1)	102.151(2)	91.891(1)
γ (º)	90	99.523(1)	100.428(2)	90	90	100.519(2)	100.437(2)
V (Å^3)	15740.9(10)	7663.7(5)	7569.0(7)	14680.0(19)	15852.6(10)	7564.6(6)	7185.6(5)
R_{int}	0.0488	0.0811	0.1018	0.1457	0.0757	0.1002	0.0915
R1	0.0319	0.0332	0.0654	0.1099	0.0414	0.0407	0.0321
wR2	0.0727	0.0792	0.1639	0.2933	0.1091	0.1074	0.0780
GooF	1.147	1.066	1.203	1.132	1.048	1.089	1.018
μ (mm^{-1})	25.754	26.479	26.605	27.393	25.409	26.791	28.262
F000	16935	8560	8292	15983	16720	8459	8438
Z	4	2	2	4	4	2	2
D_{calc} (g/cm^3)	4.109	4.260	4.193	4.118	4.033	4.272	4.488
Temp. (K)	100 (2)	100 (2)	100 (2)	100 (2)	100 (2)	100 (2)	100 (2)
Wavelength (Å)	0.71073	0.71073	0.71073	0.71073	0.71073	0.71073	0.71073

Table S2. Average bond distances and calculated bond valence summations for Tb(IV)W_{34} and Tb(III)W_{34}.

**Complexes**	**d_{Ln-O}**	**Bond Valence**
Tb(IV)W_{34}	2.279(7) Å	4.11
Tb(III)W_{34}	2.377(6) Å	3.16

Bond valence sum calculations were conducted using the PLATON software in the Olex 1.5 graphical user interface.[3,4]

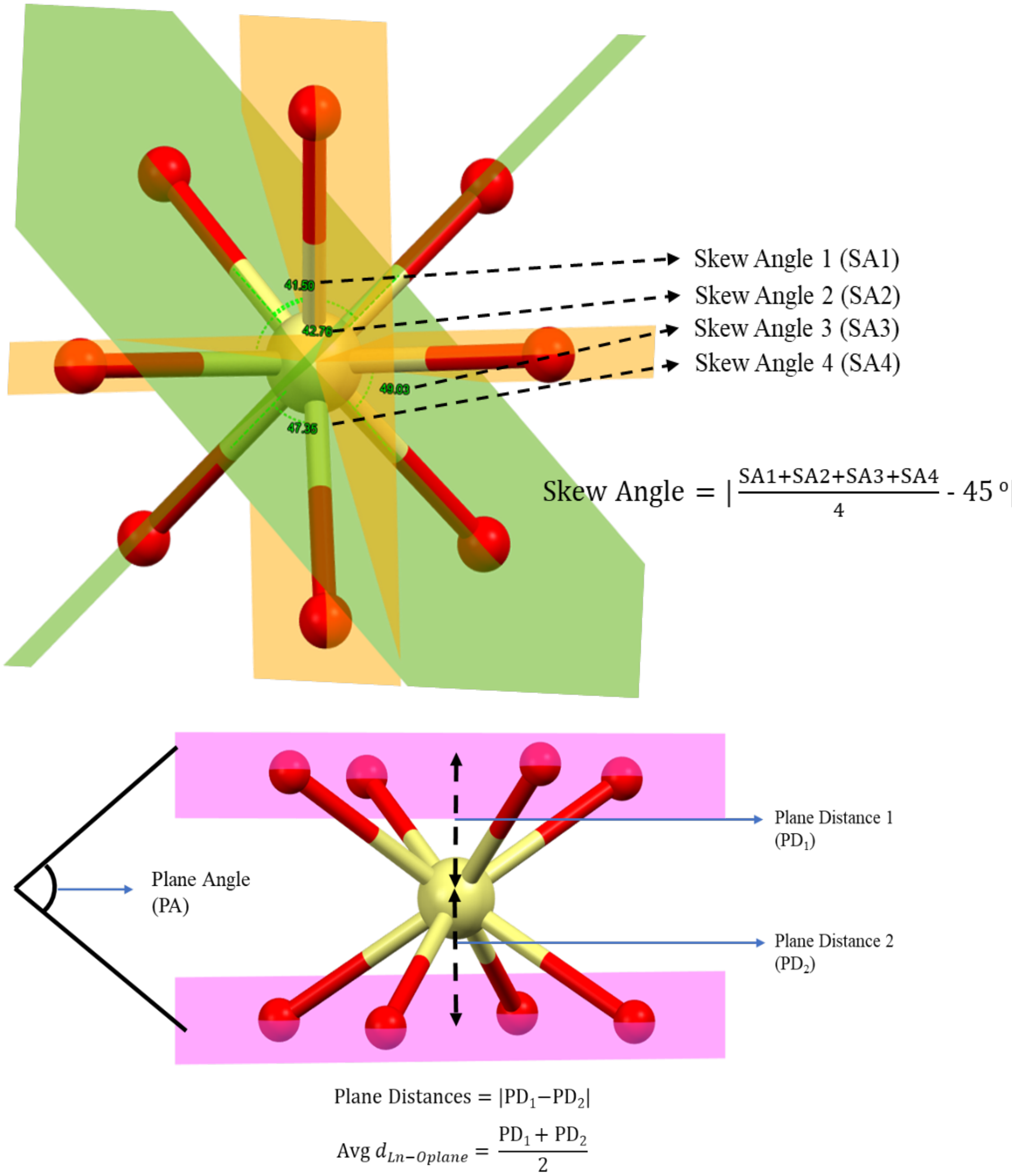

Figure S3. (**Top**) Top-down view of ball and stick representation of TbO_8 moiety shown with calculated planes used to determine skew angle values in Tb(IV)W_{34} and Tb(III)W_{34}. (**Bottom**) Side view of ball and stick representation of TbO_8 moiety displayed with calculated planes used to determine plane angle and distance values in Tb(IV)W_{34} and Tb(III)W_{34}.

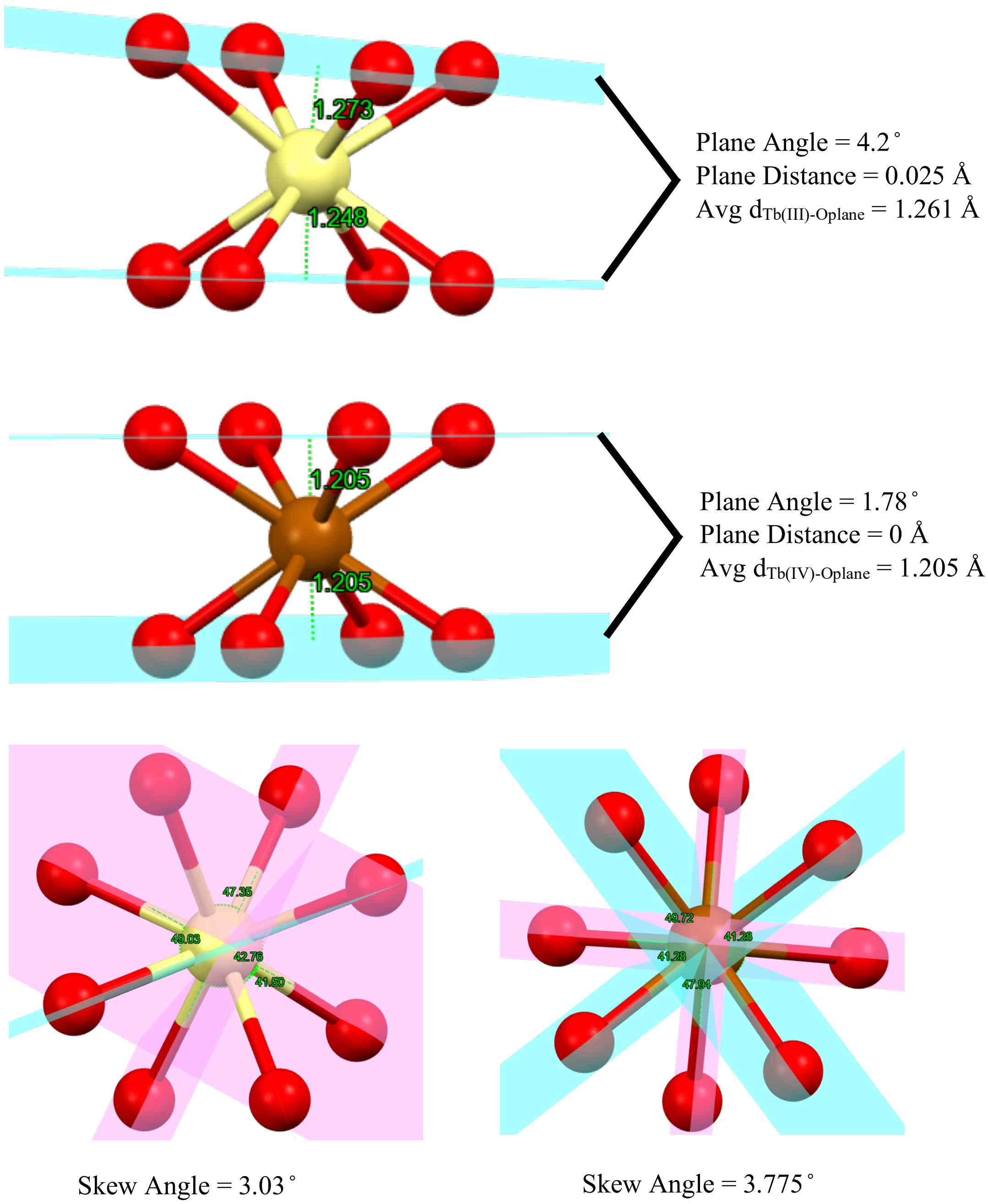

Figure S4. (**Top**) Distortion parameter figures for Tb(III)W_{34} and Tb(IV)W_{34}. Red spheres represent oxygen atoms, while yellow and brown spheres represent Tb(III) and Tb(IV), respectively. **(Bottom Left)** Skew angle values are shown for Tb(III)W_{34} and are 47.35 º, 49.03 º, 42.76 º, and 41.50 º. (**Bottom Right)** Skew angle values are shown for Tb(IV)W_{34} and are 41.28 º, 41.28 º, 47.94 º, and 49.72 º.

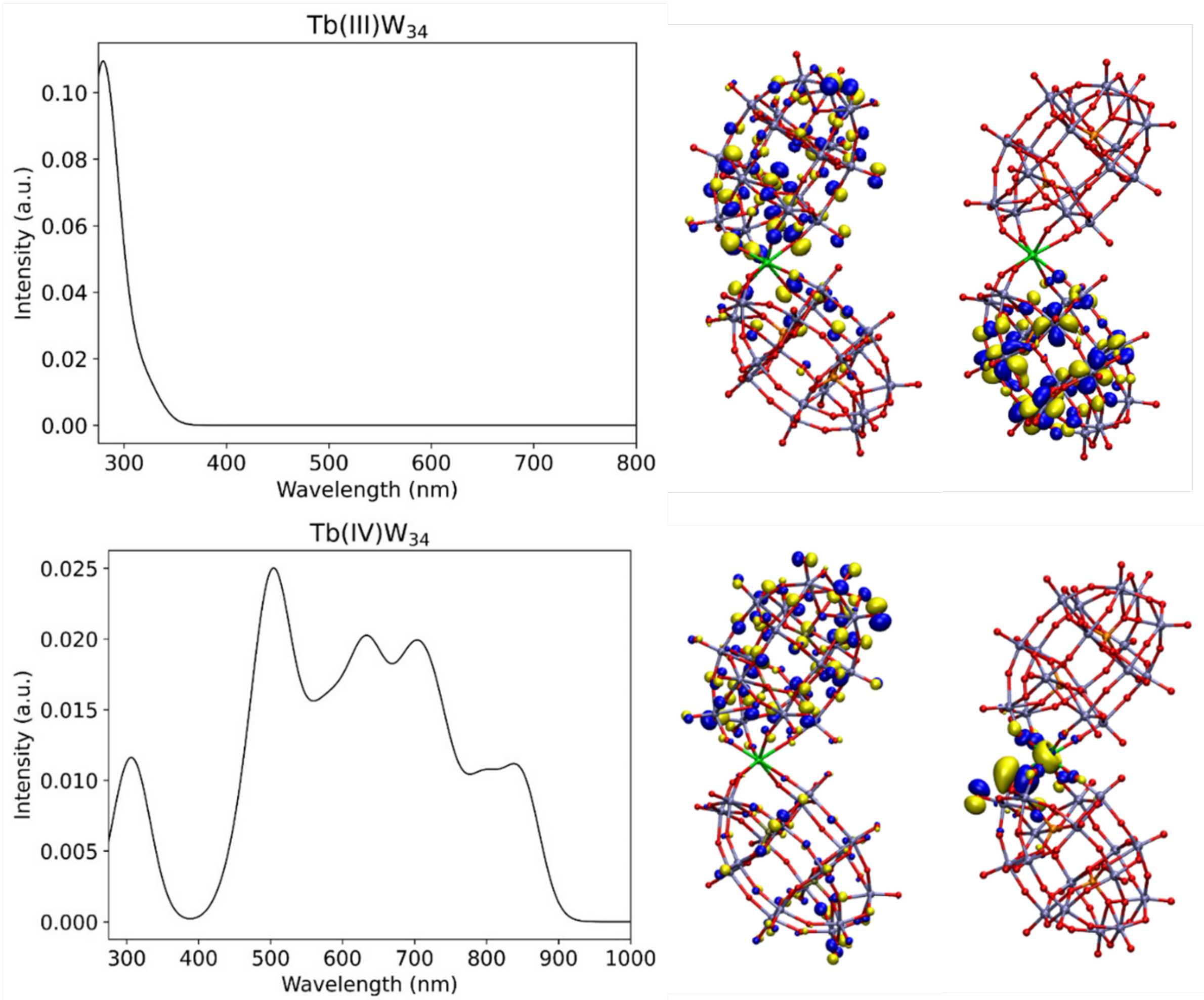

Figure S5. (**Top Left**) Time-dependent density functional theory (TDDFT) plot for Tb(III)W_{34} excitations, which was restricted to orbitals from HOMO -100 to LUMO +100. Only the first 150 excitations were calculated. (**Top Right**) Highest occupied and lowest unoccupied natural transition orbitals for Tb(III)W_{34} excitation at 297 nm. Results indicate this high energy feature originates from oxygen to tungsten absorptions in the W_{34} POMs. (**Bottom Left**) Time-dependent density functional theory plot for Tb(IV)W_{34} excitations, which was restricted to orbitals from HOMO -100 to LUMO +100. Only the first 150 excitations were calculated. (**Bottom Right**) Highest occupied and lowest unoccupied natural transition orbitals for Tb(IV)W_{34} excitation at 509 nm. Results indicate this visible energy feature is a result of oxygen to terbium ligand-to-metal charge transfer. Color code: terbium in green, tungsten in gray, phosphorous in orange, oxygen in red, and NTOs in blue and yellow.

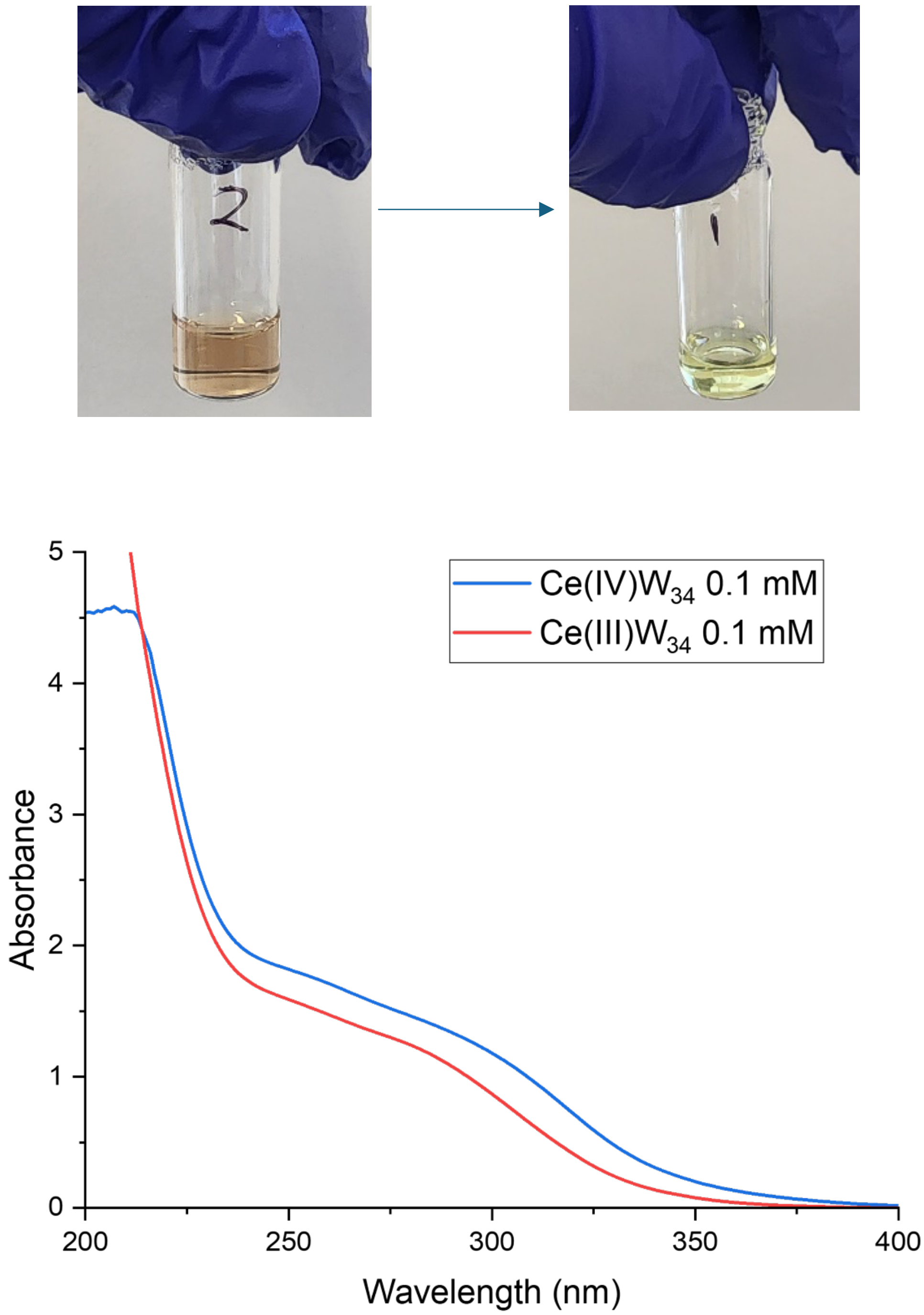

Figure S6. (**Top Left**) Dark brown solution containing Ce(III)W_{34}. (**Top Right**) Light yellow solution produced after heating a solution containing Ce(III)W_{34} with $K_2S_2O_8$ to produce Ce(IV)W_{34}. (**Bottom**) UV-Vis-NIR spectra of Ce(III)W_{34} and Ce(IV)W_{34} showing a redshift in the absorption onset of the POM band upon Ce oxidation.

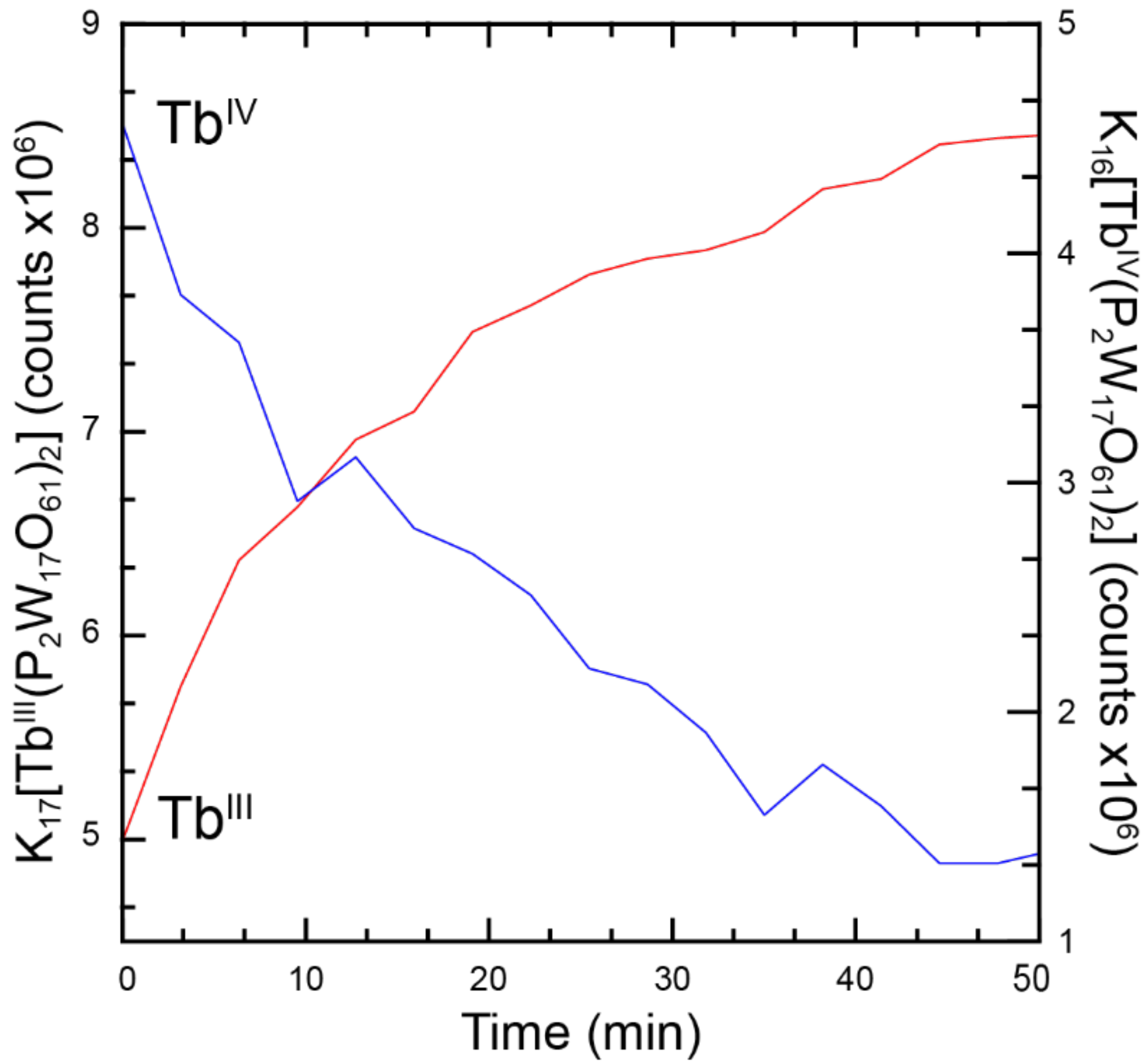

Figure S7. Plot showing disappearance of Tb(IV)W_{34} and in-growth of the Tb(III) decomposition product as a function of time after exposure to the incident X-ray beam during Tb L_3-edge XANES data acquisition. Disappearance of Tb(IV)W_{34} (blue trace) was determined by monitoring the decrease in total counts at the Tb(IV) high-energy peak maximum, 7527.9(1) eV. The in-growth of the Tb(III) decomposition product (red trace) was determined by measuring the increase in total counts at the Tb(III) decomposition peak maximum, 7518.7(1) eV.

Table S3. Inflection point and peak maximum energies obtained from low temperature (10 K) Tb L_3-edge XANES data collected on samples of Tb(IV)W_{34}, Tb(III)W_{34}, Tb(IV)O_2, and Tb(III)$Cl_3 \cdot xH_2O$

**Compound**	**Inflection Point (eV)**	**Peak Maximum (eV)**
$K_{16}[Tb^{IV}(P_2W_{17}O_{61})_2] \cdot xH_2O$	7516.8(1)	7518.7(1)
		7527.9(1)
$Tb^{IV}O_2$	7517.3(1)	7518.8(1)
		7528.8(1)
$K_{17}[Tb^{III}(P_2W_{17}O_{61})_2] \cdot xH_2O$	7516.5(1)	7518.8(1)
$Tb^{III}Cl_3 \cdot xH_2O$	7516.2(1)	7518.5(1)

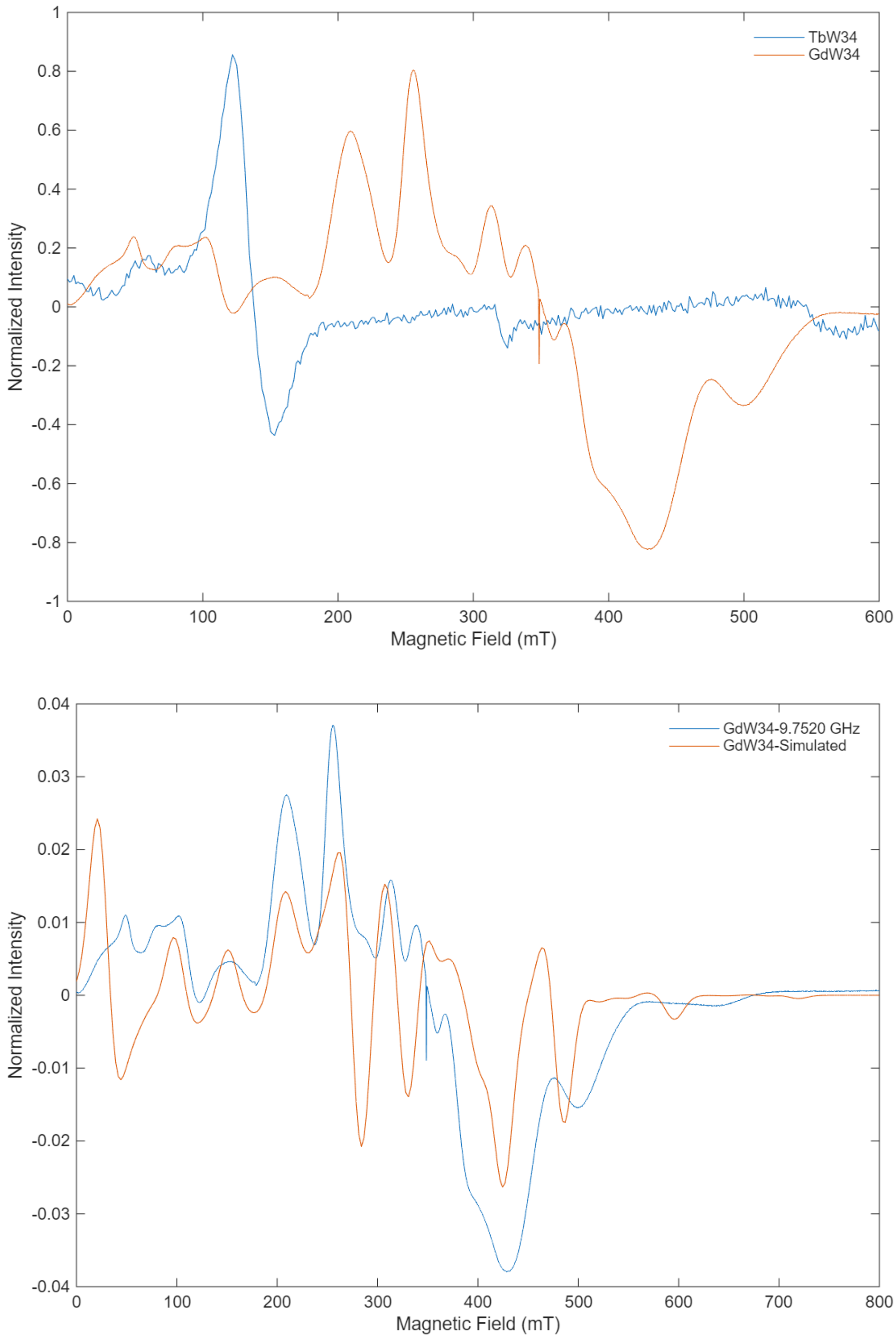

Figure S8. (**Top**) Comparison of low temperature X-band EPR spectra of Tb(IV)W_{34} and Gd(III)W_{34} and (**Bottom**) simulated EPR spectrum for Gd(III)W_{34} (orange trace) obtained with EasySpin 6.0 within the MATLAB interface overlaid with experimental for data for Gd(III)W_{34} (blue trace).[5]

Table S4. EasySpin parameters used for simulations of low temperature CW X-band EPR spectra of Tb(IV)W_{34} and Gd(III)W_{34}.

**Parameters**	Tb(IV)W_{34}	Gd(III)W_{34}
S	7/2	7/2
g	2.01	2.01
lwpp	20	19
D (MHz)	19000	1760
E (MHz)	2000	147

Included below are the MATLAB scripts used to simulate the Tb(IV)W_{34} and Gd(III)W_{34} spectra. The scripts make use of the MATLAB toolbox EasySpin.[5]

Tb(IV)W_{34}

```
clear, clc
[B,spc0] = eprload("Tb4_5mMol_5k_mod.DTA");

Sys.Nucs = 'Tb';

Sys.S = 7/2;

Sys.A = 0;
Sys.g = 2.01;
Sys.D = [19000 2000];

Sys.lwpp = 20;

Exp.mwFreq = 9.483022;

Exp.Range = [0 1600];

Exp.Temperature = 5;

Exp.ModAmp = 1;

Exp.Harmon = 1;
Exp.Mode = 'perpendicular';

Opt.GridSize = 50;

[B,spc1] = pepper(Sys,Exp,Opt);

plot(B,spc0,B,spc1*500);
xlabel('Field (mT)');
ylabel('Intensity (counts)');
```

Gd(III)W_{34}

```
clear
clc
[B,spc] = eprload("GdW34_9K_24dB_9.7520GHz_7scans_20250718.DTA");
B = B/10;

spc = spc/100;
sys.S = 7/2;

sys.g = 2.01;

sys.lwpp = 19;

D = 1760;%MHz
E = D/12;%MHz

sys.B2 = [E 0 D/3 0 0];

exp.nPoints = 385;

exp.Temperature = 9; %K

exp.mwFreq = 9.7520; %GHz

exp.Range = [0 1000];

exp.Harmonic = 1;

[B1,spc1] = pepper(sys,exp);
plot(B,spc,B1,spc1);
```

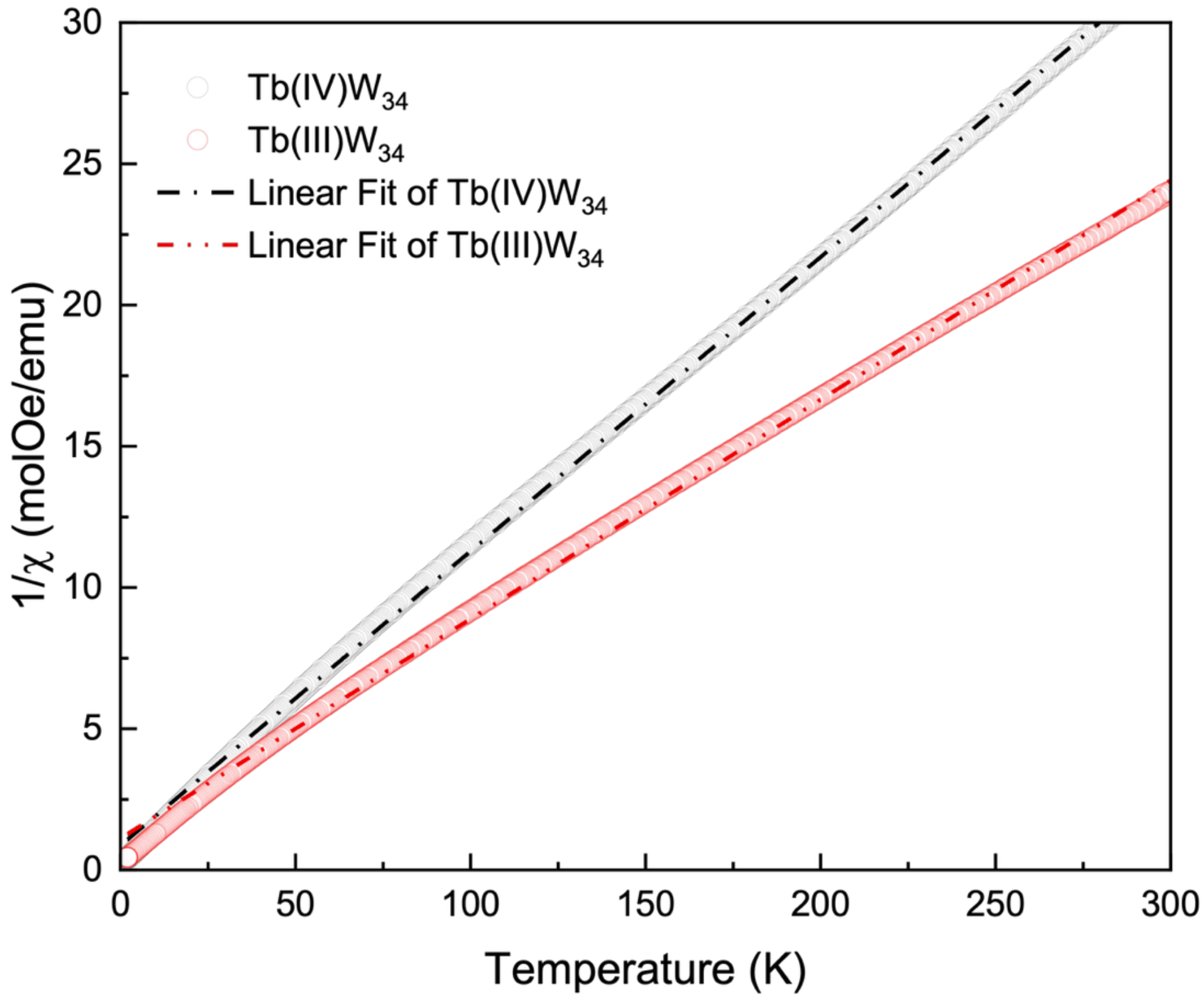

y = (0.868461) + (0.104153) * x	
	Tb(IV)W_{34} (**1**)
Pearson's r	0.9997
Adj. R-Square	0.99939
Intercept	0.86846 ± 0.0133
Slope	0.10415 ± 7.86133E-5

y = (1.11976) + (0.0776552) * x	
	Tb(III)W_{34} (**1b**)
Pearson's r	0.99915
Adj. R-Square	0.99831
Intercept	1.11976 ± 0.01664
Slope	0.07766 ± 9.83193E-5

Figure S9. 1/χ versus temperature plots for Tb(IV)W_{34} and Tb(III)W_{34} with linear fits and the tables associated with the linear regression analysis.

Effective magnetic moment (μ_{eff}) values were extracted using **Eq. S1** in which the Curie constant (C) can be calculated by assuming the line equation from the linear fit regression of the χ^{-1} vs T graphs follows the Curie-Weiss equation (**Eq. S2**). This assumption is acceptable if the χ^{-1} vs T graphs for Tb(IV)W_{34} and Tb(III)W_{34} show strong linear fits.[6] Diamagnetic corrections were done following the work of Bain and Berry.[7] The calculated magnetic moment values (μ_{calc}) for Tb(IV) and Tb(III) were obtained using **Eq. S3** and g_J values published in the work of Muragineza and Hallas.[6]

$$\mu_{eff} = \sqrt{8C}\mu_B \qquad \textbf{Eq. S1}$$

μ_{eff} = effective moment of the sample

C = Curie constant calculated from **Eq. 2**

μ_B = Bohr Magneton is the unit of μ_{eff}.

$$\chi^{-1} = \frac{T}{C} - \frac{\theta_{CW}}{C} \qquad \textbf{Eq. S2}$$

χ^{-1} = Inverse magnetic susceptibility (χ)

T = Temperature (X-value of the linear regression equation)

C = Curie Constant (Extracted from the linear regression equation following Slope = 1/C)

θ_{CW} = Curie-Weiss Temperature (X-intercept of the linear regression equation)

$$\mu_{calc} = \mathbf{g_J}\sqrt{J(J+1)}\,\mu_B \qquad \textbf{Eq. S3}$$

$\mathbf{g_J}$ = g-tensor obtained from the work of Muragineza and Hallas[6]

J = Total angular momentum

From **Eq. S1** and **Eq. S2** along with the linear fits extracted from the χ^{-1} vs T graphs, the μ_{eff} values of Tb(IV)W_{34} and Tb(III)W_{34} were determined to be 8.76 μ_B and 10.15 μ_B, respectively. The μ_{calc} value for Tb(III) is 9.74 μ_B, while the calculated magnetic moment for f^7, $^8S_{7/2}$ Tb(IV) is 7.94 μ_B.

Sequences used to conduct SQUID magnetometry on Tb(IV)W_{34} and Tb(III)W_{34}. Both zero field cooled (ZFC) and field cooled (FC) data for Tb(IV)W_{34} and Tb(III)W_{34} were collected, and the highest quality data was used for fitting and analysis. For Tb(IV)W_{34} and Tb(III)W_{34} the data collected from the 1000 Oe ZFC variable temperature regime were used to extract χT and $1/\chi$ values.

```
REM Sequence to perform Zero Field Cool and Field Cool measurement at 1000 Oe
REM
REM ***Setting up the instrument***
FLD FIELD 0.0 100.00 2 0
WAI WAITFOR 60 0 1 0 0 0
TMP TEMP 300.000000 12.000000 0
WAI WAITFOR 60 1 1 0 0 0
TMP TEMP 20.000000 12.000000 0
WAI WAITFOR 60 1 1 0 0 0
TMP TEMP 2.000000 3.000000 0
WAI WAITFOR 60 1 1 0 0 0
FLD FIELD 1000.0 20.00 2 0
WAI WAITFOR 60 0 1 0 0 0
REM
REM ***Recording ZFC***
VSMDF "C:\QdSquidVsm\Data\PJS\20250725-TbIVW34\20250725-TbIVW34-ZFC-1000Oe.dat" 0 0 ""
VSMMT 1 0 0 1 0 5 14 2 0 2 0 2 24 10 1 23 50 0 1 30 3 0 "MAP " "A/C,6,10,10,0" "ACM 2 2 10 1 0 2 0 0 2E-006 1 0 0 0 0 ^MAP ^ ^AMP 1^ ^FRQ 7.57,75.7,757^ 0 1 30 4 1 200 0 2 0"
VSMMT 1 0 0 1 0 5 14 2 0 2 0 24 40 10 1 5 50 0 1 30 3 0 "MAP " "A/C,6,10,10,0" "ACM 2 2 10 1 0 2 0 0 2E-006 1 0 0 0 0 ^MAP ^ ^AMP 1^ ^FRQ 7.57,75.7,757^ 0 1 30 4 1 200 0 2 0"
VSMMT 1 0 0 1 0 5 14 2 0 2 0 45 300 10 1 52 50 0 1 30 3 0 "MAP " "A/C,6,10,10,0" "ACM 2 2 10 1 0 2 0 0 2E-006 1 0 0 0 0 ^MAP ^ ^AMP 1^ ^FRQ 7.57,75.7,757^ 0 1 30 4 1 200 0 2 0"
REM
REM ***Setting up the instrument***
WAI WAITFOR 60 0 1 0 0 0
FLD FIELD 1000.0 20.00 2 0
WAI WAITFOR 30 0 1 0 0 0
REM
REM ***Recording FC***
VSMDF "C:\QdSquidVsm\Data\PJS\20250725-TbW34\20250725-TbW34-FC-1000Oe.dat" 0 0 ""
VSMMT 1 0 0 1 0 5 14 2 0 2 0 300 45 10 1 23 50 0 1 30 3 0 "MAP " "A/C,6,10,10,0" "ACM 2 2 10 1 0 2 0 0 2E-006 1 0 0 0 0 ^MAP ^ ^AMP 1^ ^FRQ 7.57,75.7,757^ 0 1 30 4 1 200 0 2 0"
VSMMT 1 0 0 1 0 5 14 2 0 2 0 40 24 10 1 5 50 0 1 30 3 0 "MAP " "A/C,6,10,10,0" "ACM 2 2 10 1 0 2 0 0 2E-006 1 0 0 0 0 ^MAP ^ ^AMP 1^ ^FRQ 7.57,75.7,757^ 0 1 30 4 1 200 0 2 0"
VSMMT 1 0 0 1 0 5 14 2 0 2 0 24 2 10 1 52 50 0 1 30 3 0 "MAP " "A/C,6,10,10,0" "ACM 2 2 10 1 0 2 0 0 2E-006 1 0 0 0 0 ^MAP ^ ^AMP 1^ ^FRQ 7.57,75.7,757^ 0 1 30 4 1 200 0 2 0"
REM
REM ***Preparing the instrument for end of sequence***
TMP TEMP 300.000000 12.000000 0
FLD FIELD 0.0 100.00 2 0
WAI WAITFOR 60 1 1 0 0 0
REM
```

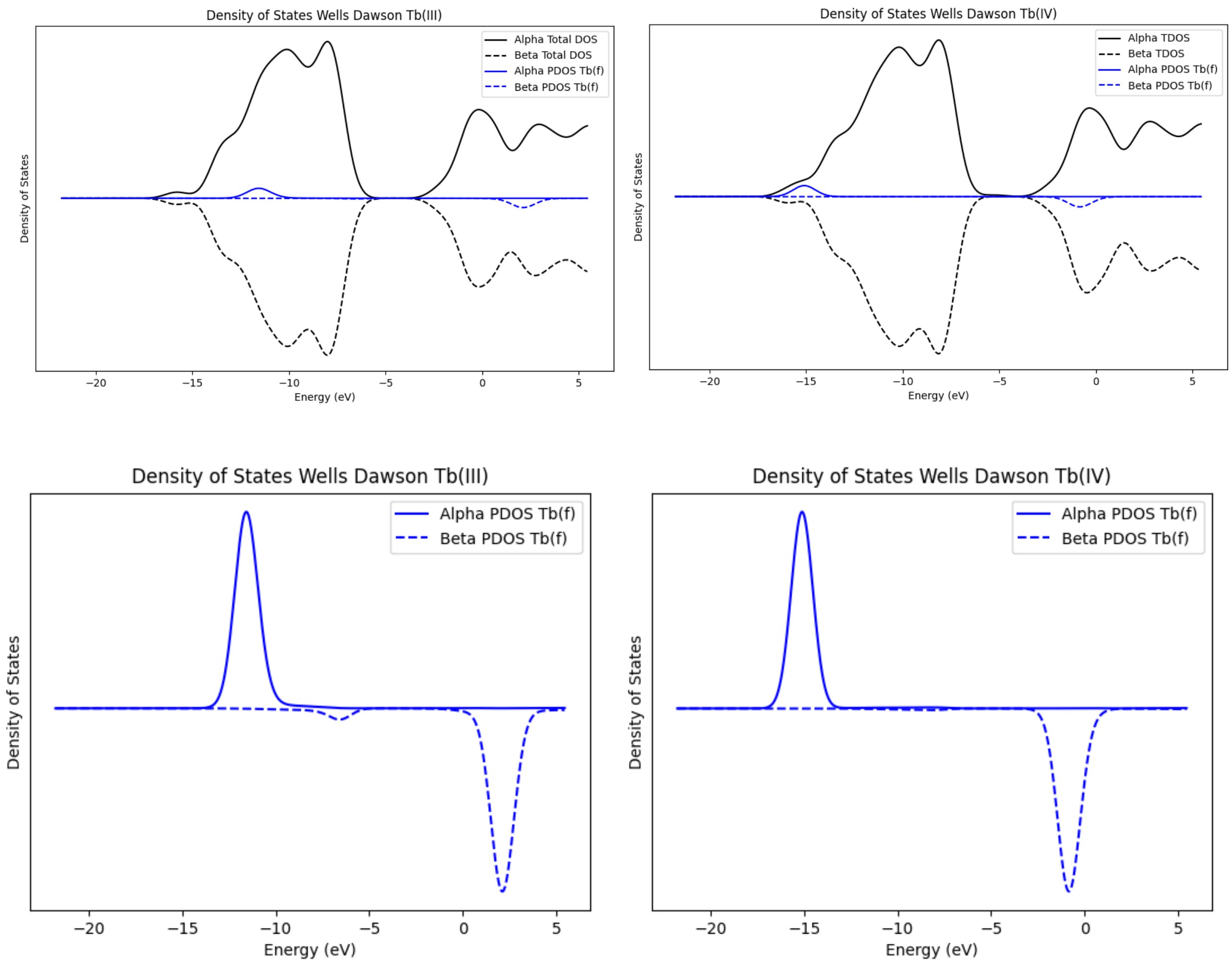

Figure S10. Total and terbium f-orbitals partial density of states for (**Top Left**) Tb(III)W_{34} and (**Top Right**) Tb(IV)W_{34}. Partial density of states of terbium f-orbitals for (**Bottom Left**) Tb(III)W_{34} and (**Bottom Right**) Tb(IV)W_{34}. Energies in eV.

DFT Cartesian Coordinates of $[Tb(P_2W_{17}O_{61})_2]^{17-}$ and $[Tb(P_2W_{17}O_{61})_2]^{16-}$.

$[Tb(P_2W_{17}O_{61})_2]^{17-}$ - Tb(III)W_{34}

W	26.583285	18.632007	18.439082
W	25.130319	17.450534	15.332795
W	28.880070	21.135391	18.377519
W	26.296843	23.216644	16.612034
W	24.952321	22.099110	13.776441
W	28.045235	14.948489	14.886941
W	29.788996	22.738200	15.202156
W	26.095330	18.808083	12.370361
W	29.546260	16.178242	18.071648
W	31.654173	14.288161	15.624027
W	28.434805	21.633644	12.320730
W	28.951827	16.352023	11.950598
W	31.777947	18.714547	18.011112
W	31.337515	19.199236	11.899105
W	32.696968	20.308941	14.781955
W	33.910842	16.859234	15.577638
W	32.559269	15.742161	12.662521
Tb	23.327150	20.699682	17.086176
P	30.508962	17.524833	14.914199
P	27.404644	20.176018	15.339752
O	25.304458	19.798329	18.104212
O	28.305557	17.443056	18.440838
O	24.175957	18.867391	15.776673
O	27.749515	19.935571	19.310983
O	24.979801	17.673673	13.406406
O	26.839329	18.926873	14.591999
O	27.459755	22.273399	17.868762
O	26.086790	17.502013	16.990081
O	30.290277	19.784922	18.406153
O	30.983596	17.200961	18.856789
O	26.624371	16.064205	14.843745
O	30.976940	15.098651	17.160220
O	28.483480	15.469563	16.661139
O	27.869060	19.775044	16.775325
O	29.839627	21.867220	16.895681
O	30.292900	17.738404	16.436932
O	23.988508	16.184089	15.555240
O	29.927407	14.233581	14.932965
O	28.588231	20.811605	14.535291
O	32.218702	19.880931	16.581216
O	27.559204	19.970302	11.993565
O	26.266545	21.248513	15.453817
O	29.472557	22.070136	19.685987
O	31.357049	21.584940	14.757999
O	25.166759	20.362062	12.928065
O	28.116345	23.344888	15.615387
O	29.263957	16.887910	14.239271
O	25.504621	23.577490	14.887799
O	33.033299	17.504574	17.129149
O	25.940273	17.807477	19.807106
O	30.938557	18.837263	14.203627
O	29.504267	22.886673	13.307496
O	32.692445	20.153097	12.870587

O	23.632971	21.574598	14.830256
O	24.806424	22.578582	17.315680
O	33.408648	15.077812	16.042187
O	29.902591	17.968363	11.639284
O	27.377896	17.365672	12.178715
O	26.850392	22.303490	12.918177
O	30.112407	20.561245	12.112075
O	33.653883	18.604122	14.875462
O	28.216996	14.914554	12.962437
O	32.387451	17.560813	12.162705
O	30.719360	15.591771	12.187894
O	34.071695	16.223228	13.753234
O	28.503974	22.336752	10.755678
O	27.280195	13.433255	15.137839
O	32.352462	14.210976	13.791866
O	29.263043	15.051766	19.334180
O	25.361288	18.635044	10.831787
O	30.710424	24.158348	15.489757
O	31.847162	19.504192	10.291236
O	24.133639	22.963382	12.528989
O	34.064541	21.318738	15.001565
O	32.836864	19.243647	19.248635
O	31.710311	16.492106	14.752549
O	26.367859	24.819856	17.244053
O	31.928954	12.667540	16.122805
O	28.680883	15.780594	10.359240
O	35.560059	16.881743	16.048944
O	33.344888	15.049955	11.303798
W	20.069946	18.630805	15.732870
W	21.521387	17.450778	18.840144
W	17.773732	21.135087	15.792191
W	20.354808	23.216588	17.560561
W	21.699045	22.098558	20.396151
W	18.606924	14.948395	19.285668
W	16.861553	22.737059	18.967072
W	20.554334	18.807996	21.802343
W	17.106477	16.177209	16.100200
W	14.998201	14.287347	18.547367
W	18.215737	21.634752	21.849808
W	17.697328	16.352190	22.220928
W	14.875700	18.714262	16.157961
W	15.312541	19.200406	22.270244
W	13.953768	20.307983	19.386274
W	12.741289	16.858098	18.590062
W	14.090346	15.743330	21.507254
P	16.142631	17.524492	19.256279
P	19.246680	20.175701	18.831868
O	21.349578	19.796040	16.067818
O	18.347818	17.441641	15.731156
O	22.475762	18.867473	18.396491
O	18.904777	19.934534	14.860145
O	21.670810	17.674295	20.766971
O	19.812307	18.926680	19.579778
O	19.193100	22.273078	16.301694
O	20.565547	17.500977	17.182769
O	16.363782	19.784013	15.763375

O	15.670241	17.200137	15.313422
O	20.027832	16.063467	19.329985
O	15.675690	15.098206	17.010931
O	18.168984	15.469025	17.511672
O	18.784146	19.774971	17.395918
O	16.812667	21.866871	17.273293
O	16.360138	17.738067	17.733797
O	22.663674	16.184423	18.618969
O	16.724105	14.232417	19.238611
O	18.062291	20.810651	19.635628
O	14.433951	19.880956	17.587217
O	19.090945	19.971277	22.177742
O	20.383897	21.249464	18.720161
O	17.181861	22.069136	14.482923
O	15.294289	21.584428	19.410943
O	21.483685	20.362318	21.244820
O	18.535120	23.343722	18.554681
O	17.387000	16.887216	19.932250
O	21.147635	23.577488	19.284398
O	13.619988	17.504432	17.039756
O	20.713222	17.805644	14.365337
O	15.712658	18.836947	19.966500
O	17.146699	22.886705	20.861870
O	13.957934	20.153421	21.297867
O	23.018551	21.574169	19.342463
O	21.844854	22.576302	16.857976
O	13.243358	15.077387	18.126518
O	16.746605	17.968591	22.530882
O	19.271858	17.365949	21.993613
O	19.800427	22.304618	21.253844
O	16.537731	20.561776	22.058258
O	12.998040	18.603989	19.292526
O	18.432984	14.914559	21.210035
O	14.262057	17.561314	22.006895
O	15.930069	15.591751	21.983009
O	12.578940	16.223795	20.414897
O	18.144958	22.338056	23.414727
O	19.371478	13.432775	19.035522
O	14.297640	14.211414	20.377980
O	17.390631	15.050425	14.838178
O	21.288074	18.635625	23.341132
O	15.940886	24.157613	18.679042
O	14.801820	19.505147	23.877803
O	22.517707	22.963110	21.643528
O	12.586786	21.318297	19.165594
O	13.817600	19.242765	14.919514
O	14.941124	16.491846	19.417211
O	20.285771	24.819651	16.927728
O	14.723112	12.666869	18.048318
O	17.967580	15.781448	23.812621
O	11.092398	16.881271	18.117733
O	13.303471	15.051039	22.865174

$[Tb(P_2W_{17}O_{61})_2]^{16-}$ - Tb(IV)W_{34}

W	26.537173	4.104839	18.463441
W	25.082822	2.898433	15.373862
W	28.817006	6.619860	18.395681
W	26.219768	8.660822	16.621284
W	24.864987	7.509692	13.813336
W	28.007515	0.418894	14.924944
W	29.693453	8.217083	15.203376
W	26.019129	4.234540	12.397353
W	29.511924	1.677589	18.095989
W	31.624419	-0.203607	15.646961
W	28.326331	7.091741	12.334394
W	28.895517	1.810519	11.977307
W	31.728070	4.226532	18.017394
W	31.244124	4.685776	11.905949
W	32.613190	5.816069	14.777894
W	33.862095	2.382304	15.580504
W	32.509513	1.246368	12.677185
Tb	23.323214	6.160352	17.104768
P	30.447110	3.017836	14.930240
P	27.344628	5.623569	15.360252
O	25.244785	5.260938	18.100479
O	28.259326	2.933860	18.470847
O	24.138261	4.325696	15.823443
O	27.685610	5.422395	19.332097
O	24.913420	3.107444	13.448719
O	26.785855	4.368456	14.619170
O	27.377357	7.741266	17.886618
O	26.044513	2.958112	17.028345
O	30.229974	5.286517	18.421451
O	30.944288	2.714275	18.872467
O	26.572926	1.519954	14.883921
O	30.947524	0.608920	17.182077
O	28.448761	0.956269	16.693380
O	27.815427	5.238252	16.797405
O	29.752585	7.362730	16.903769
O	30.236804	3.234580	16.453647
O	23.937720	1.638890	15.606790
O	29.895638	-0.275448	14.963525
O	28.513885	6.273945	14.548571
O	32.140399	5.395455	16.579977
O	27.459601	5.423171	12.015630
O	26.197992	6.685671	15.476954
O	29.400819	7.568114	19.697057
O	31.258387	7.087976	14.755203
O	25.072945	5.782226	12.969742
O	28.004384	8.807788	15.628637
O	29.205136	2.367316	14.262600
O	25.399075	9.005517	14.909363
O	32.984730	3.028772	17.133135
O	25.891136	3.296774	19.837575
O	30.861140	4.331753	14.212659
O	29.376668	8.364878	13.314061
O	32.592552	5.659301	12.867746
O	23.584641	6.964124	14.930779
O	24.726488	7.970419	17.310431

O	33.376245	0.602318	16.053951
O	29.819480	3.442731	11.658980
O	27.306510	2.810856	12.205390
O	26.728409	7.742510	12.945447
O	30.006085	6.043022	12.117450
O	33.583309	4.126120	14.873758
O	28.170735	0.377139	12.999665
O	32.311368	3.061325	12.170856
O	30.666465	1.074491	12.210723
O	34.021146	1.746871	13.757483
O	28.367572	7.797318	10.771152
O	27.258631	-1.101429	15.187167
O	32.318247	-0.280597	13.812434
O	29.244120	0.552716	19.361923
O	25.273567	4.059880	10.865542
O	30.588833	9.653910	15.480406
O	31.742029	4.990291	10.295401
O	23.984859	8.351487	12.595551
O	33.968911	6.842168	14.986169
O	32.788359	4.771377	19.245584
O	31.658768	1.997038	14.766726
O	26.233357	10.258019	17.266310
O	31.914810	-1.818871	16.151311
O	28.622698	1.231636	10.389909
O	35.511897	2.422635	16.044655
O	33.294974	0.557861	11.317702
W	20.153435	4.086774	15.737979
W	21.579836	2.912357	18.853068
W	17.873804	6.600518	15.765902
W	20.354558	8.665731	17.643078
W	21.787498	7.511843	20.421135
W	18.656511	0.430745	19.263850
W	16.895606	8.192910	18.959260
W	20.598204	4.245414	21.819616
W	17.176567	1.673229	16.075393
W	15.050576	-0.207349	18.507032
W	18.298690	7.108804	21.836950
W	17.719809	1.821898	22.197200
W	14.954033	4.218985	16.121606
W	15.377133	4.702637	22.229309
W	14.001880	5.788903	19.341816
W	12.791092	2.355623	18.526134
W	14.117792	1.262044	21.455002
P	16.204614	3.022882	19.220900
P	19.305994	5.621967	18.824451
O	21.426749	5.263969	16.121368
O	18.435871	2.930885	15.710997
O	22.511711	4.350493	18.406467
O	19.020223	5.411610	14.850968
O	21.721757	3.127931	20.779086
O	19.862229	4.379307	19.581517
O	19.313018	7.723670	16.315347
O	20.639890	2.956042	17.182519
O	16.460303	5.288852	15.733753
O	15.755963	2.714052	15.277912
O	20.088191	1.534031	19.323120

O	15.735249	0.610424	16.975133
O	18.229070	0.961212	17.491141
O	18.852226	5.228863	17.386604
O	16.934903	7.367907	17.254648
O	16.432469	3.237054	17.699862
O	22.739513	1.665047	18.640384
O	16.767166	-0.272805	19.205459
O	18.134709	6.285421	19.619470
O	14.532954	5.399426	17.552196
O	19.155078	5.442320	22.175303
O	20.454973	6.693346	18.714536
O	17.323184	7.560921	14.461496
O	15.378953	7.090312	19.382312
O	21.554155	5.796515	21.258660
O	18.640580	8.832824	18.540255
O	17.439284	2.376781	19.905512
O	21.242035	9.005290	19.303092
O	13.691716	3.031463	16.994560
O	20.828760	3.283829	14.377487
O	15.776126	4.337778	19.928264
O	17.260003	8.379306	20.827770
O	14.030896	5.669029	21.248253
O	23.067029	6.975144	19.312838
O	21.931298	7.940882	16.922303
O	13.291645	0.594070	18.060631
O	16.791433	3.457160	22.497261
O	19.310565	2.827571	21.989618
O	19.900512	7.759338	21.246305
O	16.610265	6.062075	22.022316
O	13.054766	4.124519	19.234675
O	18.462279	0.392454	21.186266
O	14.306514	3.075943	21.951801
O	15.952595	1.086037	21.943047
O	12.614315	1.752446	20.347870
O	18.226171	7.821290	23.394162
O	19.410071	-1.087552	19.013764
O	14.320580	-0.269948	20.322152
O	17.459133	0.548134	14.814943
O	21.322889	4.074317	23.359895
O	16.037268	9.642082	18.656068
O	14.858608	5.010260	23.831316
O	22.651107	8.363720	21.640908
O	12.666211	6.832679	19.112125
O	13.912726	4.763557	14.879387
O	14.992491	1.999721	19.371601
O	20.493308	10.221611	16.918595
O	14.768959	-1.822206	18.001295
O	17.972585	1.247706	23.788025
O	11.150529	2.397002	18.037929
O	13.313480	0.577622	22.803750

Table S5. pH of capped and uncapped UV-Vis-NIR stability study samples

Time	pH of Capped	pH of Uncapped
Day 0	2.61	2.57
Day 1	2.61	2.72
Day 4	2.42	2.33
Day 5	2.53	2.61
Day 7	2.56	2.64
Day 12	2.54	2.64

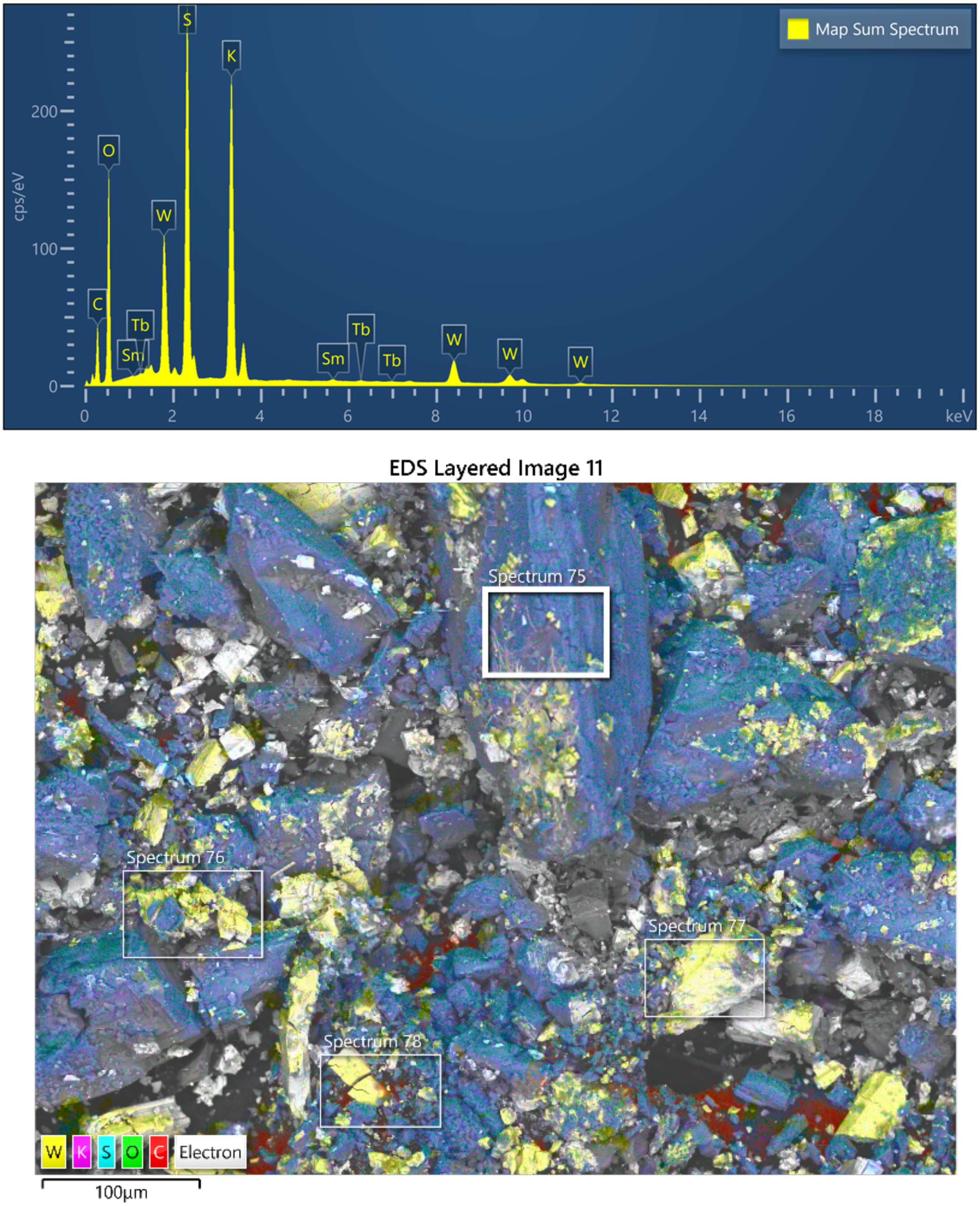

Figure S11. (**Top**) SEM-EDS map sum spectrum collected on freshly harvested crystals of Tb(IV)W_{34}. **(Bottom**) Overall elemental map for Tb(IV)W_{34} obtained via FE-SEM/EDS imaging.

Table S6. Weight % data obtained from SEM-EDS spectrum for Tb(IV)W_{34} synthesis.

Element	Line Type	Weight %	Weight % Sigma	Atomic %
C	K series	14.14	0.09	28.27
O	K series	30.99	0.06	46.50
S	K series	15.34	0.03	11.48
K	K series	17.73	0.03	10.88
Sm	L series	0.44	0.02	0.07
Tb	L series	0.42	0.03	0.06
W	L series	20.94	0.07	2.73
Total		100.00		100.00

Tb(IV)W_{34} Yield Calculation Details

Calculating a (semi-quantitative) synthesis yield for Tb(IV)W_{34} is challenging as a result of the excess $K_2S_2O_8$ that crystallizes at the same time as the main product. To obtain a semi-quantitative yield for the synthesis process, the ratio between $K_2S_2O_8$ and Tb(IV)W_{34} needs to be determined, and we used scanning electron microscope (SEM) – energy dispersive spectroscopy (EDS) to achieve this aim. The yield value for Tb(IV)W_{34} was calculated based on a 0.1 mmol synthesis of $K_{16}Tb^{IV}(P_2W_{17}O_{61})_2$•39.85$H_2O$ (**Tb(IV)W_{34}**; molar mass = 9816.3 g/mol). The materials consumed are 1 g of $K_{10}P_2W_{17}O_{61}$•20H_2O (0.203 mmol; molar mass = 4914.6 g/mol), 1 mL 0.1 M Tb(III)Cl_3 solution (0.1 mmol), and 2.7 g of $K_2S_2O_8$ (~10 mmol; molar mass = 270.32 g/mol). If 0.1 mmol of Tb(IV)W_{34} is synthesized and harvested the mass yield will be $mol\ of\ Tb(IV)W_{34}\ \times molar\ mass\ of\ Tb(IV)W_{34}$, which equals 0.9816 g. From a 0.1 mmol scale synthesis, 2.3 g of crude product were harvested. SEM-EDS data show mass percentages of 15.34% and 20.94% for S and W, respectively. By assuming the mass % to be the mass of the crude product in grams and dividing the mass % by the molar mass of the respective atoms we acquire the number of mols for S (0.478 mol of S) and W (0.114 mol of W). 0.478 mol of S is equal to 0.239 mol of $K_2S_2O_8$ and 0.114 mol of W is equal to 0.00335 mol of Tb(IV)W_{34}. By multiplying the mol values obtained for $K_2S_2O_8$ and Tb(IV)W_{34} with their respective molecular weights, the mass % of $K_2S_2O_8$ and Tb(IV)W_{34} within the crude product can be acquired (64.6% for $K_2S_2O_8$ and 32.9% for Tb(IV)W_{34}). Based on the mass % of Tb(IV)W_{34}, 0.757 g of the 2.3 g crude product are Tb(IV)W_{34}. SCXRD results indicate that Tb(IV)W_{34} crystals likely always contain some amount of Tb(III) within them, and first coordination sphere M-O bond distances allow us to estimate that around 70% of the Tb within the Tb(IV)W_{34} crystals are in the +4 oxidation state. Multiplying 0.757 g (quantity of crude product that is Tb(IV)W_{34}) by 70% results in an overall synthetic yield of 0.53 g. Finally, taking the experimental yield and dividing it by the expected yield and multiplying by 100% we arrive at a semi-quantitative yield for Tb(IV)W_{34} of 54%.

References

1. Andreeva, A. & Gilman, I. Polymorphic transitions in rare earth oxides obtained by reactive evaporation. *Inorg. Mater.* **14**, 384-390 (1978).
2. Kunzmann, P. & Eyring, L. On the crystal structures of the fluorite-related intermediate rare-earth oxides. *J. Solid State Chem.* **14**, 229-237 (1975).
3. Dolomanov, O. V., Bourhis, L. J., Gildea, R. J., Howard, J. A. & Puschmann, H. OLEX2: a complete structure solution, refinement and analysis program. *J. Appl. Crystallogr.* **42**, 339-341 (2009).
4. Spek, A. Single-crystal structure validation with the program PLATON. *J. Appl. Crystallogr.* **36**, 7-13 (2003).
5. Stoll, S. & Schweiger, A. EasySpin, a comprehensive software package for spectral simulation and analysis in EPR. *J. Magn. Reson.* **178**, 42-55 (2006).
6. Mugiraneza, S. & Hallas, A. M. Tutorial: a beginner's guide to interpreting magnetic susceptibility data with the Curie-Weiss law. *Commun. Phys.* **5**, 95 (2022).
7. Bain, G. A. & Berry, J. F. Diamagnetic corrections and Pascal's constants. *J. Chem. Ed.* **85**, 532 (2008).
