# Supplementary material for: Structural and spectroscopic characterization of a Tb(IV) polyoxometalate
Source: Nat Commun. 2026 Jul 16;17:8712. doi: 10.1038/s41467-026-75657-7 (PMC13490611; doi:10.1038/s41467-026-75657-7)
Supplement: Supplementary file 2 [file 41467_2026_75657_MOESM2_ESM.pdf]

nature portfolio

Peer Review File

Structural and Spectroscopic Characterization of a Tb(IV) Polyoxometalate

Corresponding Author: Professor Korey Carter

This file contains all reviewer reports in order by version, followed by all author rebuttals in order by version.

Version 0:

Reviewer comments:

Reviewer #1

(Remarks to the Author)
Carter et al. report the thorough characterization of Tb4+ prepared under aqueous conditions supported by the W17 POM. The demonstration that Tb4+ can be formed and have sufficient lifetime under aqueous (not non-aerobic) conditions is an important extension of the detailed physical characterization studies reported by La Pierre, Mazzanti, and Zheng of molecular Tb4+ complexes prepared in non-aqueous and rigorously O2/H2O-free conditions. These characterization studies of TbW34 build from the synthetic methodology reported by Fedoseev and co-workers using persulfate as the oxidant in large excess. While the Tb4+ oxidation state is only barely stable under a variety of conditions necessary for characterization the authors have pursued a rigorous, detailed, and convincing demonstration of the Tb4+ oxidation state. Furthermore, this 8-coordinate near cubic setting provides deep comparative insight to the physical properties of Tb4+ in comparison to 4-coordinate molecular complexes and 8-coordinate cubic oxide settings in oxide materials. Overall, this is an important paper that will be well-cited by the community. It should be published in Nature Communications after the authors consider the following largely presentation issues.

Technical:
1) The reported technical work spanning crystallography, EPR, magnetometry, XAS, and theoretical description are very well executed and well described. While I think the EPR analysis would benefit from high-field/high-frequency analysis (the Gd3+ simulation could be stronger), this EPR analysis work would appropriate for a follow-up study.
2) While the reported analysis is quite thorough, I would like to see some electrochemical analysis. Computational analysis suggests that the 4/3 couple lies just within the aqueous window. Is this really the case? Or is the exclusive utility of persulfate as the oxidant due to a kinetic phenomenon? The latter is suggested by the instability of the complex under aerobic conditions. What are the technical challenges for this measurement?

Presentation:
1) Valence and oxidation state are not interchangeable terms – the authors are referred to IUPAC definitions. This ambiguity though is the fault of the field of f-element coordination chemistry which has routinely used these terms as synonymous. I would recommend that the authors refer to the oxidation state of the ion and in particular avoid phrasings in which the terms are used together (as in the first line of the paper: trivalent oxidation states – which erodes any clear distinction and/or is repetitive).
2) In the introduction the authors choose the non-alphabetical listing “Mazzanti and La Pierre” and provide two blocks of references for these authors in that order. This seems to make some non-scientific point that is not germane to the authors’ paper.
a. I suggest the authors use the alphabetical order as they largely do throughout the rest of the paper and then group the references by topic: citations for Tb4+ (which are then in year of publication) and for Pr4+ (which are then in year of publication)
b. The authors should include initial report of Pr4+ molecular complex by the La Pierre group (Dalton Trans., 2022, 51, 6696-6706) in the references.
c. The authors may also consider noting the extension of this approach for the characterization of a 5+ praseodymium complex: Nature Chemistry volume 17, pages 1005–1010 (2025)
3) The work Zheng and co-workers is elided from the discussion except for one reference grouped with the studies of Mazzanti.
a. The authors are encouraged to include this groups synthetic and physical characterization studies of Pr4+ and Tb4+

complexes:
i. Dalton Trans., 2024,53, 5779-5783
ii. Precis. Chem. 2023, 1, 10, 583–591 (included)
iii. Chem. Sci., 2025,16, 6805-6811
4) The authors describe, “The strategy developed by the groups of Mazzanti and La Pierre for accessing tetravalent Tb and Pr is based on the use of bulky ligands with strong σ and π donating sites that can destabilize the lanthanide f-orbitals, thereby lowering the energetic favorability of the trivalent configuration. The weak field ligands used by both groups also have the overall effect of increasing ligand covalent character such that tetravalent congeners are stabilized, and the thermodynamic cost of oxidation is diminished.”
a. Yes both systems developed by La Pierre and Mazzanti fit this hypothesis, however only La Pierre has articulated this hypothesis and design strategy. The phrasing of this statement should be revised.

Reviewer #2

(Remarks to the Author)
Isolation and Characterization of Tb(IV) in Aqueous Media via Polyoxometalate Encapsulation

Review
The paper presented by Carter and co-workers reports the synthesis and the single crystal structure of a K16Tb(IV)(P2W17O61)2•39.85H2O (Tb(IV)W34) isolated from water and extensive spectroscopic evidence obtained via UV-Vis-NIR, X-ray absorption near edge spectroscopy, EPR spectroscopies and SQUID magnetometry measurements to confirm the presence of Tb in the oxidation state +4.
The work reported revisit some solution studies described in two papers by V. P. Shilov, and A. M. Fedoseev (Radiochemistry 2007 and Russian Journal of General Chemistry, 2022) where the oxidation of Tb(III) by persulfate ions in solutions of unsaturated phosphotungstate K10P2W17O61 was studied by spectrophotometry. Fedoseev and Shilov also investigated stability in water and its dependency from pH by potentiometry.
In the present work Carter and coworker using analogue conditions were able to isolate and fully characterize in the solid state the Tb(IV) complex. The introduction is appropriate and well referenced. The discussion of the various analysis performed is almost always clear and informative and the authors present a good case for the definitive isolation of aqueous stable Tb(IV) species and the nature of its oxidation state. The novelty of the present contribution lies in the isolation of the Tb(IV) complex in appreciable quantities, its XRD characterization and in the subsequent investigation of the lanthanide oxidation state.
This is an important achievement. However, especially considering that the formation of this complex had already been reported, the manuscript needs to report more rigorously the synthetic method used and investigate more in detail the oxidation conditions and the stability of the final complex.
The presentation of the synthetic part of the work lacks the rigor required for publication in a high-level journal. There are technical aspects of the synthetic part that need to be resolved before publication of this manuscript can be published.
In particular, the nature and purity of the Tb(IV) complex used for all the measurements needs to be specified at all time and the pH measured and reported for all measurements carried in solution and for the stability studies.
From reading the manuscript is not clear how the Tb(IV) can be isolated analytically pure and if it can exist in solution in the absence of the oxidizing agent. The stability study is also very important but unconvincing as described. Previous work reported that reduction to Tb(III) by water occurred depending on pH. This is ignored by the authors and instability seems to be assigned to reaction with oxygen?
Below are indicated the specific points that should be addressed before publication in any high-level journal.
i) Concerning the synthesis of K16/17Ln(III/IV)(P2W17O61) xH2O: In the reported procedure by Peacock and Weakley (1971 J. Chem. Soc. A. 1836) I could only find the Ce(III)W34 and Ce(IV)W34 complexes. In light of this, characterization method to assess purity and water content of the isolated crystals (e.g.: TGA for H2O content and ICP for W) should be used for the complexes that were not reported previously. The large amount of water on the isolated bulk compound is especially important for the magnetic studies.
ii) The synthesis of a general complex [Tb(IV)(P2W17O61)2]16- is given in the method while in the main text the formula of the complex is never specified and one has to wait to get to Sample Preparation for Tb L3-edge XAS Measurements to find the formula of the complex (K16[Tb(IV)(P2W17O61)2]·xH2O). However, there is no information on how the formula was established and on how many water molecules are present in the complex. The large amount of water on the isolated bulk compound is important especially for the magnetic studies. The author should use TGA to determine it. Purity of the complex should also generally be established by analytical techniques.
The formula obtained from the X-ray diffraction is also not presented. These issues should be solved, and presentation of the complex should be more rigorous.
Concerning the synthesis I have several key concerns tht should be addressed in full before publication.
a) When conducting synthesis and reactivity in water it is essential to report pH values as they can be very important in determining the reaction outcome and in determining stability. The authors should also investigate the effect of pH on stability. This is a key point and should be addressed for publication. Several authors of this manuscript have long-term experience in working in water and are aware of these problems/requirements.
Another important question is why oxidation is performed with large amounts of K2S2O8(100 equiv) ? The authors should report what happens with lower amounts of oxidizing agent.
The authors state: “After one day, colorless and brown crystals were observed. The colorless crystals were unreacted K2S2O8, while the brown crystals are Tb(IV)W34¬ (0.382 g, 39% yield).”
How can a yield be established when the pure complex cannot be separated from unreacted K2S2O8? Have the authors obtained the complex analytically pure? This is very important. If is not the case, a separation should be pursued.

The authors also need to clearly specify for every following experiments if the measurements were carried out on pure complex or on a mixture of Tb(IV) and oxidizing agent.
When redissolving the pure? solid in water (if this is what is done) the pH of the obtained solution needs to be measured and indicated.
b) I am very concerned about the stability studies.
The authors should specify precisely how they were conducted. How was the sample initially prepared, in glove box or in air? When stating: The brown crystals were stored in the fridge (5 ºC) in a capped container filled with the mother liquor to slow down the auto-reduction process back to Tb(III)W34.
What capped container means? Under inert atmosphere? The authors seem to hint that air is reducing Tb(IV) to Tb(III) and not water as was mentioned in the original work of Fedoseev . It is not clear what they propose, are they proposing that oxygen reduces Tb(IV) ? but water does not? This seems unlikely. Or what else?
Have the authors checked the variation of pH over time in the capped and uncapped vials. Fedoseev and coworkers had clearly indicated a pH dependency of the stability.
The stability is an important aspect here and needs to be studied and presented rigorously before publication.
Other points
i) In the Materials section, the last sentence is dedicated to the synthesis of the potassium POM salts and $K_6P_2W_{18}O_{62}$ is referenced. However, I do not see why, since such salt does not seem to be used in the paper.
ii)The magnetic measurement provided are in nice agreement with Tb(III) and Tb(IV) species. I appreciate the detailed description of the method used to obtain the theoretic magnetic moments and the SQUID sequences used. However, given such a thorough report, I am surprised that, if diamagnetic corrections were applied, such values are not reported. On the same line, I wonder how many molecules of H_2O the authors assume to be present per metal center and on what basis do they assume that.
Moreover the authors state: Effective magnetic moment values for both complexes are broadly consistent with expectations for Tb(IV) and Tb(III) complexes, with the possibility of a small amount of reduction of Tb(IV)W34 occurring during the sample loading process ahead of measurement."
This is ambiguous and it would be interesting to know how this is assessed. Without presenting data for a pure complex the story remains ambiguous. Are the authors sure that the Tb(III) is not always presend?
Was Gd(III) measured for comparison?
iii) I am slightly confused on the necessity to prove that the LMCT band observed at 437 nm is not due to 'unaccounted interactions between $K_2S_2O_8$ and Ln(III)W34 complexes'.
Is the UV-Vis-NIR recorded on a pure sample of Tb(IV)W34 or on a reaction mixture $K_2S_2O_8$? If the latter is the case, was the stability test performed on Tb(IV)W34 performed in presence or absence of excess $K_2S_2O_8$? Was the pH evolution over time measured? Stability tests in the presence of $K_2S_2O_8$ would have little meaning and to evaluate stability in water the test should be done on the pure complex. The effect of pH should also be carefully monitored .
It remains ambiguous if a pure Tb(IV) complex can be obtained and carefully characterized
Why in the Sample Preparation for Tb L3-edge XAS measurements the $K_{16}[Tb(IV)(P_2W_{17}O_{61})_2]\cdot xH_2O$ solution was prepared using $(NH_4)_2S_2O_8$ rather than $K_2S_2O_8$? Please also here indicate pH values for the soliutions or reproducibility cannot be ensured.
For the following statement 'Fedoseev and colleagues have been the only group to pursue this chemistry further;37,38 however, beyond ...' ref. 38 is Hoefdraad H. E. 1975 J. Inorg. Nucl. Chem. is not relevant to the statement, however, ref. 32 is (Fedoseev A. M. Radiochemistry 2007).
Finally, I would recommend this manuscript for publication since it significantly advances our understanding of high-valent lanthanide chemistry in aqueous conditions. Nevertheless, the absence of a clear assessment of the chemical purity of the isolated and analyzed samples raises important questions that should be addressed to strengthen the quality of the study. The synthetic conditions should be clearly described with more details including additional studies (evaluating the effect of different amounts of oxidizing agent on the reaction outcome). The pH should be indicated for all experiments. The stability studies should be carefully carried on the pure compound out and carefully described. The authors should isolate the complexes in anaerobic conditions first and then expose them to air controlling the pH evolution.

Reviewer #3

(Remarks to the Author)
Subintoro et al. present a water-synthesised and more-or-less stable Tb(IV)POM complex. I like the idea and the outcome is reasonably convincing. But, there are some specific pieces of data that do not yet fully convince me. See below. If these can be addressed, and done so in a robust and thorough manner, I would be happy to see this published here.

As a general comment, I like the straight forward presentation of the work, but I dislike the results summary at the end of the introduction - the paper is not really long enough to need this, and I got confused reading it. Just do the standard "herein we use XYZ techniques to show ABC", and then get into the guts, please.

Comments:

Equation 1 has g with a double-headed arrow over the top - this is usually reserved for its role as a matrix in between the magnetic field and spin vectors. Here, however, it is a scalar so should not have the arrow above. Likewise, the B and S should be given as the dot product.

A little more time needs to be devoted to the EPR simulation section. When D >> hv as here, the exact value of D is poorly captured by simulation, especially given the level of reproduction here. Indeed, some quick playing around indicates that I

can get (i) a better simulation by using a significant D-strain (ca. 0.4 cm-1) that broadens the higher-field satellites at ca 250 mT and (ii) that D anywhere between 0.5 and 0.7 gives a reasonable representation of the experiment (all better than the current simulations).

Given the drastic difference between the Gd(III) and Tb(IV) EPR (which is not the case for Eu(II) vs Gd(III) pairs in the same environment), I am not fully convinced yet that this is "evidence" of 4f7. Indeed the ChiT vs T trace has a significant temperature dependence that is not supported by S = 7/2 with D = 0.6 cm-1. Can they get the EPR spectrum of the Tb(III) complex before oxidation? Clearly they can get the magnetometry, so I would like to see what the EPR looks like - of course it might be silent, but it also might not be!

Indeed, the curvature of the ChiT vs T trace is eerily similar to the Tb(III) sample. Could it be a mixture? I guess this is strongly supported by XANES (Fig 3). This needs to be discussed more "head-on" that this sample is likely a mixture under most analytical conditions.

Version 1:

Reviewer comments:

Reviewer #1

(Remarks to the Author)
I am happy with the revisions the authors have made and the careful consideration of the comments and suggestions of all of the reviewers. The manuscript is ready for publication.

Reviewer #2

(Remarks to the Author)
The authors have made some attempt at improving the manuscript by integrating several comments that we put forward, especially regarding pH of the solutions. The authors have now provided the pH at which the reaction was carried out , and indicated that the pH value changes stay unchanged when the reaction is stirred in an open vial ruling out potential effects of pH change .

However, I often find the comments of the authors still rather dismissive with little action taken on the basis that instrumentation or capability to work under inert atmosphere are lacking.
Since the stabilization of Tb(IV) by polyoxometalates had already been reported together with stability studies I would expect more careful and thorough studies when rivisiting this old chemistry prior to resubmission of Nature communications.
In particular since the authors claim “that the compound undergoes autoreduction when exposed to air “ this claim needs to be demonstrated by carrying the reaction in absence of air which should also allow to obtain a more pure product.
It is unacceptable to state that they cannot work under inert conditions (when working with unusual oxidation states this is not an option). Every laboratory can have access to a Schlenk line.

It also seem strange that the authors were not willing to investigate what happens in the presence of stoichiometric amounts of K2S2O8 rather than 100 molar equivalent. One would expect more information from a paper that revisit past work.
But this is even more surprising when one realizes that the final Tb(IV)W34 compound cannot be obtained pure because cannot be separated from the excess K2S2O8 . Separation would be easier if smaller amounts of K2S2O8 could be used.

The authors say “in ambient conditions, debulking K2S2O8 requires prolonged air exposure which leads to Tb reduction back to the +3 oxidation state” . This can be avoided by working in inert conditions. It really makes no sense and is not acceptable for a nature communication or any high level journal to prepare in air an air-sensitive compound!

In one of the answers to the question of purity a worrying note is made: Tb-O bond distances in Tb(IV)W34 indicate that in the solid-state around 70% of the Tb within the crystals are in the +4 oxidation state. This seems to indicate that even in isolated crystallin material not all the Tb are in the +4 oxidation state. This in my opinion reduces significantly the impact of the work and if the authors believe that this is due to performing the synthesis in air they should repeat it under inert conditions.

It appers clearly now that the authors cannot obtain their complex in a pure form, but some amounts of Tb(III) are always present casting significant doubt on the reported characterizations.

How the isolation of the title compound in pure form could be “beyond the scope of this study”?

I find the use of the term 'isolation' potentially misleading as it could lead the reader to think that Tb(IV)W34 can be obtained pure, with no (or negligible) amounts of Tb(III) and persulfate, which is not the case.
Finally the key result here was announced to be the isolation of a water stable Tb(IV) complex but in their reply now they seem to agree that water is likely to act as reductant (not oxygen) as was suggested in the early Fedoseev studies and therefore an excess of persulfate oxidizing agent is always required to keep the complex in the +IV oxidation state.
Notably in reply to our request to establish the stability of pure Tb(IV)W34 in water , they report stability in presence of persulfate.

I do not think the manuscript in its current form is suitable for publication in Nature Communication and this manuscript could be published in an Inorganic Chemistry journal as it is but clearly stating the limitations of the study (no pure compound obtained, stability of the complex only in presence of persulfate, uncertainty on the origins of the low stability in air).

Reviewer #3

(Remarks to the Author)
I am satisfied that the authors have sufficiently considered and responded to my points.

Version 2:

Reviewer comments:

Reviewer #2

(Remarks to the Author)
Reviewer Report
The authors have made several amendments to the manuscript, clarifying that the title compound cannot be isolated in pure form, though the underlying reason remains unclear. While I agree that stabilizing a Tb(IV) polyoxometalate (POM) in water is an important result, several critical questions remain. Below are my responses to the authors' rebuttal:

1. The authors claim that the stabilization of Tb(IV) by POMs has not been previously reported. This is incorrect. As the authors themselves acknowledge in the text, Saprykin et al. documented the solution-state complexation of Tb(IV) with W17 ligands nearly half a century ago, and Fedoseev's group has also pursued this chemistry. However, I fully recognize and commend the authors for utilizing this foundational knowledge to isolate single crystals of Tb(IV) and further characterize them, despite the characterizations being carried out on non-analytically pure compounds.

2. The authors note that their inorganic work is conducted in ambient, aqueous conditions due to a lack of gloveboxes or Schlenk lines. While we respect that not all laboratories possess this specialized equipment, tackling air-sensitive chemistry inherently requires the appropriate conditions and equipment to handle it properly.
Furthermore, the new experiments the authors carried out in an argon atmosphere confirmed that the complex does not undergo autoreduction in air. Therefore, the authors should remove mentions of the complex being "air-sensitive" from the manuscript, as this has been disproved.

3. A serious problem remains: the complex cannot be isolated as a pure compound, but only in the presence of K2S2O8. . (This is often not accepted in high-level journals). Importantly the presence of K2S2O8. . raises some questions: the authors attribute the low stability to various factors (solvation, autoreduction in the absence of oxidants, and air-sensitivity), creating confusion in the manuscript. It is highly possible that the complex is stable only in the presence of an excess of the oxidizing agent. This can only be proven by removing the excess oxidant and testing if the resulting clean crystals are stable in pure water.

4. Suggested Revisions to the Manuscript

To proceed with publication, the authros should make the following revisions:
Clearly state in the manuscript that the Tb(IV) species cannot be isolated without the presence of K2S2O8. This must be explicitly stated in the main text, not just hidden in the methods section.
Clarify Crystal Separation: Indicate whether and when the Tb(IV) crystals were manually separated from K2S2O8. prior to spectroscopic characterization.
Specify Storage Conditions: In the Methods section, specify that the capped container used to store the brown crystals was capped under air.
Incorporate the experiments mentioned in the reply into the manuscript. Specifically, add that experiments using a Schlenk line and an argon glovebox showed similar reduction behavior in single crystals upon removal from the mother liquor.
Please clarify this observation: are the crystals redissolved in pure water, or do they undergo reduction immediately upon removal from the solution?
Clearly discuss whether clean crystals, if separated, maintain their stability in pure water.
Refrain from describing this as an "air-sensitive" compound, as the authors' own data disproves this behavior.

Note: I will not oppose publication if the Editor is satisfied with reporting a non-analytically pure compound, provided the aforementioned limitations are transparently disclosed to the reader.

Open Access This Peer Review File is licensed under a Creative Commons Attribution 4.0 International License, which permits use, sharing, adaptation, distribution and reproduction in any medium or format, as long as you give appropriate credit to the original author(s) and the source, provide a link to the Creative Commons license, and indicate if changes were made.
In cases where reviewers are anonymous, credit should be given to 'Anonymous Referee' and the source.
The images or other third party material in this Peer Review File are included in the article's Creative Commons license, unless indicated otherwise in a credit line to the material. If material is not included in the article's Creative Commons license and your intended use is not permitted by statutory regulation or exceeds the permitted use, you will need to obtain permission directly from the copyright holder.
To view a copy of this license, visit https://creativecommons.org/licenses/by/4.0/

RESPONSE TO REVIEWERS' COMMENTS
Please consider this letter as a response to each of the comments of the referees. We thank each of them for providing valuable questions, feedback, and insights.

Reviewer(s)' Comments to Author:

Referee 1
Summary:
Carter et al. report the thorough characterization of Tb^{4+} prepared under aqueous conditions supported by the W17 POM. The demonstration that Tb^{4+} can be formed and have sufficient lifetime under aqueous (not non-aerobic) conditions is an important extension of the detailed physical characterization studies reported by La Pierre, Mazzanti, and Zheng of molecular Tb^{4+} complexes prepared in non-aqueous and rigorously O_2/H_2O-free conditions. These characterization studies of TbW34 build from the synthetic methodology reported by Fedoseev and co-workers using persulfate as the oxidant in large excess. While the Tb^{4+} oxidation state is only barely stable under a variety of conditions necessary for characterization the authors have pursued a rigorous, detailed, and convincing demonstration of the Tb^{4+} oxidation state. Furthermore, this 8-coordinate near cubic setting provides deep comparative insight to the physical properties of Tb^{4+} in comparison to 4-coordinate molecular complexes and 8-coordinate cubic oxide settings in oxide materials. Overall, this is an important paper that will be well-cited by the community. It should be published in Nature Communications after the authors consider the following largely presentation issues.

Technical:
1) The reported technical work spanning crystallography, EPR, magnetometry, XAS, and theoretical description are very well executed and well described. While I think the EPR analysis would benefit from high-field/high-frequency analysis (the Gd^{3+} simulation could be stronger), this EPR analysis work would appropriate for a follow-up study.

Our Response: We thank the referee for their positive feedback, and we agree that high-frequency EPR measurements would be of value to further our understanding of these POM systems. These measurements are currently being pursued, and will be the subject of a follow-up study.

Action Taken: None

2) While the reported analysis is quite thorough, I would like to see some electrochemical analysis. Computational analysis suggests that the 4/3 couple lies just within the aqueous window. Is this really the case? Or is the exclusive utility of persulfate as the oxidant due to a kinetic phenomenon? The latter is suggested by the instability of the complex under aerobic conditions. What are the technical challenges for this measurement?

Our Response: We agree that electrochemical analysis would be of interest. It is outside the expertise of any of the authors on this study; nevertheless, we attempted to collect cyclic voltammetry data at both UI and LANL where different electrochemical set ups

were available. In both cases, we were unable to obtain reasonable spectra likely due to a combination of solubility limitations for the Tb(IV) POM system at concentrations required for CV measurements, challenges associated with aqueous electrochemical measurements, and kinetic challenges highlighted by the referee. Accordingly, we have reached out to groups with aqueous electrochemistry expertise to initiate new collaborations, and the results from these efforts will be the subject of a follow-up study as well.

Action Taken: None

Presentation:
1) Valence and oxidation state are not interchangeable terms – the authors are referred to IUPAC definitions. This ambiguity though is the fault of the field of f-element coordination chemistry which has routinely used these terms as synonymous. I would recommend that the authors refer to the oxidation state of the ion and in particular avoid phrasings in which the terms are used together (as in the first line of the paper: trivalent oxidation states – which erodes any clear distinction and/or is repetitive).

Our Response: This is a good point and we thank the referee for highlighting this subtle but important distinction. We have updated the manuscript consistent with the referee's suggestion in each case where we found valence and oxidation state being used interchangeably.

Action Taken: The first sentence of the introduction has been updated to read, "A defining feature of lanthanide molecular chemistry is the persistence of metals in the +3 oxidation states.[1-3]"

The second to last sentence of the first paragraph in the introduction has been updated to read, "In aqueous media, molecular examples of divalent and tetravalent lanthanides, beyond Ce, are rare as lanthanide metal ions are labile toward redox processes that lead back to the +3 oxidation state."

The last sentence of the penultimate paragraph of the introduction has been updated to read, "A shift of the Tb(IV)/(III) redox potential by 1.2 – 1.3 V would bring this value to just below 2 V vs. NHE, and this reduction potential shift allows strong chemical oxidants such as persulfate ($S_2O_8^{2-}$) to oxidize Tb(III) within the electrochemical window of water.[35,39,40]"

The third sentence of the UV-Vis results paragraph has been updated to read, "The La Pierre and Mazzanti groups have reported that their Tb(IV) complexes exhibit LMCT spectral bands that are absent from spectra of Tb(III) analogues, and here we also confirmed the observed spectral feature at 437 nm was unique to Tb(IV)W_{34} and a result of oxygen to terbium ligand-to-metal charge transfer using time-dependent density functional theory calculations (**Figure S5, Supporting Information**)."

The last sentence of the EPR and magnetism paragraph has been updated to read, "The much smaller **D** and **E** values for Gd(III)W_{34} compared to Tb(IV)W_{34} are consistent with previous findings from La Pierre and colleagues,[28] who found that significantly larger ZFS values for Tb(IV) are the result of an increase in spin orbit coupling going from Gd(III) to Tb(IV), and our results confirm this trend applies for W_{34} complexes."

2) In the introduction the authors choose the non-alphabetical listing "Mazzanti and La Pierre" and provide two blocks of references for these authors in that order. This seems to make some non-scientific point that is not germane to the authors' paper.
a. I suggest the authors use the alphabetical order as they largely do throughout the rest of the paper and then group the references by topic: citations for Tb4+ (which are then in year of publication) and for Pr4+ (which are then in year of publication)

Our Response: We thank the referee for the suggestion and we have updated the manuscript accordingly.

Action Taken: We have updated the relevant sentence in the introduction to read, "This paradigm changed with the recent works from the groups of La Pierre, Mazzanti and Zheng, which demonstrated that isolation and characterization of molecular complexes of tetravalent praseodymium (Pr) and terbium (Tb) was possible in non-aqueous, inert conditions.[18-30]" References have been re-ordered based on topic (Tb(IV) papers first, followed by Pr(IV) papers), and additional references have been added based on referee suggestions included below.

b. The authors should include initial report of Pr4+ molecular complex by the La Pierre group (Dalton Trans., 2022, 51, 6696-6706) in the references.

Our Response: We thank the referee for the suggestion and we have referenced this paper in the updated version of the manuscript.

Action Taken: The highlighted paper is now cited in the manuscript (it is ref. 26 in the updated reference list).

c. The authors may also consider noting the extension of this approach for the characterization of a 5+ praseodymium complex: Nature Chemistry volume 17, pages 1005–1010 (2025)

Our Response: This is a wonderfully interesting recent paper, and we considered the referee's suggestion about citing it as well. However, we could not figure out a way to cite it without introducing additional details and context that distracted from the focus of the introduction. As a result, we did not include this reference in the updated manuscript.

Action Taken: None

3) The work Zheng and co-workers is elided from the discussion except for one reference grouped with the studies of Mazzanti.

a. The authors are encouraged to include this groups synthetic and physical characterization studies of Pr4+ and Tb4+ complexes:

i. Dalton Trans., 2024,53, 5779-5783
ii. Precis. Chem. 2023, 1, 10, 583–591 (included)
iii. Chem. Sci., 2025,16, 6805-6811

Our Response: We thank the referee for the suggestion and we have highlighted the work of Zheng in the introduction and added citations we previously overlooked.

Action Taken: We have updated the relevant sentence in the introduction to read, "This paradigm changed with the recent works from the groups of La Pierre, Mazzanti and Zheng, which demonstrated that isolation and characterization of molecular complexes of tetravalent praseodymium (Pr) and terbium (Tb) was possible in non-aqueous, inert conditions.[18-30]" Moreover, 2024 and 2025 papers from Zheng *et al*. have been cited in the updated manuscript (these are refs. 24 and 29 in the updated reference list).

4) The authors describe, "The strategy developed by the groups of Mazzanti and La Pierre for accessing tetravalent Tb and Pr is based on the use of bulky ligands with strong σ and π donating sites that can destabilize the lanthanide f-orbitals, thereby lowering the energetic favorability of the trivalent configuration. The weak field ligands used by both groups also have the overall effect of increasing ligand covalent character such that tetravalent congeners are stabilized, and the thermodynamic cost of oxidation is diminished."
a. Yes both systems developed by La Pierre and Mazzanti fit this hypothesis, however only La Pierre has articulated this hypothesis and design strategy. The phrasing of this statement should be revised.

Our Response: We thank the referee for this detailed insight, and have updated the relevant sentence in the introduction.

Action Taken: The sentence highlighted by the referee has been updated to read, "The current strategy for accessing tetravalent Tb and Pr, which has been explicitly outlined by the group of La Pierre and used by the groups of Mazzanti and Zheng, is based on the use of bulky ligands with strong σ and π donating sites that can destabilize the lanthanide *f*-orbitals, thereby lowering the energetic favorability of the trivalent configuration."

Referee 2
Summary:
The paper presented by Carter and co-workers reports the synthesis and the single crystal structure of a K16Tb(IV)(P2W17O61)2•39.85H2O (Tb(IV)W34) isolated from water and extensive spectroscopic evidence obtained via UV-Vis-NIR, X-ray absorption near edge spectroscopy, EPR spectroscopies and SQUID magnetometry measurements to confirm the presence of Tb in the oxidation state +4.
The work reported revisit some solution studies described in two papers by V. P. Shilov, and A. M. Fedoseev (Radiochemistry 2007 and Russian Journal of General Chemistry, 2022) where the oxidation of Tb(III) by persulfate ions in solutions of unsaturated phosphotungstate K10P2W17O61 was studied by spectrophotometry. Fedoseev and Shilov

also investigated stability in water and its dependency from pH by potentiometry. In the present work Carter and coworker using analogue conditions were able to isolate and fully characterize in the solid state the Tb(IV) complex. The introduction is appropriate and well referenced. The discussion of the various analysis performed is almost always clear and informative and the authors present a good case for the definitive isolation of aqueous stable Tb(IV) species and the nature of its oxidation state. The novelty of the present contribution lies in the isolation of the Tb(IV) complex in appreciable quantities, its XRD characterization and in the subsequent investigation of the lanthanide oxidation state. This is an important achievement. However, especially considering that the formation of this complex had already been reported, the manuscript needs to report more rigorously the synthetic method used and investigate more in detail the oxidation conditions and the stability of the final complex.
The presentation of the synthetic part of the work lacks the rigor required for publication in a high-level journal. There are technical aspects of the synthetic part that need to be resolved before publication of this manuscript can be published.
In particular, the nature and purity of the Tb(IV) complex used for all the measurements needs to be specified at all time and the pH measured and reported for all measurements carried in solution and for the stability studies.
From reading the manuscript is not clear how the Tb(IV) can be isolated analytically pure and if it can exist in solution in the absence of the oxidizing agent. The stability study is also very important but unconvincing as described. Previous work reported that reduction to Tb(III) by water occurred depending on pH. This is ignored by the authors and instability seems to be assigned to reaction with oxygen?
Below are indicated the specific points that should be addressed before publication in any high-level journal.

Our Response: We thank the referee for their detailed and constructive feedback. We have repeated experimental procedures so specific pH information could be obtained, and this is now included in the revised manuscript. Additional experiments were also conducted to address the referee's purity related questions, and while these could not be unambiguously resolved, we have provided additional results and context both in this letter and in the resubmitted manuscript and supporting information. We appreciate the referee's question as well related to the role of pH in the Tb reduction process. We did not ignore the previous studies that mentioned this relationship, rather this was not something we emphasized previously or in the revised submission, since results we have obtained do not suggest pH plays a significant role in affecting the Tb oxidation state.

Action Taken: No specific changes were made based on these broader comments. Revisions to the manuscript based on specific suggestions from the referee are addressed in the points below.

Comments:
i) Concerning the synthesis of K16/17Ln(III/IV)(P2W17O61) xH2O: In the reported procedure by Peacock and Weakley (1971 J. Chem. Soc. A. 1836) I could only find the Ce(III)W34 and Ce(IV)W34 complexes. In light of this, characterization method to assess purity and water content of the isolated crystals (e.g.: TGA for H2O content and ICP for

W) should be used for the complexes that were not reported previously. The large amount of water on the isolated bulk compound is especially important for the magnetic studies.

Our Response: There have been a number of additional studies on Ln(III) and Ce(IV) POM complexes with the lacunary Wells-Dawson anion since the initial report by Peacock Weakley (see for ex. Inorg. Chem. 2001, 40, 1894-1901; J. Cluster Sci. 2003, 14, 431-446; Inorg. Chem. 2006, 45, 2055-2060; J. Mol. Struct. 2013, 1040, 33-38). Across these studies, and many others, there is not a standardized set of measurements used to assess POM analytical purity, and variations in data provided are likely driven by instrumentation available to different research groups. Here the question of water content is a good one, and we pursued TGA and elemental analysis measurements to gain further insights. ICP data is not something we are equipped to readily obtain, and it is unclear what information this measurement would provide that is not already known from the SCXRD structure.

Action Taken: Water content in freshly prepared crystals can be obtained from SCXRD results, and this information is included in the formula provided for the Tb(IV)W_{34} POM in the abstract of the manuscript, $K_{16}Tb(IV)(P_2W_{17}O_{61})_2{\bullet}39.85H_2O$. The TGA instrument available at the University of Iowa is an older model, and weight loss events less than 5% are considered within measurement error for the instrument. 39.85 water molecules represents 7.3% of the overall mass of the Tb(IV) POM (based on the formula provided above), and TGA data collection on two freshly prepared batches of Tb(IV)W_{34} crystals did not provide quantitative information on water content. We also pursued C, H, N elemental analysis data on freshly prepared Tb(IV)W_{34} crystals, which yielded H percentages of 0.49% and 0.44%. Hydrogen accounts for 0.8% of the formula weight of Tb(IV)W_{34} based on the SCXRD formula, and these values are consistent based on +/- 0.4% relative error values that are deemed acceptable for EA results (see Table 1 in ACS Central Sci. 2022, 8, 855-863 for more information).

ii) The synthesis of a general complex [Tb(IV)(P2W17O61)2]16- is given in the method while in the main text the formula of the complex is never specified and one has to wait to get to Sample Preparation for Tb L3-edge XAS Measurements to find the formula of the complex (K16[Tb(IV)(P2W17O61)2]·xH2O). However, there is no information on how the formula was established and on how many water molecules are present in the complex. The large amount of water on the isolated bulk compound is important especially for the magnetic studies. The author should use TGA to determine it. Purity of the complex should also generally be established by analytical techniques.
The formula obtained from the X-ray diffraction is also not presented. These issues should be solved, and presentation of the complex should be more rigorous.

Our Response: We must respectfully push back against some of the information provided in this comment. The complete formula for Tb(IV)W_{34} is provided in the final sentence of the abstract, "Herein we present the single crystal structure of $K_{16}Tb(IV)(P_2W_{17}O_{61})_2{\bullet}39.85H_2O$ (Tb(IV)W_{34}) as well as extensive spectroscopic evidence obtained via UV-Vis-NIR, X-ray absorption near edge spectroscopy, continuous wave X-band electron paramagnetic resonance spectroscopies and SQUID magnetometry measurements to confirm the oxidation state of Tb in W_{34} complexes as +4." It is also

included in the final sentence of the first paragraph of the introduction, "We have overcome this limitation using polyoxometalate (POM) chemistry, and herein we present, $K_{16}Tb(IV)(\alpha_2\text{-}P_2W_{17}O_{61})_2 \bullet 39.85H_2O$ ($Tb(IV)W_{34}$), a molecular tetravalent terbium complex that can be synthesized and characterized in water, although still susceptible to auto reduction when exposed to air." The point about the formula included in the methods section for $Tb(IV)W_{34}$ is a good one and we have updated the manuscript text accordingly. The extent of hydration around POM complexes is known to be dynamic and is impacted by a number of factors; thus, in instances where real-time information on POM complex water content could not be obtained, such as samples prepped at the beamline ahead of XAS measurements, we included complex formulas without a specific number of water molecules. We investigated the water content in the POM crystals using TGA and EA measurements (see the point above in this letter) and we agree as well that water content is important for magnetic measurements, and provide more specifics related to this point below (where this is a comment specifically related to SQUID magnetometry results).

Action Taken: The formula for the $Tb(IV)W_{34}$ complex included in the methods section has been updated to read, "Synthesis of $K_{16}[Tb(IV)(P_2W_{17}O_{61})_2] \bullet xH_2O$."

Concerning the synthesis I have several key concerns tht should be addressed in full before publication.
a) When conducting synthesis and reactivity in water it is essential to report pH values as they can be very important in determining the reaction outcome and in determining stability. The authors should also investigate the effect of pH on stability. This is a key point and should be addressed for publication. Several authors of this manuscript have long-term experience in working in water and are aware of these problems/requirements.

Our Response: We agree and thank the referee for the suggestion. The pH of the lacunary POM (W_{17}) solution used for synthesizing the trivalent Wells-Dawson complexes is approximately 6.5. Addition of Ln(III) aliquots, following the procedure included in the manuscript, does not affect the reaction solution pH. After the addition of 100 eq. of $K_2S_2O_8$ (relative to Tb(III)), which is part of the procedure for making $Tb(IV)W_{34}$, the pH of the reaction solution is reduced to ~2.5, and this value is consistent with the findings of Shilov and Fedoseev in their respective studies.

Action Taken: Specific pH information was added to the synthesis section of the manuscript, and the following sentences were updated.

In the Synthesis of $K_{16/17}Ln(III/IV)(P_2W_{17}O_{61})_2 \bullet xH_2O$ (LnW_{34}) where Ln = Ce(III/IV), Gd(III), Tb(III), Dy(III) section, the text now reads, "The synthesis protocol used to prepare LnW_{34} complexes is based on the procedure first outlined by Peacock and Weakley.[65] 0.25 grams of $K_{10}P_2W_{17}O_{61} \bullet 20H_2O$ (0.05 mmol) was dissolved in 9.75 mL Milli-Q H_2O with heating (90 °C) and stirring (pH = ~6.5). Subsequently, 250 µL of 0.1 M $Ln(NO_3) \bullet xH_2O$ / $LnCl_3 \bullet xH_2O$ / $(NH_4)_2Ce(NO_3)_6$ (0.025 mmol) solution was added in 50 µL portions into the heated (90 °C) polyoxotungstate solutions, and pH values stayed consistent at approximately 6.5."

In the Synthesis of $K_{16}[Tb(IV)(P_2W_{17}O_{61})_2]•xH_2O$ section, the text now reads, "To prepare $Tb(IV)W_{34}$, 100 molar eq of $K_2S_2O_8$ was added into a solution containing 1 eq of $Tb(III)W_{34}$ solution, prepared using the procedure outlined above, with stirring at 80 °C, which lowers the reaction solution pH to ~2.5."

Another important question is why oxidation is performed with large amounts of K2S2O8(100 equiv) ? The authors should report what happens with lower amounts of oxidizing agent.

Our Response: 100 molar equivalents of $K_2S_2O_8$ were used as this was the quantity we found consistently yielded the desired $Tb(IV)W_{34}$ product. The referee's question about what happens with lower amounts of oxidizing agent is an interesting one that is outside the scope of this manuscript, and one that we plan to probe in a follow-up study. Worthy of additional comment, the 100 molar equivalents we used here to oxidize Tb is a significant decrease compared to the studies from Shilov and Fedoseev where 2500-7500 equivalents of $S_2O_8^{2-}$ were used to generate Tb(IV).

Action Taken: None

The authors state: "After one day, colorless and brown crystals were observed. The colorless crystals were unreacted K2S2O8, while the brown crystals are Tb(IV)W34¬ (0.382 g, 39% yield)." How can a yield be established when the pure complex cannot be separated from unreacted K2S2O8? Have the authors obtained the complex analytically pure? This is very important.

Our Response: This is a good question and during the process of preparing the manuscript for submission this is one we thought about a lot. Due to the persistence of excess persulfate in $Tb(IV)W_{34}$ samples, traditional methods for obtaining reaction yields were not possible. In our methods section, we aimed to provide complete details for each reaction, and so we designed a semi-quantitative process for determining the yield of the $Tb(IV)W_{34}$ reaction based on data obtained from multiple measurements. This involved preparing fresh $Tb(IV)W_{34}$ crystals, and then harvesting them, along with excess persulfate. The total weight of the mixture of products was determined and then the crystalline sample was ground thoroughly. Subsequently, we conducted energy dispersive X-ray spectroscopy on the $Tb(IV)W_{34}$ sample to obtain the elemental ratios between sulfur and tungsten as well as sulfur and terbium. Based on these ratios, we can obtain a semi-quantitative yield of $Tb(IV)W_{34}$ from the mixture of products. From there, we accounted for the amount of $Tb(IV)W_{34}$ within the bulk crystalline product based on our SCXRD results. Tb-O bond distances in $Tb(IV)W_{34}$ indicate that in the solid-state around 70% of the Tb within the crystals are in the +4 oxidation state, which provides us a conservative estimate of the amount of $Tb(IV)W_{34}$ produced by each synthesis.

Action Taken: We have updated the relevant text in the methods section of the manuscript to read, "The colorless crystals were unreacted $K_2S_2O_8$, while the brown crystals are $Tb(IV)W_{34}$ (54% yield (semi-quantitative)). The brown crystals were stored in the fridge (5 ºC) in a capped container filled with the mother liquor to slow down the auto-reduction

process back to Tb(III)W_{34}. Worthy of additional comment, obtaining a yield value for Tb(IV)W_{34} is challenging due to co-crystallization with $K_2S_2O_8$. Gravimetric analysis and scanning electron microscopy-energy dispersive spectroscopy (SEM-EDS) were used to obtain the semi-quantitative yield value provided above. Details regarding this yield calculation can be found in the **Supporting Information**." Additionally, SEM-EDS results and yield calculation information have been added to the supporting information section where they can be found in **Figure S11**, **Table S6**, and in the paragraph on **pg. S26** titled, "**Tb(IV)W_{34} Yield Calculation Details**."

If is not the case, a separation should be pursued.

Our Response: We have spent a lot of time over the past month and a half thinking about this comment and pursuing potential separations pathways. A significant excess of $K_2S_2O_8$ is required for Tb oxidation and some of the oxidant crystallizes with the brown crystals of the Tb(IV) POM. In ambient conditions, debulking $K_2S_2O_8$ requires prolonged air exposure, which leads to Tb reduction back to the +3 oxidation state. The POM complex and $S_2O_8^{2-}$ are both anions, and they have different affinities for anion exchange resins as a result of their differences in charge. However, during the course of running a range of anion exchange columns, which in our lab involves extended exposure to ambient conditions, Tb was once again reduced back to the +3 oxidation state in all cases. Overall, this is a worthwhile line of investigation and we thank the referee for the suggestion. Based on what we have learned from attempting this separation during the course of the revisions process, isolating Tb(IV) in analytically pure form will require further optimization in terms of its coordination and solution stability with POM anions before this can be achieved, which is beyond the scope of this study.

Action Taken: None

The authors also need to clearly specify for every following experiments if the measurements were carried out on pure complex or on a mixture of Tb(IV) and oxidizing agent.

Our Response: We agree that providing specific information on samples used for data collection is important, and we direct the referee to the following locations in the Methods section where this information was included for the Tb(IV)W_{34} complex.

For SCXRD – "Crystals of Tb(IV)W_{34} are air sensitive and, after a period of approximately ten minutes, lose their brown color indicating the reduction of Tb(IV) back to Tb(III). Tb(IV)W_{34} crystals also quickly dissolve back into the mother liquor, in under five minutes, at room temperature. To address these challenges, mounting of the crystals was done on a makeshift stage with an ice pack to keep the temperature low and the crystal of Tb(IV)W_{34} was mounted in the liquid N_2 cold stream in under ten minutes."

For XAS – "The $K_{16}[Tb(IV)(P_2W_{17}O_{61})_2]\cdot xH_2O$ solution was prepared in a fume hood by boiling a mixture of $K_{17}[Tb(III)(P_2W_{17}O_{61})_2]\cdot xH_2O$ (80 mg, 8.1 mmol) with $(NH_4)_2S_2O_8$ (200 mg, 0.88 mmol) in H_2O (1 mL) in a glass vial (3 mL). An aliquot of the resulting

orange-brown solution (70 μL) was then transferred into a sample holder that had been 3D-printed from VeroClear (Stratasys). This holder was best described as an open box whose inner dimensions were 18 mm (height) × 2 mm (width) × 2 mm (depth) with a total volume of approximately 0.07 mL. The open side of the sample holder was sealed with Kapton tape (0.5 mil; 12.7 μm). Solutions were pipetted into the holder through a hole that had been bored through one end of the box. This injection hole was subsequently sealed with Kapton tape and the sample was placed in the holder attached to a rod and flash frozen in a bath of liquid nitrogen. The frozen sample was transferred to the end-station hutch and rapidly inserted into the cryostat against a strong flow of helium."

For EPR – "Samples of Tb(IV)W_{34} analyzed using EPR spectroscopy were prepared by slow evaporation from a 5 mM solution and crystals were harvested with a transfer pipette along with the mother liquor."

For SQUID Magnetometry – "For Tb(IV)W_{34}, samples were weighed into the VSM powder sample container in the form of crystals (each was hand-picked and loaded) and subsequently turned into a powder within the sample holder."

Action Taken: As part of addressing this comment from the referee we noted that there was opportunity to add clarity to the UV-Vis-NIR data collection details, and accordingly we have updated the manuscript text to read, "The UV-Vis-NIR spectra of Tb(IV)W_{34}, Tb(III)W_{34}, Ce(IV)W_{34}, Ce(III)W_{34}, Gd(III)W_{34}, and Dy(III)W_{34} were collected from freshly prepared 0.25 mM solutions of each POM."

When redissolving the pure? solid in water (if this is what is done) the pH of the obtained solution needs to be measured and indicated.

Our Response: When Tb(IV)W_{34} crystals, as well as excess persulfate, are dissolved in water the pH is approximately 2.6 (based on measurements with an Ohaus Aquasearcher pH meter). We measured these values in capped and uncapped vials, mimicking those used in UV-Vis-NIR stability studies, and these values stay consistent at around 2.6 over the course of two weeks.

Action Taken: We added the following sentence to the UV-Vis-NIR section of the methods that provides pH specific information about Tb(IV)W_{34} crystals upon dissolution in water, "Sample pH was monitored during the duration of measurements and stayed consistent for both capped and uncapped solutions at approximately 2.6."

b) I am very concerned about the stability studies.
The authors should specify precisely how they were conducted. How was the sample initially prepared, in glove box or in air?

Our Response: We direct the referee to the following sentence in the Methods section where we provided information on how samples for the stability study were prepared, "The longitudinal stability study was conducted on two samples of Tb(IV)W_{34}, each at a concentration of 0.25 mM, with one of the samples capped and the other one left uncapped

with data collected regularly over twelve days." Moreover, additional details on different stability study measurements are included in the UV-Vis-NIR paragraph of the manuscript in the following sentences, "Moreover, the Tb(IV) metal center in Tb(IV)W_{34} was found to be stable in a closed aqueous system as demonstrated by longitudinal UV-Vis-NIR measurements wherein the molar absorptivity of the LMCT band (A_{437}) was monitored, **Figures 2C** and **2D**. Upon transferring the Tb(IV)W_{34} solution from a cuvette to container with greater surface area such as a plastic petri dish, while maintaining the same volume, the A_{437} decreases exponentially, **Figure 2C**. Extending measurements on Tb(IV)W_{34}, kept under closed aqueous conditions, revealed the sustained presence of the 437 nm charge transfer peak over twelve days, **Figure 2D**." Overall, all samples were prepared in ambient conditions in a chemical fume hood. In between measurements, capped and uncapped sample vials were kept under ambient conditions in secondary containment on a lab bench.

Action Taken: To further address the referee's concerns, we conducted additional control experiments. Samples of $TbCl_3$ with and without excess persulfate, and matching the conditions and ionic strength of Tb(IV)W_{34} samples, were monitored using UV-Vis-NIR spectroscopy and no peak at 437 nm was observed. This indicated that lacunary Wells-Dawson POM ligands are required to access and maintain spectroscopic signals characteristic of Tb(IV) under aqueous conditions.

When stating: The brown crystals were stored in the fridge (5 ºC) in a capped container filled with the mother liquor to slow down the auto-reduction process back to Tb(III)W34. What capped container means? Under inert atmosphere? The authors seem to hint that air is reducing Tb(IV) to Tb(III) and not water as was mentioned in the original work of Fedoseev . It is not clear what they propose, are they proposing that oxygen reduces Tb(IV) ? but water does not? This seems unlikely. Or what else?

Our Response: Capped container in this context refers to a standard 20 mL scintillation vial. As mentioned above (and in the manuscript), all synthesis and characterization studies were done in ambient environments. We consistently observed that the brown crystals of Tb(IV)W_{34} were air sensitive, and would quickly change color, indicative of Tb reduction. Accordingly, sample preparation procedures for different measurements were designed and optimized so that Tb(IV)W_{34} crystal exposure to ambient conditions outside the sample mother liquor were minimized. We also observed a correlation between air exposure and Tb reduction in our UV-Vis-NIR stability study, and we agree that there is likely more to the reduction process. Water is known to act as a reductant when paired with strong oxidizers. Ce(IV) is a common strong oxidizing agent and Tb(IV) likely is too. Our current hypothesis is that in the sample mother liquor, the excess persulfate acts as a stabilizer, similar to those added to reactive species used in synthesis, such as H_2O_2. Upon removing crystals from the mother liquor, exposure to air, and the water contained within it, leads to Tb reduction, which is consistent with the takeaways from the work of Fedoseev.

Action Taken: None

Have the authors checked the variation of pH over time in the capped and uncapped vials. Fedoseev and coworkers had clearly indicated a pH dependency of the stability.

The stability is an important aspect here and needs to be studied and presented rigorously before publication.

Our Response: This is a good question and we investigated how pH changed over time in capped and uncapped vials as a part of putting together the revised manuscript.

Action Taken: As noted above in response to an earlier point, we added a sentence to the UV-Vis-NIR paragraph in the methods section that reads, "Sample pH was monitored during the duration of measurements and stayed consistent for both capped and uncapped solutions at approximately 2.6." We also added a new table to the supporting information with details on pH values obtained at different timepoints, and these details can be found in **Table S5**.

Other points
i) In the Materials section, the last sentence is dedicated to the synthesis of the potassium POM salts and K6P2W18O62 is referenced. However, I do not see why, since such salt does not seem to be used in the paper.

Our Response: This reference is included because preparing $K_{10}P_2W_{17}O_{61}$•$20H_2O$ for use in reactions with lanthanide cations is a two-step process that first involves preparing $K_6P_2W_{18}O_{62}$•nH_2O.

Action Taken: None

ii)The magnetic measurement provided are in nice agreement with Tb(III) and Tb(IV) species. I appreciate the detailed description of the method used to obtain the theoretic magnetic moments and the SQUID sequences used. However, given such a thorough report, I am surprised that, if diamagnetic corrections were applied, such values are not reported. On the same line, I wonder how many molecules of H2O the authors assume to be present per metal center and on what basis do they assume that.

Our Response: We thank the referee for the good question. As we have highlighted in earlier points, the number of lattice water molecules in Tb(III) and Tb(IV)W_{34} structures can be initially obtained from SCXRD data, and evolves over time. As part of the resubmission, we conducted TGA and EA measurements to gain more insights about lattice water content and how it may change over time. Unfortunately, this data did not provide additional quantitative information, so we conducted a series of magnetometry data fits where we systematically changed the diamagnetic correction to reflect formula weights that included a range of water molecules (zero to double the number we observed via SCXRD (80)). Based on the number of water molecules included in SCXRD structures, 42.5 for Tb(III)W_{34} and 39.85 for Tb(IV)W_{34}, we obtained, and reported in the manuscript, μ_{eff} values for Tb(IV)W_{34} and Tb(III)W_{34} are 8.76 μ_B and 10.15 μ_B. When the total number water molecules is varied between 0 and 80, the μ_{eff} range for Tb(III)W_{34} is 9.71 – 10.57 μ_B and for Tb(IV)W_{34} it is 8.05 – 9.47 μ_B. In the manuscript we stated, "Effective magnetic moment values for both complexes are broadly consistent with expectations for Tb(IV) and

Tb(III) complexes," and based on this additional analysis it is our assessment that this takeaway is still valid.

Action Taken: To provide further insights and to address the referee's question, we conducted additional analysis of SQUID magnetometry data collected on Tb(III)W_{34} and Tb(IV)W_{34} complexes to assess how diamagnetic correction values could impact effective magnetic moments. Overall, it was found that even as diamagnetic correction values changed as a function of changing the number of lattice water molecules, takeaways from magnetometry data stayed consistent and Tb(III)W_{34} and Tb(IV)W_{34} displayed distinct χT versus T behavior.

Moreover the authors state: Effective magnetic moment values for both complexes are broadly consistent with expectations for Tb(IV) and Tb(III) complexes, with the possibility of a small amount of reduction of Tb(IV)W34 occurring during the sample loading process ahead of measurement."
This is ambiguous and it would be interesting to know how this is assessed. Without presenting data for a pure complex the story remains ambiguous. Are the authors sure that the Tb(III) is not always presend?

Our Response: There is certainly a possibility that some percentage of Tb(III) is present. This is very challenging to analytically verify while collecting other types of data, such as SQUID magnetometry, and ultimately the precise quantity of Tb(III) present in a Tb(IV)W_{34} sample does not change the takeaways we have included in the manuscript. Rather than speculate, we aimed to be honest and to clearly represent the uncertainty that is inherent in experimental data. The χT versus T plot in Figure 4B highlights a qualitative difference between the Tb(III)W_{34} and Tb(IV)W_{34} curves, with the latter exhibiting character consistent with other Tb(IV) examples in the literature (see La Pierre *et al*. JACS, 2019, 141, 13222-13233). Moreover, as addressed in the point above, even with potential varying amounts of water in the lattice, that can affect diamagnetic correction values, effective magnetic moment values are clearly different, and values for Tb(IV)W_{34} are most consistent with expectations for a metal-ion with a $4f^7$ electron configuration.

Action Taken: None

Was Gd(III) measured for comparison?

Our Response: Due to limited SQUID measurement time, the Gd(III)W_{34} POM complex was not analyzed using this technique. As we had already been able to collect X-band EPR data, which provided more accurate information on how ZFS values were changing, we decided that the most appropriate comparison for magnetic measurements was Tb(III)W_{34} vs. Tb(IV)W_{34}.

Action Taken: None

iii) I am slightly confused on the necessity to prove that the LMCT band observed at 437 nm is not due to 'unaccounted interactions between K2S2O8 and Ln(III)W34 complexes'.

Is the UV-Vis-NIR recorded on a pure sample of Tb(IV)W34 or on a reaction mixture K2S2O8? If the latter is the case, was the stability test performed on Tb(IV)W34 performed in presence or absence of excess K2S2O8? Was the pH evolution over time measured? Stability tests in the presence of K2S2O8 would have little meaning and to evaluate stability in water the test should be done on the pure complex. The effect of pH should also be carefully monitored .
It remains ambiguous if a pure Tb(IV) complex can be obtained and carefully characterized

Our Response: We aimed to understand the persistence of Tb(IV)W_{34} complexes in solution as comprehensively as possible, and it was this goal that led us to investigate whether unaccounted interactions between $K_2S_2O_8$ and Ln(III)W_{34} complexes were the origin of the UV-Vis-NIR peak observed at 437 nm. Spectra for Tb(IV)W_{34} were collected in the presence of excess $K_2S_2O_8$. Stability study data was also collected with excess persulfate present within the solution, and in our view these results are notable and relevant since the same spectral results cannot be achieved with only a mixture of Tb(III) and $K_2S_2O_8$ (where the oxidation reaction has not been performed). This indicates that the W_{17} POM ligands are helping to stabilize Tb(IV), which is consistent with findings of Fedoseev and colleagues (see refs. 35 and 40 in the updated manuscript) as we mentioned in the original version of the manuscript. As part of the revisions process, we have monitored the pH of stability study solutions and this data is included in **Table S5** of the updated **Supporting Information** document.

Action Taken: Control experiments were run to understand if UV-Vis-NIR spectra for solutions containing Tb(III) and excess persulfate, where the oxidation reaction was not performed ahead of measurement, featured a charge transfer peak at 437 nm that is characteristic of Tb(IV). Additionally, pH values of stability study solutions were monitored and this data is included in **Table S5**, **Supporting Information**.

Why in the Sample Preparation for Tb L3-edge XAS measurements the K16[Tb(IV)(P2W17O61)2]·xH2O solution was prepared using (NH4)2S2O8 rather than K2S2O8? Please also here indicate pH values for the soliutions or reproducibility cannot be ensured.

Our Response: Ammonium persulfate was used to prepare samples for XAS measurements due to the greater solubility of this species, which was advantageous for data collection purposes. We have also added sample pH information to the **Sample Preparation for Tb *L_3*-edge XAS Measurements** section of the manuscript.

Action Taken: The sentence where Tb(IV)W_{34} XAS sample preparation is described in the methods section has been updated to read, "The $K_{16}[Tb(IV)(P_2W_{17}O_{61})_2]\cdot xH_2O$ solution was prepared in a fume hood by boiling a mixture of $K_{17}[Tb(III)(P_2W_{17}O_{61})_2]\cdot xH_2O$ (80 mg, 8.1 µmol) with $(NH_4)_2S_2O_8$ (200 mg, 0.88 µmol) in H_2O (1 mL) in a glass vial (3 mL) where the solution pH was approximately 2.6."

For the following statement 'Fedoseev and colleagues have been the only group to pursue this chemistry further;37,38 however, beyond ...' ref. 38 is Hoefdraad H. E. 1975 J. Inorg. Nucl. Chem. is not relevant to the statement, however, ref. 32 is (Fedoseev A. M. Radiochemistry 2007).

Our Response: This is a good point and we have updated the manuscript accordingly.

Action Taken: In the introduction sentence highlighted by the referee, we have updated the citations to include the 2007 work from Fedoseev and colleagues and removed the reference from Hoefdraad. Of note, the reference numbers here are different due to additional citations added at the request of referee #1. The 2007 Fedoseev paper that the referee has highlighted is reference 35, while the original ref. 37 is now reference 40.

The updated text in the introduction now reads, "Fedoseev and colleagues have been the only group to pursue this chemistry further;[35,40] however, beyond the identification of a metal-based charge transfer band at 440 nm in UV-Vis-NIR spectra, no further characterization of tetravalent terbium in a TbW_{34} complex has been published."

Finally, I would recommend this manuscript for publication since it significantly advances our understanding of high-valent lanthanide chemistry in aqueous conditions. Nevertheless, the absence of a clear assessment of the chemical purity of the isolated and analyzed samples raises important questions that should be addressed to strengthen the quality of the study. The synthetic conditions should be clearly described with more details including additional studies (evaluating the effect of different amounts of oxidizing agent on the reaction outcome). The pH should be indicated for all experiments. The stability studies should be carefully carried on the pure compound out and carefully described. The authors should isolate the complexes in anaerobic conditions first and then expose them to air controlling the pH evolution.

Our Response: We thank the referee for their overall conclusion and we have addressed each of the points included in their review.

Action Taken: None here, and see earlier points in this letter where we made updates and provided additional details related to POM complex synthesis procedures. On a final note, the referee's suggestion to isolate the complexes in anaerobic conditions and then expose them to air controlling the pH evolution is interesting, and one we will work to pursue in the future.

Referee 3

Summary:

Subintoro et al. present a water-synthesised and more-or-less stable Tb(IV)POM complex. I like the idea and the outcome is reasonably convincing. But, there are some specific pieces of data that do not yet fully convince me. See below. If these can be addressed, and done so in a robust and thorough manner, I would be happy to see this published here.

As a general comment, I like the straight forward presentation of the work, but I dislike the

results summary at the end of the introduction - the paper is not really long enough to need this, and I got confused reading it. Just do the standard "herein we use XYZ techniques to show ABC", and then get into the guts, please.

Our Response: We appreciate the feedback and have edited the final paragraph of the introduction to improve the clarity and focus of the text.

Action Taken: Multiple sentences were either removed and/or shortened and the updated text for the final paragraph of the introduction now reads:

"Herein, we report the synthesis, structure, and spectroscopic characterization of a Tb(IV)W_{34} POM complex. The species was prepared using a two-step process: first, a Tb(III) sandwich complex coordinated by W_{17} ligands was synthesized; second, this precursor was subjected to a post-synthetic oxidation reaction, which converted Tb(III) to Tb(IV), yielding the desired product. The characteristic brown color of Tb(IV) is observed for Tb(IV)W_{34} complexes in both solution and single crystal samples, and this species was characterized in detail using single crystal X-ray diffraction (SCXRD), X-ray absorption (XAS), UV-Vis-NIR, and electron paramagnetic resonance (EPR) spectroscopies as well as magnetometry measurements and density functional theory calculations. SCXRD experiments revealed Tb(III) and Tb(IV) species were isostructural, and comparison of average bond distances between coordinating oxygen atoms (d_{Ln-O}) in the Tb(III) and Tb(IV)W_{34} complexes revealed a ~0.1 Å decrease for the tetravalent complex. X-ray absorption near edge spectroscopy (XANES) measurements on Tb(IV)W_{34} featured a white line doublet that is characteristic of tetravalent terbium; however, the sample is unstable in the X-ray beam and auto-reduction back to Tb(III) was observed. UV-Vis-NIR spectra for Tb(IV)W_{34} feature a ligand to metal charge transfer (LMCT) band with a λ_{max} = 437 nm that is absent in the spectrum of the Tb(III)W_{34} complex. Longitudinal UV-Vis-NIR measurements on Tb(IV)W_{34} were conducted over a period of twelve days, and during the experimental observation period Tb(IV)W_{34} was stable when kept under closed aqueous conditions based on the sustained presence of the 437 nm charge transfer peak.[40] X-band EPR measurements on solid-state samples of Tb(IV)W_{34} further confirmed the Tb(IV) oxidation state assignment as spectra contained features that have only been observed for previously reported molecular Tb(IV) complexes.[31] Zero field cooled SQUID magnetometry measurements for Tb(IV)W_{34} revealed an effective magnetic moment (μ_{eff}) value that is consistent with expectations for a f^7, $^8S_{7/2}$ metal-ion, which also notably contrasted with the magnetic behavior and μ_{eff} value for Tb(III)W_{34}. Finally, density functional theory calculations for Tb(IV)W_{34} found that oxidation happens on the terbium center without involvement of the W_{17} ligands, which also leads to oxygen to terbium ligand-to-metal charge transfer in computed absorption spectra, consistent with experimental findings regarding the tetravalent character of Tb(IV)W_{34}."

Equation 1 has g with a double-headed arrow over the top - this is usually reserved for its role as a matrix in between the magnetic field and spin vectors. Here, however, it is a scalar so should not have the arrow above. Likewise, the B and S should be given as the dot product.

Our Response: These are good points and we have updated manuscript accordingly.

Action Taken: Equation 1 has been updated to incorporate the referee's suggestions.

A little more time needs to be devoted to the EPR simulation section. When D >> hv as here, the exact value of D is poorly captured by simulation, especially given the level of reproduction here. Indeed, some quick playing around indicates that I can get (i) a better simulation by using a significant D-strain (ca. 0.4 cm-1) that broadens the higher-field satellites at ca 250 mT and (ii) that D anywhere between 0.5 and 0.7 gives a reasonable representation of the experiment (all better than the current simulations).

Our Response: We thank the referee for the valuable suggestion; however, there are challenges in accurately accounting for D-strain without high frequency and high field EPR data. Accordingly, we used a simple model for our X-band EPR fit that was consistent with our magnetometry results, which, in our assessment, provided a satisfactory fit for our experimental data. As we highlighted above in response to a question to referee #1, we are pursuing high frequency and high field EPR measurements on Tb(IV)W_{34}, and the results of these efforts will be the subject of a follow-up study where insights into D-strain within the POM complex will be one of the focuses.

Action Taken: None

Given the drastic difference between the Gd(III) and Tb(IV) EPR (which is not the case for Eu(II) vs Gd(III) pairs in the same environment), I am not fully convinced yet that this is "evidence" of 4f7. Indeed the ChiT vs T trace has a significant temperature dependence that is not supported by S = 7/2 with D = 0.6 cm-1. Can they get the EPR spectrum of the Tb(III) complex before oxidation? Clearly they can get the magnetometry, so I would like to see what the EPR looks like - of course it might be silent, but it also might not be!

Our Response: We did collect X-band EPR data on Tb(III)W_{34} ahead of oxidation, and it is silent. La Pierre *et al*. (JACS, 2019, 141, 13222-13233) have observed a similar temperature dependence in their χT versus T data, which they attributed to variations in ZFS parameters, and this matches our findings as well. We agree that the changes between Eu(II) and Gd(III) complexes are known to be less pronounced, and Tb(IV) EPR measurements in host lattices such as ThO_2 and $ThSiO_4$ have also revealed more pronounced differences compared to Gd(III) (see Baker *et al*. Proc. R. Soc. Lon. Series A, 1965, 286, 352-365 and Hansen *et al*. Z. Naturforsch., A: Phys. Sci. 1996, 51, 885-894 for more details).

Action Taken: To add further clarity, consistent with the referee's suggestion we have updated two sentences in the EPR paragraph of the manuscript to read, "EPR is an ideal technique to detect the presence of Tb(IV) molecular species because the f^7 electron configuration of Tb(IV) results in readily detectable spectra at commercially available X-band frequencies, in contrast to f^8 Tb(III) complexes that are typically EPR silent. In this study, X-band EPR data were collected on Tb(III)W_{34} and Tb(IV)W_{34} at 5 K, **Figure 4A**. The Tb(III) complex was EPR silent as anticipated, while the Tb(IV)W_{34} spectrum

includes two distinct low field transitions at around 50 and 150 mT that are in agreement with the spectra for tetrahedrally coordinated Tb(IV) systems from La Pierre and Mazzanti.[18,19]"

Indeed, the curvature of the ChiT vs T trace is eerily similar to the Tb(III) sample. Could it be a mixture? I guess this is strongly supported by XANES (Fig 3). This needs to be discussed more "head-on" that this sample is likely a mixture under most analytical conditions.

Our Response: There is certainly a possibility that some percentage of Tb(III) is present as we highlighted in a response to referee #2 above, and we agree that under most analytical conditions Tb(IV) samples are likely a mixture Tb +4 and +3. The curvature of χT versus T for Tb(IV)W_{34} is consistent with other Tb(IV) examples in the literature (see La Pierre *et al*. JACS, 2019, 141, 13222-13233). Moreover, the effective magnetic moment value for Tb(IV)W_{34} is most consistent with expectations for a metal-ion with a $4f^7$ electron configuration. Coupled with the XANES results highlighted in Figure 3, we are comfortable with describing Tb(IV)W_{34} as a +4 complex, as it predominantly displays characteristics that would be expected of a tetravalent species. To reflect the uncertainty inherent in our measurements, we did not make further claims about our results, and we have also acknowledged the likelihood of reduction occurring during the sample preparation process in the following sentence in the SQUID magnetometry paragraph, "Effective magnetic moment values for both complexes are broadly consistent with expectations for Tb(IV) and Tb(III) complexes,[19] with the possibility of a small amount of reduction of Tb(IV)W_{34} occurring during the sample loading process ahead of measurement."

Action Taken: None

Please consider this letter as a response to additional comments from referee 2. While we disagree with their conclusion on the suitability of the manuscript for publication in Nature Communications, we have addressed each of the points they raised below, and in doing so we sought to find opportunities to improve our manuscript based on their feedback.

Reviewer(s)' Comments to Author:

Referee 2

The authors have made some attempt at improving the manuscript by integrating several comments that we put forward, especially regarding pH of the solutions. The authors have now provided the pH at which the reaction was carried out , and indicated that the pH value changes stay unchanged when the reaction is stirred in an open vial ruling out potential effects of pH change.

However, I often find the comments of the authors still rather dismissive with little action taken on the basis that instrumentation or capability to work under inert atmosphere are lacking.

Our Response: We must respectfully disagree with the referee's characterization that their original concerns were dismissed. The referee originally made nineteen suggestions about how the manuscript could be improved. We made fifteen of the suggested changes in the revised manuscript and provided detailed explanations for the four instances where updates were not possible or in our assessment did not contribute to improving the paper. We must also respectfully push back regarding the assumption that inert and aqueous synthesis conditions are equivalent. There are notable differences in how this work is conducted and at the core of the referee's feedback to the revised manuscript is the assumption that all laboratories have Schlenk lines or gloveboxes readily available. This is not the case, and in general all of the inorganic work conducted in our laboratory is done in ambient, aqueous conditions. As part of follow-up work on the title complex and with help from another group at the University of Iowa, we were able to run preliminary tests on the oxidation process and isolation of the Tb(IV) complex, and specific revisions made as a result of these findings are described in the points below.

Action Taken: None here, and see later points in this letter where we describe revisions that we have made as a result of preliminary synthetic tests conducted in inert environments.

Since the stabilization of Tb(IV) by polyoxometalates had already been reported together with stability studies I would expect more careful and thorough studies when rivisiting this old chemistry prior to resubmission of Nature communications.
In particular since the authors claim "that the compound undergoes autoreduction when exposed to air " this claim needs to be demonstrated by carrying the reaction in absence of air which should also allow to obtain a more pure product.
It is unacceptable to state that they cannot work under inert conditions (when working with unusual oxidation states this is not an option). Every laboratory can have access to a Schlenk line.

Our Response: We must respectfully disagree with the referee's characterization of earlier POM literature. The stabilization of Tb(IV) by POMs has not been previously reported, and notably, we did not use this descriptor either given the challenges we encountered during POM complex characterization. From our preliminary test experiments using a Schlenk line and in an Argon (water allowed) glovebox, we observed similar reduction behavior in single crystals of Tb(IV)W_{34} upon removal of the complex from the mother liquor. This does indicate that the sentence the referee has highlighted needs to be revised, and we have updated it accordingly. Moreover, this has provided insight that desolvation may also contribute to the instability of the POM complex, and we have included this information in the revised manuscript.

Action Taken: We have updated the sentence highlighted by the referee, which now reads, "We have overcome this limitation using polyoxometalate (POM) chemistry, and herein we present, $K_{16}Tb(IV)(\alpha_2\text{-}P_2W_{17}O_{61})_2\bullet 39.85H_2O$ (Tb(IV)W_{34}), a molecular tetravalent terbium complex that can be synthesized and characterized in water, although still susceptible to auto reduction upon desolvation."

Additionally, we have updated a sentence in the conclusion that had a similar focus. It now reads, "The LMCT band persists for over twelve days in water in a capped cuvette demonstrating aqueous stability of tetravalent terbium when air exposure and desolvation effects are minimized."

Finally, we have updated X-ray crystallography section (in the Methods) as well to ensure details provided throughout the manuscript are consistent. The relevant sentence in this section that highlighted effects of air on the Tb(IV)W_{34} crystals has been updated to read, "Crystals of Tb(IV)W_{34} are sensitive to desolvation and, after a period of approximately ten minutes, lose their brown color indicating the reduction of Tb(IV) back to Tb(III)."

It also seem strange that the authors were not willing to investigate what happens in the presence of stoichiometric amounts of K2S2O8 rather than 100 molar equivalent. One would expect more information from a paper that revisit past work. But this is even more surprising when one realizes that the final Tb(IV)W34 compound cannot be obtained pure because cannot be separated from the excess K2S2O8 . Separation would be easier if smaller amounts of K2S2O8 could be used.

Our Response: We must respectfully disagree with this characterization of our previous comment. As we highlighted in our first resubmission letter, the papers from Saprykin and Fedoseev included methods to generate Tb(IV) that required 2500-7500 equivalents of $S_2O_8^{2-}$. Here we achieved this same result using only 100 molar equivalents, which is a significant decrease. Ultimately the focus of the manuscript is on the characterization of Tb(IV)W_{34}, which was achieved by identifying a consistent, reproducible method for making the complex, and this is the procedure we have included in the methods section of the paper. The referee is requesting a separate study to figure out the minimum amount of $S_2O_8^{2-}$ required to obtain Tb(IV)W_{34}, and we think this is a worthwhile idea for a follow-up paper, as we highlighted in our first resubmission letter. Nevertheless, we ran a series of

test experiments using 10, 25, 50, and 75 molar equivalents of persulfate in analogous conditions, and based on these reactions at least 50 molar equivalents of persulfate is required to achieve the desired solution color change and to produce characteristic brown crystals. These solid products were not characterized in detail; however, the significant quantity of persulfate that is still required means the separation challenges we described in our initial resubmission letter still apply.

Action Taken: Follow-up test reactions were run to see how much persulfate is required for Tb oxidation, and qualitative observations indicate that at least 50 molar equivalents are needed to obtain Tb(IV)W_{34}.

The authors say "in ambient conditions, debulking K2S2O8 requires prolonged air exposure which leads to Tb reduction back to the +3 oxidation state" . This can be avoided by working in inert conditions. It really makes no sense and is not acceptable for a nature communication or any high level journal to prepare in air an air-sensitive compound!

Our Response: There is a lot to dissect in this comment and to do so we have included details from the initial resubmission letter and our responses that are directly related to this point.

The authors state: "After one day, colorless and brown crystals were observed. The colorless crystals were unreacted K2S2O8, while the brown crystals are Tb(IV)W34¬ (0.382 g, 39% yield)." How can a yield be established when the pure complex cannot be separated from unreacted K2S2O8? Have the authors obtained the complex analytically pure? This is very important.

Our Response: This is a good question and during the process of preparing the manuscript for submission this is one we thought about a lot. Due to the persistence of excess persulfate in Tb(IV)W_{34} samples, traditional methods for obtaining reaction yields were not possible. In our methods section, we aimed to provide complete details for each reaction, and so we designed a semi-quantitative process for determining the yield of the Tb(IV)W_{34} reaction based on data obtained from multiple measurements. This involved preparing fresh Tb(IV)W_{34} crystals, and then harvesting them, along with excess persulfate. The total weight of the mixture of products was determined and then the crystalline sample was ground thoroughly. Subsequently, we conducted energy dispersive X-ray spectroscopy on the Tb(IV)W_{34} sample to obtain the elemental ratios between sulfur and tungsten as well as sulfur and terbium. Based on these ratios, we can obtain a semi-quantitative yield of Tb(IV)W_{34} from the mixture of products. From there, we accounted for the amount of Tb(IV)W_{34} within the bulk crystalline product based on our SCXRD results. Tb-O bond distances in Tb(IV)W_{34} indicate that in the solid-state around 70% of the Tb within the crystals are in the +4 oxidation state, which provides us a conservative estimate of the amount of Tb(IV)W_{34} produced by each synthesis.

Action Taken: We have updated the relevant text in the methods section of the manuscript to read, "The colorless crystals were unreacted $K_2S_2O_8$, while the brown crystals are Tb(IV)W_{34} (54% yield (semi-quantitative)). The brown crystals were stored in the fridge (5

ºC) in a capped container filled with the mother liquor to slow down the auto-reduction process back to Tb(III)W_{34}. Worthy of additional comment, obtaining a yield value for Tb(IV)W_{34} is challenging due to co-crystallization with $K_2S_2O_8$. Gravimetric analysis and scanning electron microscopy-energy dispersive spectroscopy (SEM-EDS) were used to obtain the semi-quantitative yield value provided above. Details regarding this yield calculation can be found in the **Supporting Information**." Additionally, SEM-EDS results and yield calculation information have been added to the supporting information section where they can be found in **Figure S11**, **Table S6**, and in the paragraph on **pg. S26** titled, "**Tb(IV)W_{34} Yield Calculation Details**."

If is not the case, a separation should be pursued.

Our Response: We have spent a lot of time over the past month and a half thinking about this comment and pursuing potential separations pathways. A significant excess of $K_2S_2O_8$ is required for Tb oxidation and some of the oxidant crystallizes with the brown crystals of the Tb(IV) POM. In ambient conditions, debulking $K_2S_2O_8$ requires prolonged air exposure, which leads to Tb reduction back to the +3 oxidation state. The POM complex and $S_2O_8^{2-}$ are both anions, and they have different affinities for anion exchange resins as a result of their differences in charge. However, during the course of running a range of anion exchange columns, which in our lab involves extended exposure to ambient conditions, Tb was once again reduced back to the +3 oxidation state in all cases. Overall, this is a worthwhile line of investigation and we thank the referee for the suggestion. Based on what we have learned from attempting this separation during the course of the revisions process, isolating Tb(IV) in analytically pure form will require further optimization in terms of its coordination and solution stability with POM anions before this can be achieved, which is beyond the scope of this study.

Action Taken: None

Initially, we were asked to figure out if a separation of Tb(IV)W_{34} and persulfate was possible. We investigated this extensively, and provided details on why our efforts using multiple methods were unsuccessful. As a result, the referee has now indicated that an entire separations scheme in inert conditions needs to be designed and implemented, irregardless of whether this is reasonable or possible given facilities and equipment available in our laboratories. As we noted above, Schlenk lines and gloveboxes are not regularly available for inorganic synthesis in our laboratory at Iowa. We were able to run some test experiments related to a question from the referee in inert environments, with the help of collaborators, and based findings from these reactions we have updated the manuscript accordingly. Designing a separations procedure would first involve extensive characterization of inert products to confirm that the same species are obtained that we have isolated from ambient conditions, which would be very challenging considering the range of measurements and the specialized sample preparation that would be involved to collect air-sensitive XAS, EPR, or magnetometry data. Moreover, it would also likely require an extended work period in collaborators' laboratory spaces, and collectively, these are unreasonable peer review expectations, so they were not pursued. Worthy of a final comment, the aim of our efforts was to prepare a Tb(IV) complex in air. There are multiple

molecular examples of Tb(IV) complexes that can be isolated in inert conditions, and thus, moving in this direction for our work would, in our assessment, lessen the novelty and impact of our results.

Action Taken: None

In one of the answers to the question of purity a worrying note is made: Tb-O bond distances in Tb(IV)W34 indicate that in the solid-state around 70% of the Tb within the crystals are in the +4 oxidation state. This seems to indicate that even in isolated crystallin material not all the Tb are in the +4 oxidation state. This in my opinion reduces significantly the impact of the work and if the authors believe that this is due to performing the synthesis in air they should repeat it under inert conditions.

Our Response: We appreciate the feedback and respectfully disagree regarding the impact of the results. Differences in metal-oxygen or metal-nitrogen bond distances that are less than the expected ionic radii change between Tb(III) and Tb(IV) have also been noted by La Pierre (JACS, 2019, 141, 13222-13233) and Mazzanti (JACS, 2019, 141, 9827-9831) in their studies on air-sensitive molecular Tb(IV) complexes. The challenge in our study with fully realizing and capturing a 100% Tb(IV) species is a result of working in aqueous conditions, which is also the aspect of our work that is unique. The Tb(IV)/(III) redox couple is ~3.1 V vs. NHE, and aqueous POM complexation shifts this just below the redox accessible water window (to ~1.9-2.0 V). Consistent with the trajectory of studies focused on air-sensitive Tb(IV) complexes, we need to find a way to further cathodically shift the Tb(IV)/(III) redox couple, and this synthesis work is underway. Regarding the shift to inert conditions, to fully make this transition would also involve moving from water to an organic solvent, and this would completely change the POM chemistry we have described (and would likely require a different, non-tungsten based POM system).

Action Taken: None

It appers clearly now that the authors cannot obtain their complex in a pure form, but some amounts of Tb(III) are always present casting significant doubt on the reported characterizations.

Our Response: We appreciate the candid feedback, and without details on specific doubts it is not possible to address referee concerns on 'reported characterizations'. Notably, each of the characterization methods we used can provide oxidation state specific information and we have collected data on the Tb(III) analogue in all cases. Single crystal X-ray diffraction data showed that there was a significant difference in metal-oxygen bond distances between Tb(III) and Tb(IV) POM complexes. XANES spectra for Tb(IV)W_{34} featured a characteristic double white line that matched with the TbO_2 standard, in contrast to the single white line peak observed for Tb(III)W_{34}. UV-Vis-NIR spectra for Tb(III) and Tb(IV)W_{34} complexes with only the latter featuring a metal-to-ligand charge transfer transition at 437 nm (which we confirmed was specific to the Tb(IV) species using TD-DFT calculations). Continuous wave X-band EPR spectra revealed an isotropic signal for Tb(IV)W_{34}, which is expected for a species with a $4f^7$ electron configuration, while the

Tb(III)W_{34} spectrum was silent, and SQUID magnetometry results revealed clear differences between the two complexes, where the Tb(IV)W_{34} complex featured an effective magnetic moment value very close to the that of Gd(III) rather than Tb(III). As such, we have acknowledged our synthetic limitations, and investigated them further as part of this second resubmission, and we are confident in our characterization of the Tb(IV)W_{34} species.

Action Taken: None

How the isolation of the title compound in pure form could be "beyond the scope of this study"?

Our Response: We must respectfully push back against this out of context comment. We used the phrase, "beyond the scope of this study," in addressing a separations related point in the previous resubmission letter (full details are provided herein as part of our response to an earlier comment from the referee). Our initial attempts at separating Tb(IV)W_{34} from excess persulfate were unsuccessful due to a range of experimental logistical hurdles that led to consistent Tb reduction. As a result, we indicated that synthesis optimization would be required, as well as multiple additional experiments, and this was a worthwhile topic for a follow-up study (as this would facilitate potential applications of the POM chemistry we have outlined herein). The focus of this manuscript is on the characterization of the Tb(IV)W_{34} POM and as we have outlined in the point above, the techniques used are not impacted by the presence of excess persulfate. We agree with the referee that 'isolation' is likely the wrong word to use here (see more details in a point below), and we have revised the manuscript title and a relevant sentence in the abstract so this word is no longer included.

Action Taken: None here and see details below on how we have made updates for clarity related to the use of the word 'isolation' in the manuscript title and abstract.

I find the use of the term 'isolation' potentially misleading as it could lead the reader to think that Tb(IV)W34 can be obtained pure, with no (or negligible) amounts of Tb(III) and persulfate, which is not the case.
Finally the key result here was announced to be the isolation of a water stable Tb(IV) complex but in their reply now they seem to agree that water is likely to act as reductant (not oxygen) as was suggested in the early Fedoseev studies and therefore an excess of persulfate oxidizing agent is always required to keep the complex in the +IV oxidation state.

Our Response: It is a reasonable point that isolation may not be the best word to use in the title of the manuscript, so we have updated it to, "**Structural and Spectroscopic Characterization of a Tb(IV) Polyoxometalate**." There were also one instance where we used isolation in the paper, which we have revised. Overall, we have provided definitive evidence that we can obtain a POM complex that displays structural and spectroscopic characteristics consistent with a species in the +4 oxidation state. This complex persists in solution, in the presence of excess persulfate, and the means for Tb reduction warrants

further study. There is more to the reduction process than was outlined by Saprykin or Fedoseev, and with more extensive characterization methods now available, this is an area we are working to advance.

Action Taken: The title of the manuscript was updated based on feedback from the referee and we have updated one sentence in the manuscript abstract to ensure greater clarity in the text. It now reads, "In this investigation, we have advanced this paradigm by obtaining definitive evidence of Tb(IV) in a molecular system under aqueous conditions utilizing the lacunary Wells-Dawson polyoxometalate, $K_{10}P_2W_{17}O_{61}$•$20H_2O$."

Notably in reply to our request to establish the stability of pure Tb(IV)W34 in water , they report stability in presence of persulfate.
I do not think the manuscript in its current form is suitable for publication in Nature Communication and this manuscript could be published in an Inorganic Chemistry journal as it is but clearly stating the limitations of the study (no pure compound obtained, stability of the complex only in presence of persulfate, uncertainty on the origins of the low stability in air).

Our Response: We must respectfully disagree with this conclusion. As highlighted earlier in this letter, we addressed 15/19 points originally provided by the referee in our revised manuscript. As part of these efforts, we conducted multiple additional experiments and worked to design separations processes to address referee questions. Research does not always yield ideal answers, and we acknowledged in our previous resubmission letter that persulfate is likely acting as a stabilizer for Tb(IV)W_{34}, akin to stabilizing agents added to reactive species such as H_2O_2. We judiciously selected characterization methods that were element or electronic configuration specific, so we could confirm Tb(IV) character even in the presence of $S_2O_8^{2-}$. Advancing synthesis and characterization methods for metals in unusual oxidation states is an area of broad interest within the chemical and physical sciences, and we agree with referees 1 and 3 that this is a manuscript worthy of publication in Nature Communications.

Action Taken: None

Please consider this letter as a response to additional comments from referee 2. We havefur ther revised the manuscript to address each of the points they raised below, and in doing so we sought to find opportunities to improve our manuscript based on their feedback.

Reviewer(s)' Comments to Author:

Referee 2

The authors have made several amendments to the manuscript, clarifying that the title compound cannot be isolated in pure form, though the underlying reason remains unclear. While I agree that stabilizing a Tb(IV) polyoxometalate (POM) in water is an important result, several critical questions remain. Below are my responses to the authors' rebuttal:

1. The authors claim that the stabilization of Tb(IV) by POMs has not been previously reported. This is incorrect. As the authors themselves acknowledge in the text, Saprykin et al. documented the solution-state complexation of Tb(IV) with W17 ligands nearly half a century ago, and Fedoseev's group has also pursued this chemistry. However, I fully recognize and commend the authors for utilizing this foundational knowledge to isolate single crystals of Tb(IV) and further characterize them, despite the characterizations being carried out on non-analytically pure compounds.

Our Response: We have not made the claim that stabilization of Tb(IV) POMs has not previously been reported as evidenced by the following sentence in the introduction section of the manuscript, "We are not the first to recognize the potential of W_{17} POMs for lanthanide coordination chemistry efforts as Saprykin *et al*. have documented the solution state complexation of Tb(IV) with W_{17} ligands almost half a century ago.[39]" We are appreciative of the comments though and we thank the referee for the detailed feedback they have provided here and in previous rounds of revisions as they have helped to improve the manuscript.

Action Taken: None

2. The authors note that their inorganic work is conducted in ambient, aqueous conditions due to a lack of gloveboxes or Schlenk lines. While we respect that not all laboratories possess this specialized equipment, tackling air-sensitive chemistry inherently requires the appropriate conditions and equipment to handle it properly. Furthermore, the new experiments the authors carried out in an argon atmosphere confirmed that the complex does not undergo autoreduction in air. Therefore, the authors should remove mentions of the complex being "air-sensitive" from the manuscript, as this has been disproved.

Our Response: While we acknowledge that use of a glovebox or Schlenk line could have made aspects of the chemistry described within the manuscript easier, our aim was actually to explore whether Tb(IV) could be accessed in ambient conditions, even if this presented

additional experimental challenges. We agree that describing the Tb(IV) POM as air-sensitive is incorrect and have updated the manuscript accordingly.

Action Taken: The only remaining mention of the POM being air-sensitive was in the conclusions and we have the updated relevant sentence to read, “The LMCT band persists for over twelve days in water in a capped cuvette demonstrating aqueous stability of tetravalent terbium when desolvation effects are minimized.”

3. A serious problem remains: the complex cannot be isolated as a pure compound, but only in the presence of K2S2O8. . (This is often not accepted in high-level journals). Importantly the presence of K2S2O8. . raises some questions: the authors attribute the low stability to various factors (solvation, autoreduction in the absence of oxidants, and air-sensitivity), creating confusion in the manuscript. It is highly possible that the complex is stable only in the presence of an excess of the oxidizing agent. This can only be proven by removing the excess oxidant and testing if the resulting clean crystals are stable in pure water.

Our Response: We have updated the manuscript to ensure that Tb(IV)W_{34} stability limitations are clearly and consistently described. Further discussions related to how data was collected (i.e., from isolated single crystals of the POM or those containing excess persulfate) is included below, and specific information for each experimental measurement is now explicitly included within the methods section of the manuscript.

Action Taken: None here and see below for specifics on how referee concerns were addressed based on their specific recommendations for updates to the manuscript.

4. Suggested Revisions to the Manuscript
To proceed with publication, the authros should make the following revisions:

Clearly state in the manuscript that the Tb(IV) species cannot be isolated without the presence of K2S2O8. This must be explicitly stated in the main text, not just hidden in the methods section.

Our Response: We have updated the manuscript consistent with the suggestion of the referee.

Action Taken: The following sentence in the first paragraph of the introduction has been updated, “We have overcome this limitation using polyoxometalate (POM) chemistry, and herein we present, $K_{16}Tb(IV)(\alpha_2\text{-}P_2W_{17}O_{61})_2 \cdot 39.85H_2O$ (Tb(IV)W_{34}), a molecular tetravalent terbium complex that can be synthesized in the presence of persulfate and characterized in water, although still susceptible to auto reduction upon desolvation.”

Additionally, we have updated the first sentence of the conclusions to read, “In this investigation, Tb(IV)W_{34} and Tb(III)W_{34} complexes have been synthesized and characterized via multiple spectroscopic techniques to prove that Tb(IV) can be isolated and characterized in aqueous media in the presence of persulfate.”

Clarify Crystal Separation: Indicate whether and when the Tb(IV) crystals were manually separated from K2S2O8. prior to spectroscopic characterization.

Our Response: Single crystal X-ray diffraction data was collected on individually selected Tb(IV) POM crystals and the relevant paragraph in the methods section already includes this information. UV-Vis-NIR and XAS measurements on the Tb(IV) POM used samples that contained persulfate, and these results were not affected by its presence. EPR and SQUID magnetometry data collections were performed using single crystals that were isolated from persulfate before measurement. This information was already included in the SQUID magnetometry paragraph of the methods section, and we updated the EPR paragraph to explicitly convey how the sample was isolated ahead of data collection.

Action Taken: The sentence in the EPR paragraph of the methods section related to sample preparation for Tb(IV)W_{34} has been updated to read, “Samples of Tb(IV)W_{34} analyzed using EPR spectroscopy were prepared by slow evaporation from a 5 mM solution, and crystals were separated from persulfate and subsequently harvested with a transfer pipette along with the mother liquor.”

Specify Storage Conditions: In the Methods section, specify that the capped container used to store the brown crystals was capped under air.

Our Response: We have updated the relevant methods section sentence based on referee feedback.

Action Taken: In the ‘Synthesis of $K_{16}[Tb(IV)(P_2W_{17}O_{61})_2]\cdot xH_2O$**’** paragraph within the methods section, we have updated the sentence highlighted by the referee to read, “The brown crystals were stored in the fridge (5 ºC) in a capped under air container filled with the mother liquor to slow down the auto-reduction process back to Tb(III)W_{34}.

Incorporate the experiments mentioned in the reply into the manuscript. Specifically, add that experiments using a Schlenk line and an argon glovebox showed similar reduction behavior in single crystals upon removal from the mother liquor. Please clarify this observation: are the crystals redissolved in pure water, or do they undergo reduction immediately upon removal from the solution?

Our Response: We have added the requested information regarding experiments conducted with Tb(IV)W_{34} using a Schlenk line and an argon glovebox. Crystals of Tb(IV)W_{34} are reduced upon removal from solution, although this process is not immediate, which is why we were able to obtain XRD, XAS, EPR, and SQUID data on the complex while mounting or loading samples for the measurements in ambient environments.

Action Taken: The ‘Synthesis of $K_{16}[Tb(IV)(P_2W_{17}O_{61})_2]\cdot xH_2O$’ paragraph has been updated to include information requested by the referee. Specifically, the following sentence has been added, “Synthesis of Tb(IV)W_{34} was also pursued using Schlenk

techniques and in an argon glovebox, and storage of the POM crystals in sample mother liquor was still required to limit auto-reduction to the Tb(III) analogue."

Clearly discuss whether clean crystals, if separated, maintain their stability in pure water. Refrain from describing this as an "air-sensitive" compound, as the authors' own data disproves this behavior.

Our Response: As we have highlighted in previous response to reviewers letters, separating persulfate from Tb(IV)W_{34} requires some amount of time where the crystals are outside of the mother liquor, and during this time auto-reduction does occur. We agree that use of the term air-sensitive is inaccurate, and as noted in a point above, we removed the one mention of Tb(IV)W_{34} sensitivity that remained in the manuscript.

Action Taken: Consistent with an earlier point made by the referee we updated the sentence in the conclusions section that included a mention of POM air-sensitivity to read, "The LMCT band persists for over twelve days in water in a capped cuvette demonstrating aqueous stability of tetravalent terbium when desolvation effects are minimized."

Note: I will not oppose publication if the Editor is satisfied with reporting a non-analytically pure compound, provided the aforementioned limitations are transparently disclosed to the reader.

Our Response: We appreciate the feedback and have made the further revisions requested by the referee.

Action Taken: None here and see the earlier points in this letter where we describe updates we have made to the manuscript based on referee feedback, with the aim of providing greater clarity and transparency.
